# Unveiling the Structural Modifications of Cyanines to Target G‑Quadruplex DNA through Biophysical, Computational, and Transcriptome Analyses

**DOI:** 10.1021/acsomega.5c11600

**Published:** 2026-05-15

**Authors:** Cristina Galiana-Roselló, Andrea Lázaro-Gómez, Ariadna Gil-Martínez, Anargyros Drolapas, William E. Meador, Catalina Nicolau, Chun-Qiong Zhou, Jared H. Delcamp, Antonio Bauza, Jorge González-García

**Affiliations:** † Department of Inorganic Chemistry, Institute of Molecular Science (ICMol), 16781 University of Valencia, Catedrático José Beltrán 2, Paterna 46980, Spain; ‡ Príncipe Felipe Research Center, Eduardo Primo Yúfera, 3, Valencia 46012, Spain; § Materials and Manufacturing Directorate (RXNC), 33319 Air Force Research Laboratory, 2230 Tenth Street B655, Wright-Patterson AFB, Ohio 45433, United States; ∥ Department of Chemistry, 16745 Universitat de les Illes Balears, Crta de Valldemossa Km 7.5, Palma de Mallorca, Baleares 07122, Spain; ⊥ School of Pharmaceutical Sciences, 70570 Southern Medical University, Guangzhou 510515, P. R. China

## Abstract

Six cyanine ligands differing in the polymethine linker and the substituents have been developed and investigated as binders for DNA. The interaction with DNA was assessed by fluorescence resonance energy transfer (FRET) melting, UV–vis, and fluorimetric assays and completed with computational experiments. The ligand containing a shorter linker of one carbon length shows no interaction, while increasing the linker length enhances the interaction toward G-quadruplex DNA structures. The largest binding is observed for the cyanines containing five carbon connectors, while the change of the electronic nature of the substituents has no significant effect on the binding to G4s. The binding modes show a preference for the *cis* conformation of the cyanines, which overlaps more efficiently with the G-quartet through π–π interactions. Whole-transcriptome RNA-seq analysis shows the global gene expression in HeLa cells treated with the strongest and most selective G4 ligand, **C3**. The changes produced by **C3** represent a global response to a complex mechanism, which sheds light on the cellular activity of cyanines.

## Graphical abstract

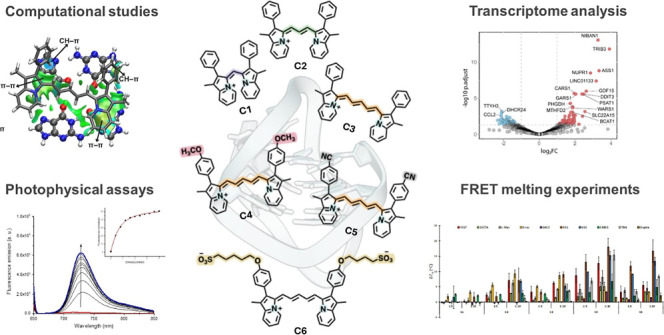

## Introduction

Cancer remains the second leading cause of morbidity and mortality worldwide, causing nearly 10 million deaths in 2020.^1^ In consequence, researchers and clinicians are constantly seeking novel therapeutic and diagnostic approaches to improve patient outcomes.^2–4^ Among current advances, immunotherapy and precision medicine are at the forefront of the clinical advances fostered by the identification of genetic and epigenetic alterations in cancer cells.^5,6^ One of the most promising epigenetic targets in cancer therapy is the G-quadruplex (G4) DNA structure, described together with other noncanonical DNA/RNA structures in the past decade.^7–9^ G4s are formed in guanine-rich sequences by the hydrophobic stacking of coplanar G-tetrads, stabilized by the hydrogen bonding network of four guanine bases. The structure is assembled by alkali metal cations, and its stability depends on additional factors such as the number of G-tetrads and the length of the loops.^10–12^

The biological relevance of G4s in cancer arises from the presence of putative G4-forming sequences in telomeres and in the promoters of several oncogenes.^13^ The formation and/or stabilization of G4 structures within these regions by small ligands has therefore emerged as a potential anticancer strategy.^14–16^ Indeed, the G4-stabilizing fluoroquinolones CX-5461 and CX-3543 (or quarfloxin) have reached clinical trials. CX-5461 displays a complex mechanism involving the stabilization of G4s in *cMyc* and *cKit* promoters and in telomere regions, as well as replication fork blockages, ultimately leading to DNA damage and inhibition of rRNA biogenesis.^17^ Currently, CX-5461 is in phase I clinical trials for patients with BRCA1/2-deficient tumors.^18^ On the other hand, CX-3543 targets ribosomal G4s in the nucleolus, inhibiting RNA polymerase and causing reduction of the tumor volume in pancreatic cancer xenograft models.^19^ Nevertheless, it has been withdrawn because of bioavailability issues and insufficient efficacy.

In parallel to the development of therapeutic ligands, the design of G4 probes has provided tools for investigating G4s in vitro and in cells.^20–22^ Most of the G4 probes rely on the fluorescence enhancement upon DNA binding, although other detection strategies based on the fluorescence lifetime emission have been developed.^23–25^ Moreover, very few antibodies/nanobodies are available but remain scarce for the fixed-cell G4 recognition.^26,27^ A complete understanding of G4 biological functions thus requires reliable probes for their detection and visualization in cellular processes, emphasizing the need for simple and commercially available tools.

A myriad of small molecules has been described as G4 binders and probes, including organic ligands (i.e., acridines, perylenes, anthraquinones, porphyrins, etc.)^28–32^ and metal complexes (salphens, terpyridines, porphyrins, metalophthalocyanines, etc.).^33,34^ Among them, several cyanine ligands have been reported as G4 binders and probes due to their structural, electronic, and photophysical properties, complementing the unique G-quadruplex structure and properties.^35–37^ Cyanines are characterized by two heterocycles containing nitrogen atoms, which are connected via a polymethine bridge, forming an extended, planar π–conjugated scaffold. This planar aromatic surface enables efficient π–π stacking interactions with the G-tetrads of the G4s. Moreover, cyanines are attractive dyes due to their favorable optical properties, such as high quantum yields, accessible synthesis, tunable water solubility, and emission in the biological window.^38^ Cyanines likely exhibit low fluorescence in aqueous solution but undergo an increase of the emission upon binding biomolecules (proteins, DNA, etc.) because of the restriction of intramolecular rotation and suppression of nonradiative decay pathways, making them valuable as “*light-up*” probes for detection and imaging. Nevertheless, the development of cyanine ligands as G4 ligands has been challenging over the years. The high flexibility of the polymethine moiety favors its binding to duplex DNA grooves, decreasing the specificity for G4.^39^ An efficient strategy has been to incorporate substituents with steric restraints to reduce the duplex interaction.^40^ Wilson and collaborators developed symmetric cyanines with steric hindrance and asymmetric cyanines containing a planar heterocyclic moiety to avoid duplex interaction and to facilitate G-quartet π-stacking, respectively.^41,42^ In this context, Delcamp and collaborators developed a series of indolizine cyanines showing a near-infrared emission with large quantum yields and high molecular brightness.^43^ They investigated the incorporation of water-solubilizing groups, such as sulfonate, to afford photostable dyes able to detect albumin with a lower limit of detection than known dyes.^44,45^

In the present study, we explored a portfolio of cyanine ligands differing in the polymethine linker and heterocycle substitution. The aggregation tendency of six indolizine cyanine ligands was initially assessed by UV–vis spectroscopy and dynamic light scattering (DLS). Then, the cyanine-G4/ds DNA interaction was studied by fluorescence resonance energy transfer (FRET) melting and UV–vis/fluorescence spectroscopies. We additionally investigated their binding to G-quadruplex structures by using theoretical studies. We then evaluated the cell activity of the most promising cyanine as a G4 ligand (**C3**) in cells by whole-transcriptome RNA-seq analysis.

## Experimental Methods

All reagents were obtained from commercial sources and used without further purification, unless otherwise noted. Organic solvents were dried by keeping them over molecular sieves 4 Å. The unlabeled and labeled DNA oligonucleotides were purchased from IDT DNA as HPLC grade, and the labeling dyes were 5′-FAM and 3′-TAMRA (see Table S1 for sequences). Ligands were dissolved in Milli-Q water to give 5 mM stock solutions. All solutions were stored at −20 °C and defrosted and diluted immediately before using a suitable buffer to the appropriate concentrations.

### Aggregation Studies

For the DLS experiments, aqueous suspensions were prepared by diluting the stock solution of each cyanine with Milli-Q water, reaching a final concentration of 5 μΜ. The suspensions were vortexed for 60 s and transferred to a plastic cuvette for measuring via dynamic light scattering (DLS) using a ZetaSizer Nano ZS (Malvern Instruments, UK). Each measurement was conducted at 25 °C in backscattering mode (173 °), and the results were averaged over 3 repetitions.

### FRET Melting Assay

Labeled DNA was dissolved as a 20 μM stock solution in Milli-Q water and then annealed at a 400 nM concentration in potassium cacodylate buffer (LiCac 10 mM, 10 mM KCl, 90 mM LiCl, pH 7.3) at 95 °C for 5 min and allowed to cool slowly to 25 °C overnight. Ligands were dissolved from stock solutions (see above) to final concentrations in the buffer. Each well of a 96-well plate (Applied Biosystems) was prepared with 60 μL, with a final 200 nM DNA concentration and increasing concentrations of tested ligands (0–4 μM). Measurements were performed on a PCR AriaMx (Agilent Technologies) with excitation at 450–495 nm and detection at 515–545 nm. Readings were taken from 25 to 95 °C at an interval of 0.5 °C, maintaining a constant temperature for 30 s before each reading. Each measurement was done in triplicate. The normalized fluorescence signal was plotted against the temperature, and the Δ*T*_m_ values were determined.

For the competition FRET melting assay, labeled oligonucleotides were annealed at concentrations of 400 nM. Ligands were diluted from stock solutions in the same buffer as the DNA to yield the final concentrations. Each well of a 96-well plate was prepared with a final oligo concentration of 200 nM, a ligand concentration of 2 μM, and the *ds26* concentration to test. Measurements were performed under the same conditions as the FRET melting assays.

### UV–Vis/Fluorescence Titrations

The DNA was dissolved in *Tris* buffer (*Tris* 10 mM, KCl 100 mM, pH 7.4) and annealed at 95 °C for 10 min before cooling to room temperature overnight. The concentration of DNA was checked using the molar extinction coefficients provided by the manufacturer. Annealing concentrations were approximately 500 μM. For the titrations, ligands (5 μM) in buffer were titrated with the corresponding DNA until saturation of absorption. The absorption spectra were recorded on a Varian Cary UV–vis 100 spectrometer. The fluorescence emission spectra were registered on a Varian Cary Eclipse spectrometer. Spectra were smoothed using the Savitzky–Golay algorithm, and absorption maxima were fitted to binding models using the Levenberg–Marquardt algorithm and equations previously reported.^46,47^

### Theoretical Models

The energies were computed at the B3-LYP^48,49^ /def2-TZVP^50^ level of theory by means of the TURBOMOLE 7.7 software.^51^ The interaction energies were calculated following the supermolecule approximation, that is, Δ*E* = *E*_G4···ligand complex_ – *E*_G4_ – *E*_ligand_. Solvent effects were treated by means of the COSMO (Conductor-like Screening Model) continuum model implemented in TURBOMOLE software, using water as the solvent.^52^ To obtain the interaction energies gathered in Tables S3–S12, we first optimized the G4 quartet structure and then kept it frozen and relaxed compounds **C1** to **C5** over it. Once the final geometry was obtained, we carried out single-point calculations at the B3-LYP/def2-TZVP level of theory in water.

The Molecular Electrostatic Potential (MEP) surfaces were calculated at the same level of theory using the Gaussian 16 calculation package.^53^ Lastly, the NCIplot^54^ isosurfaces correspond to both favorable and unfavorable interactions, as differentiated by the sign of the second density Hessian eigenvalue and defined by the isosurface color. The color scheme is a red–yellow–green–blue scale with red for repulsive (ρ^+^cut) and blue for attractive (ρ^–^cut) NCI interaction density. Yellow and green surfaces correspond to weak repulsive and weak attractive interactions, respectively.

### Molecular Docking

The crystal structure of the DNA-ligand complex was downloaded from RCSB Protein Data Bank (PDB code: 296D).^55^ The original cocrystallized ligand and water molecules were removed by Chimera 1.16.^56^ The structures of the ligands were created and optimized with Avogadro^57^ at the MMFF94s Force Field. After that, the structures were imported into AutoDockTools-1.5.7,^58^ and hydrogen atoms were added. A grid box encompassing the duplex was used to enable the blind docking to be carried out. The same grid coordinates were used for all cyanines. Docking was performed using AutoDockVina 1.1.2.^59,60^ Input comprises the receptor, ligand, and docking box, while output is a list of poses ranked by Δ*E*, the predicted binding energy in kcal/mol (see Table S13). To obtain the maximum number of poses, we set *num_modes* to 24 and *energy_range* to 10. The docking models with the lowest docked free energy were selected, and the docked structures were visualized using Chimera 1.16 and PyMOL.^61^

### Cell Culture and Maintenance

All cell lines were obtained from the American Type Culture Collection (ATCC). Human cervical (HeLa-CCL-2), breast (MCF7-HTB-22), and lung (A549-CCL-185) carcinoma and murine macrophage (Raw 264.7-TIB-71) cell lines were maintained in DMEM high glucose (4.5 g/L) with l-glutamine (GIBCO) supplemented with 10% heat-inactivated fetal bovine serum, 1% penicillin/streptomycin (100 units/ml), and 0.1% Fungizone (GIBCO). Cells were grown subconfluently in a humidified incubator at 37 °C with 5% CO_2_ and passaged, routinely tested for mycoplasma contamination, and subjected to frequent morphological examination and growth curve analysis as quality control assessments.

### Drug Dose–Response Viability Assays

Stock solutions of indolizine cyanine derivatives were prepared in DMSO at a concentration of 10 mM and stored in aliquots at −20 °C. HeLa, MCF7, and A549 cells were seeded at a density of 5000 cells/well in 96-well plates, while Raw 264.7 cells were seeded at a density of 10.000 cells/well. After 24 h of attachment, treatments were performed as 1:2 serial dilutions from a maximum concentration of 25 or 100 μM. Cell viability after 48 h of treatment was determined based on the quantification of ATP, which signals the presence of metabolically active cells, using the Cell Titer-Glo luminescence assay kit (Promega, Madison, WI). Cell Titer-Glo reagent was added to the cells according to the manufacturer’s instructions, and luminescence was read using the BioTek Synergy H1Multimode Reader. Samples were normalized to untreated controls, and dose–response curves and IC_50_ values were determined using GraphPad Prism 6 software.

### RNA-Seq Studies

RNA-seq analyses of exposure to **C3** were undertaken with HeLa cells. HeLa cells (4 × 10^6^ cells/well) were seeded in 60 mm plates. The following day, cells were treated with 40 nM **C3** for 24 h. Total RNA was extracted using the Qiagen RNeasy Plus Mini Kit (ThermoFisher, catalogue no. 74134) as per the manufacturer’s instructions. RNA quality (RIN >9.0) was checked with an Agilent 2100 bioanalyzer RNA 6000 Nano Chip, and RNA concentration was quantified using a Qubit fluorometer (ThermoFisher) and Qubit RNA HS Assay Kit (Qiagen, catalogue no. Q32852).

### RNA-Seq Library Preparation and Sequencing

RNA libraries were prepared following standard protocols for 3′-end transcript enrichment. Briefly, reverse transcription was initiated using a combination of random primers and oligo­(dT) primers to ensure coverage across transcripts while capturing the polyadenylated tail. The resulting cDNA fragments were subjected to an initial PCR amplification to increase template availability, followed by a second PCR step to introduce sequencing adapters required for cluster generation and downstream sequencing.

Library quality was assessed with the QIAxcel Advanced System (Qiagen), which provides fragment size distribution and allows the detection of potential degradation or insufficient concentration prior to sequencing. Paired-end sequencing (2 × 150 bp) was performed on the Illumina NovaSeq X platform using sequencing-by-synthesis (SBS) chemistry. Fluorescently labeled nucleotides were incorporated sequentially and detected in real time, generating millions of short reads.

### Bioinformatic Analyses

*Quality control and trimming*: Raw sequencing reads were subjected to adapter removal and quality trimming, including poly­(A) sequence clipping, using standard preprocessing workflows. Read quality was evaluated with *FastQC* (v0.11.9) to confirm high per-base quality scores, the absence of adapter contamination, and minimal low-quality bases. *Read alignment*: Cleaned reads were mapped to the human reference genome (GRCh38) using STAR (v2.7.10) with splice-aware alignment. PCR duplicates were removed based on unique molecular identifiers (UMIs) with UMI-tools to retain only unique fragments. *Gene quantification*: Gene-level counts were generated using HTSeq-count (v2.0.2) with NCBI gene annotations. To correct for differences in sequencing depth and sample composition, raw counts were normalized with the median-of-ratios method implemented in *DESeq2*.^62^ *Differential expression analysis*: Differential gene expression between groups was assessed using *DESeq2*. *P*-values were adjusted for multiple testing with the Benjamini–Hochberg procedure to control the false discovery rate (FDR). Results were visualized using principal component analysis (PCA), dispersion estimates, volcano plots, and heatmaps of top differentially expressed genes. Summary tables included log2 fold changes, Wald statistics, and raw and adjusted p-values. *Functional enrichment analysis*: Gene Ontology (GO) and Kyoto Encyclopedia of Genes and Genomes (KEGG) pathway enrichment analyses were performed using *clusterProfiler* (v4.12.0). Genes were ranked by the Wald statistic, and enrichment scores were calculated with multiple-testing correction by the Benjamini–Hochberg method. To reduce semantic redundancy among enriched GO terms, significant Biological Process categories were clustered by similarity using the Revigo R package (v1.12.2), which groups functionally related terms based on semantic similarity (Rel method, threshold = 0.7, *org.Hs.*eg*.db*). Representative parent terms for each cluster were selected based on minimal dispensability or maximum enrichment score to facilitate interpretation.

## Results and Discussion

### Design and Photophysical Properties of Indolizine Cyanine Ligands

Six cyanine ligands containing two indolizine units were synthesized and characterized (Figure 1) as reported previously.^43,44^ The indolizine units are connected through a polymethine unit differing in the carbon length of one, three, or five carbon atoms. Additionally, the larger cyanine **C3** was derivatized with electron-donating/-withdrawing groups, methoxy and cyanine groups, respectively, and sulfonate groups showing two negative charges.

**Figure 1:**
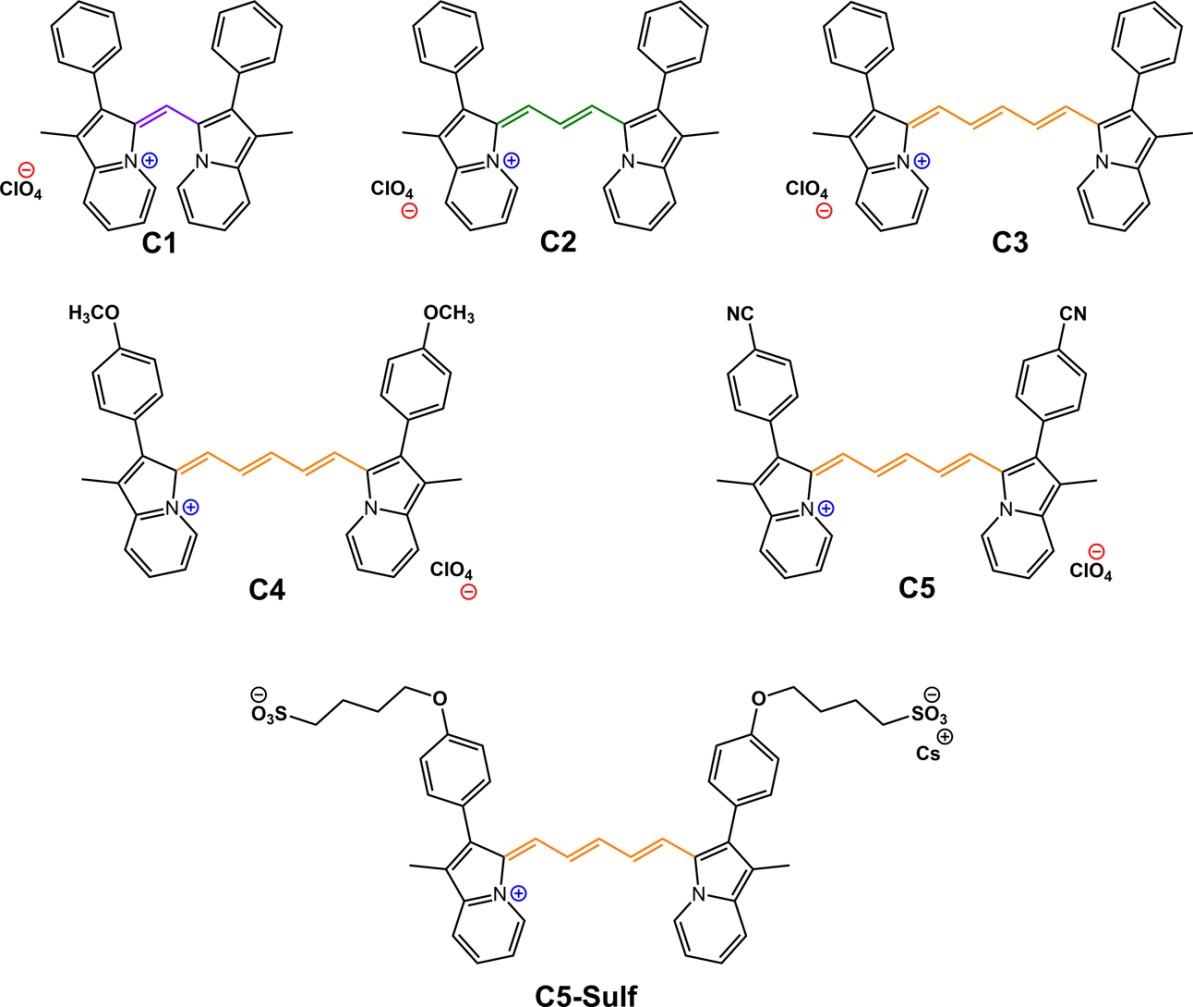
Structures of the cyanine ligands studied in this work.

We initially studied the photophysical properties (absorbance and fluorescence emission) of the cyanine ligands in solution with concentration, solvent polarity, and temperature. All the ligands showed a large enhancement of the fluorescence emission in nonpolar solvents such as toluene, in contrast to the low emission in water.^44,45^ This effect can be attributed to the nonemissive aggregate state of the ligands in the most polar solvent, which is disassembled in apolar solvents. With regard to the UV–vis spectra, a red shift occurred from polar to nonpolar solvents (water–methanol–dioxane–toluene), indicating that the monomer cyanines are associated with the red-shifted UV–vis band.

To study the potential ability of the ligands to self-aggregate in aqueous conditions, we monitored the UV–vis spectra at different concentrations ([L] = 5–100 μM) in *Tris* buffer. The absorption intensity was nonlinear above 20 μM for **C2**, **C4**, and **C5**, indicating that these ligands highly aggregate in an aqueous environment at these conditions, in contrast with **C1** and **C3**, which showed a linear dependence on the ligand concentration (Figures S1–S6).

We then recorded the absorption spectra at different solvent ratios of H_2_O/MeOH mixtures (Figures 2 and S7–S11). The initial Vis band in 100% water of **C2**–**C5** ligands was red-shifted and showed a hyperchromic effect, indicating a change from an aggregated state in water to a monomer state in 100% methanol. We calculated the percentage of methanol to swap between states at around 70:30 H_2_O/MeOH. The absence of change in the photophysical properties for **C1** confirmed the impossibility of disassembling in solution because of the short linker of the cyanine, causing a stable aggregate form. We observed a change in the absorption bands upon increasing the temperature from the aggregated to the monomeric forms because of the breaking of the intermolecular forces that hold the aggregates. Only cyanine **C2** showed a large enhancement of the fluorescence when the ligands changed from pure water to pure methanol, indicating that the emissive state of the ligands corresponds to the monomer (Figure S12).

**Figure 2:**
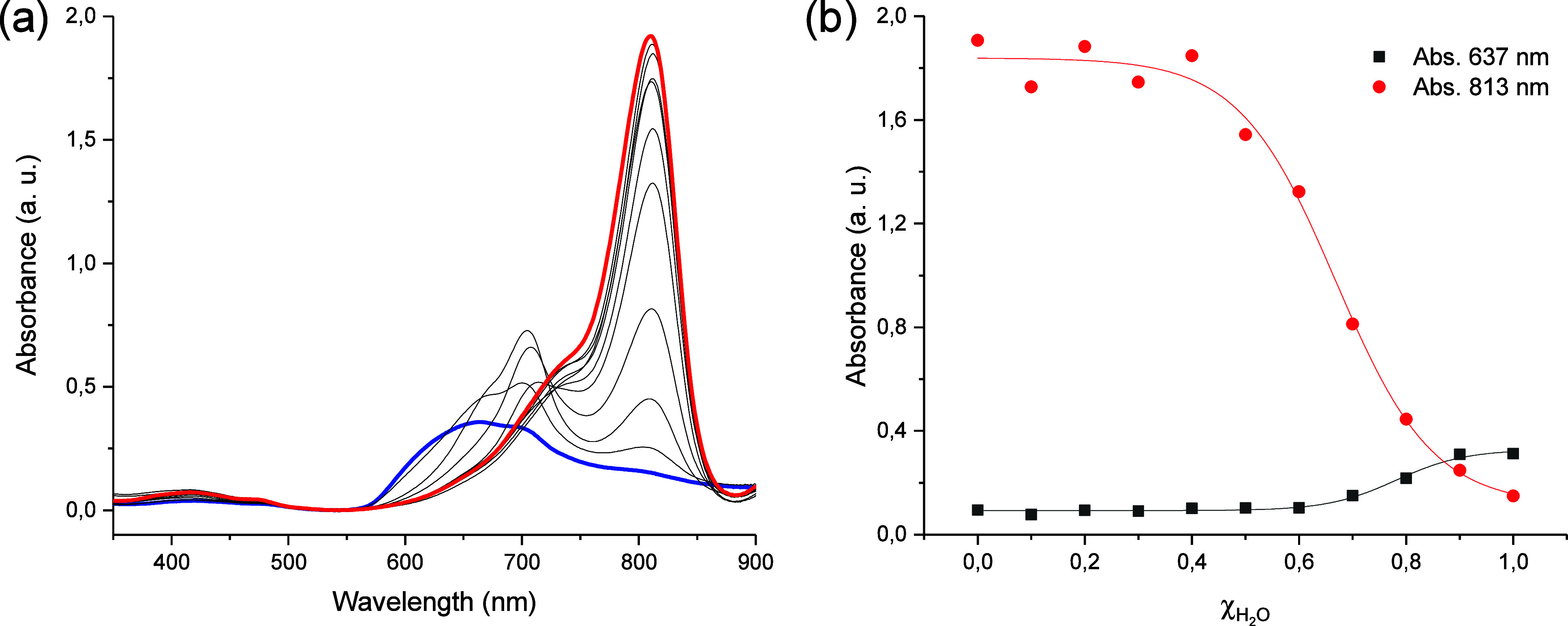
(a) UV–vis spectra of **C3** in different solvent mixtures (blue line: 100% H_2_O, red line: 100% MeOH). (b) Absorbance at 637 and 813 nm versus the percentage of H_2_O (χ_H_2_O_).

We confirmed this effect for cyanine **C2** by monitoring the UV–vis spectra over the temperature in a mixture of 80:20 H_2_O/MeOH, showing the change of the UV–vis bands from the aggregate to the monomer species (Figure S13).

To get insights into the aggregates in solution, we performed DLS experiments in pure water, and **C1**–**C5** obtained aggregates ranged from 90 to 360 nm in size (Table S2 and Figures S14 and S15) depending on the ligand, confirming the aggregation tendency of these cyanines. In contrast, **C5-Sulf** cannot form aggregates in these conditions, probably because of the negatively charged sulfonate groups of the ligand.

### Fluorescence Resonance Energy Transfer (FRET) Melting Assays

Once the aggregation properties were studied, we conducted FRET melting assays to assess the interaction of the ligands toward a panel of G-quadruplex and duplex DNAs (see Table S2 for sequences). A FRET assay is based on doubly labeled oligonucleotide sequences which fold into a secondary DNA structure, either a G-quadruplex or a duplex. When folding, both dyes are in close proximity to each other, and the fluorescence emission is quenched by a FRET process, but upon heating, both dyes pull away, and the fluorescence is recovered. The FRET melting curves provide a melting point (*T*_m_) denoting half of the structure unfolded, which is characteristic of each structure and sequence. Upon interacting with a ligand, the melting point increases, yielding Δ*T*_m_ values as a quantitative measurement of the interaction. We selected G-quadruplexes of different topologies, including antiparallel (*22CTA*, *K-ras*, *TBA*, and *G*_*4*_ *C*_*2*_), parallel (*cMyc*, *Kit*_*1*_, *Kit*_*2*_, *CEB25*), and mixed/hybrid (*hTelo*), to study the influence of the topology of the G-quadruplex in the interaction. Among the ligands containing identical indolizine moieties and polymethine chains of different lengths, **C1**–**C3**, the Δ*T*_m_ values (Figure 3) are higher for longer chains (**C1** < **C2** < **C3**), indicating that the higher structural flexibility conferred by a 3 or 5 carbon polymethine chain is needed to interact with G4s. On the other hand, considering the cyanine ligands with the longer polymethine moiety, the substitution of the indolizine groups with an electron-donating group (methoxy, **C4**) and an electron-withdrawing group (cyano, **C5**) enhances the stabilization of all G4 topologies compared to their unsubstituted **C3** analog. However, substitution of the same indolizine scaffold with a sulfonate group (**C5-Sulf**) markedly diminishes the stabilization effect for all G4s, indicating that negatively charged groups decrease the interaction with G4 DNA.^63–65^ To complete our G4-ligand interaction studies, we conducted FRET melting experiments using a G4 RNA derived from the oligonucleotide sequence GGGGCCGGGGCCGGGCCGGGGCC (*r*(*G_4_C_2_*)) folding into an antiparallel RNA G-quadruplex structure. The cyanine ligands **C2** and **C3** slightly stabilized this RNA at a high molar ratio (≈7–9 °C, see Figure S16), suggesting that these ligands have a high interaction toward G4 DNA over G4 RNA. Interestingly, all the ligands show a very low stabilization of the duplex model (*ds26*), denoting a selectivity for G4 over the duplex structure.

**Figure 3:**
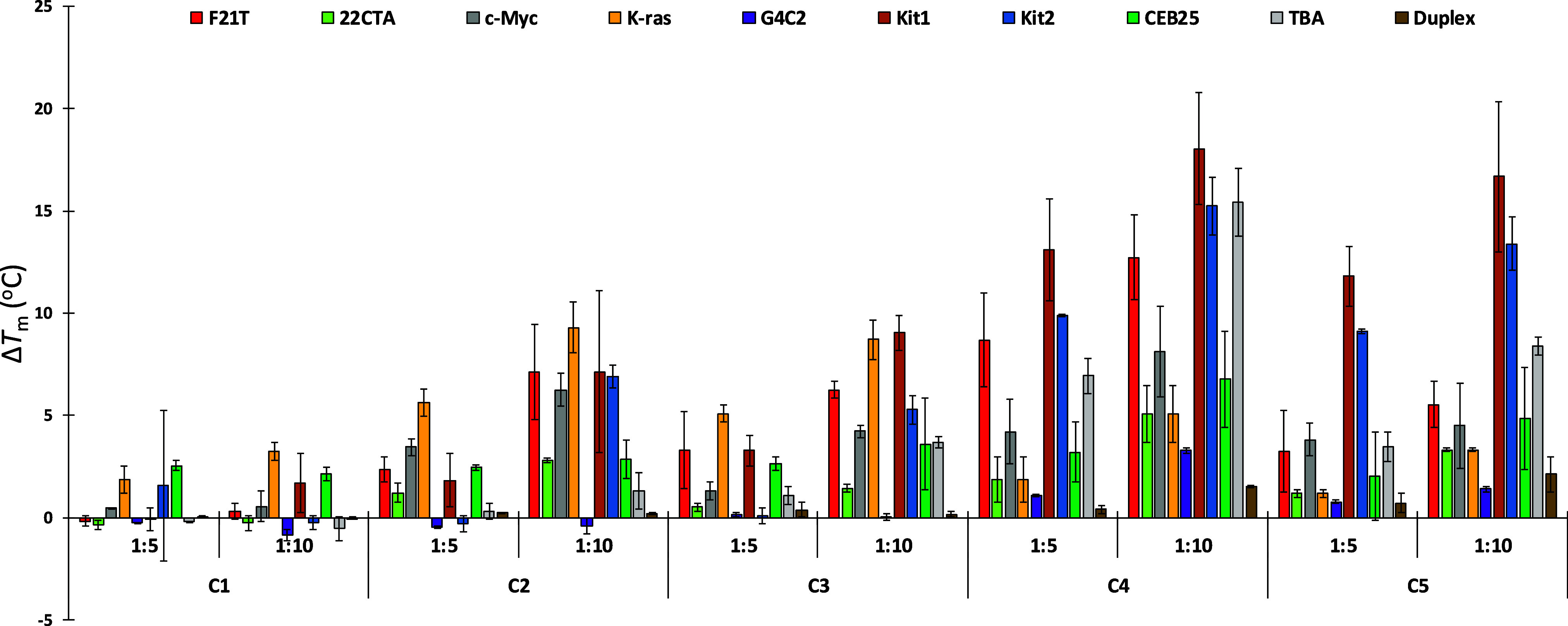
Plot of Δ*T*_m_ (°C) for the interaction of cyanine-based ligands with various G4s and duplex DNA (*ds26*).

To evaluate thoroughly the G4 versus duplex selectivity, competition FRET melting assays were carried out with **C2** and **C3**, because these cyanine ligands induce remarkable G4 DNA stabilization. The addition of an excess of duplex DNA (up to 100 equiv) did not yield a significant decrease in the values of Δ*T*_m_ for *hTelo* and *cMyc* (Figure S17). It highlights the selectivity of both ligands for G4 over duplex sequences, which corroborates the results discussed previously.

### UV–Vis Experiments

Following the FRET melting evaluation, we assessed the binding of the ligands to the DNA structures by UV–vis titrations. We investigated G4 DNAs of different topologies, covering the mixed/hybrid (*hTelo*), hybrid-1 (*24TTG*), hybrid-2 (*26TTA*), and parallel (*cMyc* and *Kit*_*1*_) topologies and the double-stranded DNA (*ds26*). UV–vis titrations of **C1** with DNAs showed no change in the UV–vis spectra, confirming the low affinity of this cyanine for DNAs previously obtained by FRET melting experiments.

The vis spectra of **C2** containing the three-carbon linker exhibited a larger band centered at 620 nm, together with a smaller band at 690 nm in buffered solution. Upon the addition of DNAs, a decrease in both bands with the appearance of a new band at 720 nm occurred for G4s and duplexes (Figures 4 and S18–S21 in ESI). The appearance of the new band was much smaller for the duplex DNAs.

**Figure 4:**
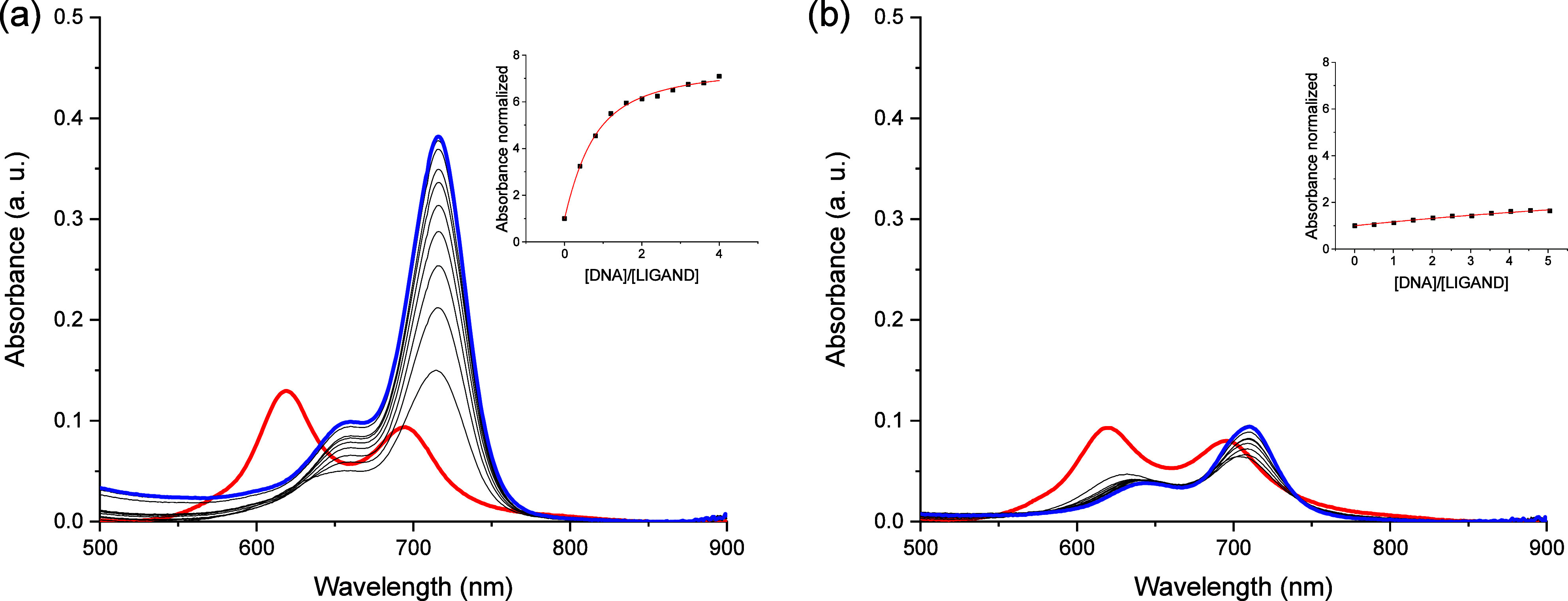
UV–vis titrations of **C2** with (a) *hTelo* and (b) *ds26* in *Tris* 10 mM, KCl 100 mM, and pH 7.4. The red line corresponds to the initial spectrum and the blue spectrum to the last one of the titration. Inset: fitting of the absorbance versus ratio ([DNA]/[ligand].)

Cyanine **C3**, containing a longer polymethine linker than **C2**, had a broad band at 630 nm in *Tris* buffer. The Vis spectra of **C3** showed a decrease in intensity upon DNA titration while new bands appeared, the number and wavelengths of the bands depending on the DNA structure (Figures S22–S25). For G4s and duplexes, a band centered at 825 nm increased in intensity upon DNA addition, but in some cases, a smaller band at 750 nm appeared too. Only for *Kit*_1_ G4, the intensity of the lower wavelength band was stronger than the band centered at higher wavelengths. These bands can be attributed to different aggregation forms upon the DNA binding.

**C5** showed two visible bands centered at 590 and 680 nm in *Tris* buffer, assigned to the aggregate state of the ligand. The UV–vis spectrum of **C5** experienced a decrease of these bands and the appearance of a new band around 850 nm upon addition of DNA (either G4s or duplex) (Figures S26–28). This new band can be assigned to the monomer, which hinders the molecular rotation of the ligands, resulting in a fluorescence enhancement. Among G4 structures, a larger effect is observed for the parallel G4 structures *Kit*_*1*_ and *cMyc*, indicating the major monomer disassembly ability for the parallel G4s of **C5**. Strikingly, the duplex showed a different behavior, in which the titration of **C5** with *ds26* showed a larger increase in the new band at a longer wavelength (Figure S22) than the G4 structures. We performed Job’s plot studies by UV–vis, indicating that two molecules of cyanines were bound per G4 for **C2**, **C3**, and **C5**, while only one cyanine **C5-Sulf** was bound to a *cMyc* G4 DNA (Figures S29–S32).

The apparent affinity constants were obtained from the UV–vis titrations to afford the values gathered in Table 1. The affinity values for the five-carbon linker cyanines (**C3**–**C5**) range between 10^3^ and 10^5^ M^–1^, varying with the DNA structure but showing similar values for duplexes and G-quadruplexes, suggesting a poor selectivity. With regard to **C2**, the values are higher for G4s than duplexes for the three duplex DNA structures (see Figures 4 and S21), indicating the highest selectivity against the tetrameric DNA structures from the cyanine ligands.

**Table 1:** Caption Values of *K*_a_ Obtained from the Fitting of the UV–Vis Titration Experiments^c^

	C2	C3	C5
*hTelo*	1.1 × 10^5^ ^a^ 5.6 × 10^5^ ^b^	1.7 × 10^4^	1.1 × 10^4^
*cMyc*	8.0 × 10^5^ ^a^ 4.7 × 10^5^ ^b^	2.2 × 10^4^	1.9 × 10^4^
*ds26*	3.1 × 10^3^ ^a^ 4.6 × 10^4^ ^b^	1.6 × 10^4^	6.9 × 10^3^
*24TTG*	1.5 × 10^5^ ^a^	1.1 × 10^4^	1.4 × 10^4^
*26TTA*	2.3 × 10^5^ ^a^	1.3 × 10^4^	2.2 × 10^4^
*Kit*_*1*_	4.3 × 10^5^ ^a^	2.6 × 10^4^	1.0 × 10^4^

a Obtained by UV–vis experiments.

b Obtained by fluorescence titration.

c Performed in *Tris* buffer 10 mM, KCl 100 mM, and pH 7.4.

### Fluorescence Emission Assays

Then, we explored the potential light-up effect of the ligands upon addition of DNA. These cyanine ligands are initially aggregated in aqueous solution, and they are poorly emissive except for **C2**, which has a low fluorescence emission band at 730 nm. The interaction of cyanine **C2** with DNA yielded the aggregation disassembly, forming monomer species, which restricts the molecular motion and enhances the fluorescence emission (Figure 5). We performed fluorescence titrations with G4s and duplex DNA, and all the DNA showed a light-up effect, showing a larger fluorescence emission increase for the G-quadruplexes (Figures 5 and S33–S36). The fluorescence titrations were analyzed, and the binding constants were obtained and are collected in Table 1. We obtained values in agreement with the UV–vis experiments, denoting the preference of cyanine **C2** for G4s.

**Figure 5:**
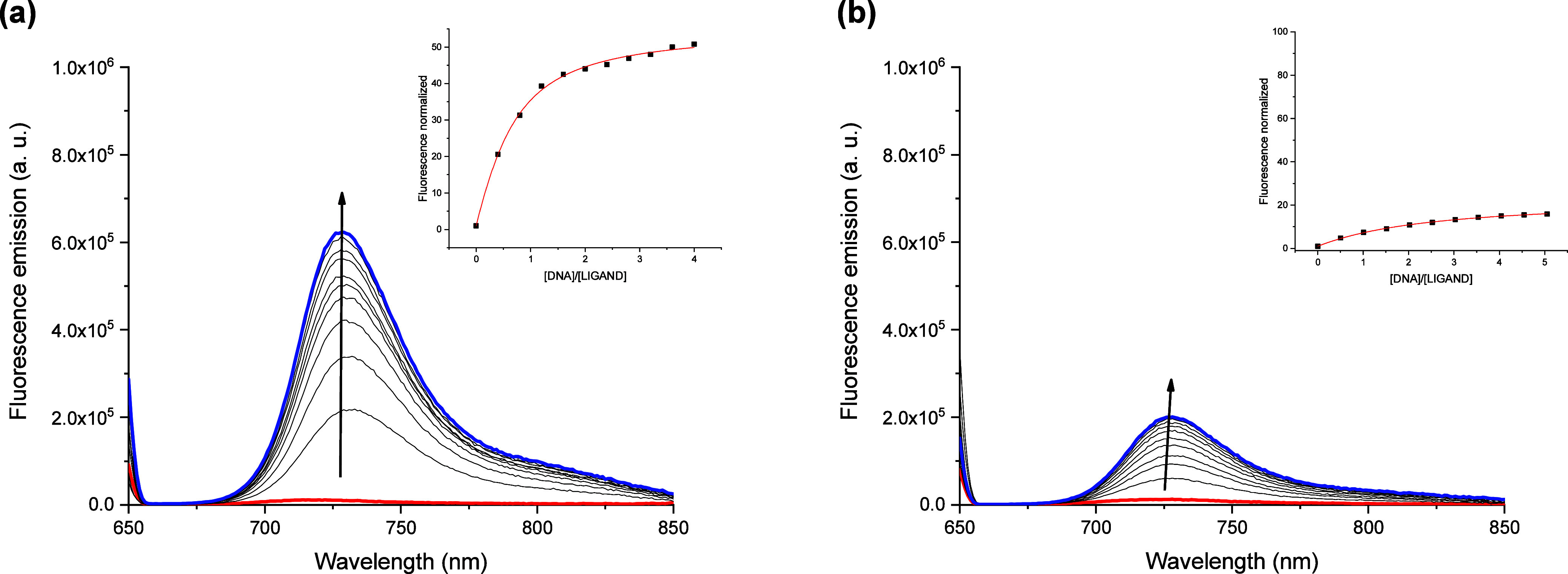
Fluorescence emission titrations of **C2** with (a) *hTelo* and (b) *ds26* in *Tris* 10 mM, KCl 100 mM, and pH 7.4. The red line corresponds to the initial spectrum and the blue spectrum to the last one of the titration. Inset: fitting of the fluorescence normalized versus ratio ([DNA]/[ligand]).

### Theoretical Calculations

We then carried out computational studies at the B3-LYP/def2-TZVP level of theory (see methods in ESI for more details on the calculation protocols) to assess (*i*) the energetic balance between the *cis* and *trans* configurations in *bis*-indolizines **C1** to **C5,** as well as (*ii*) their binding affinities toward a G-quartet. First, we explored the *trans*–*cis* isomerization of the cyanines in solution, which can impact the binding toward the G-quartet of a G4. In Figure 6a, the relative energy differences (Δ*E*, in kcal·mol^–1^) between the *cis* and *trans* configurations in water are shown for **C1** to **C5** compounds. As noted, in both **C1** and **C2**, the *trans* configuration was more stable (negative Δ*E* values), while in the case of compounds **C3** to **C5**, the opposite was observed, resulting in a major stabilization of the *cis* configuration (positive Δ*E* values). Due to the small energetic barriers found between both configurations (except for **C2**), we decided to carry on with both isomers to the next phase of the computational study.

**Figure 6:**
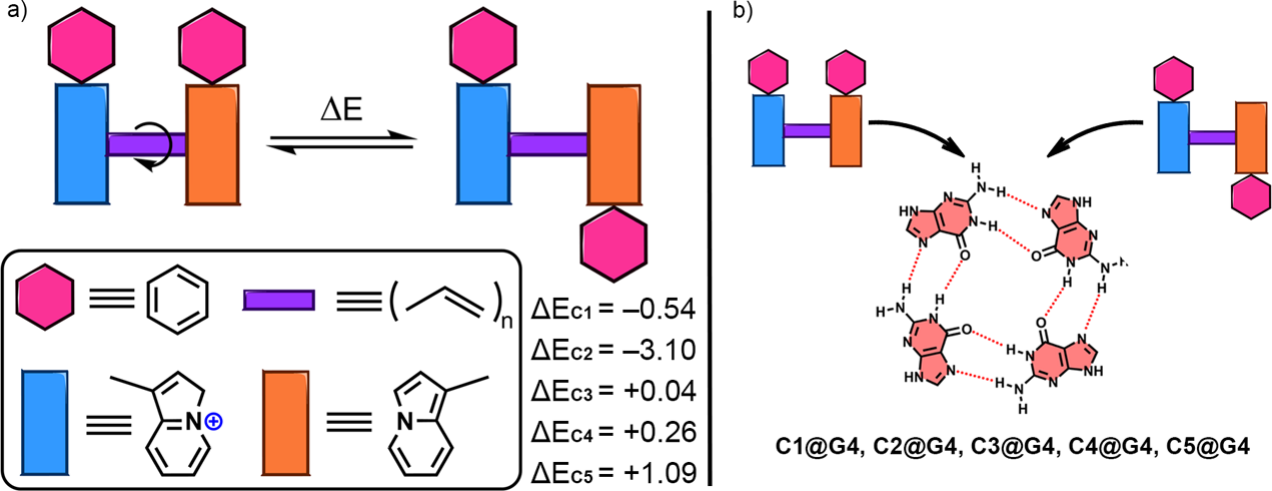
(a) Energetic difference between the *cis* and *trans* configurations of the *bis*-indolizines **C1** to **C5** in water. Energy values (Δ*E*) are given in kcal·mol^–1^. Negative Δ*E* values refer to a more stable *trans* configuration, while positive Δ*E* values refer to a more stable *cis* configuration. (b) Schematic representation of the complexes studied involving both *cis* and *trans bis*-indolizines and a G-quartet.

To understand the binding affinities of these *bis*-indolizines toward G4s, we used compounds **C1** and **C5** and a G-quartet as theoretical models (see Figure 6b). In Figure 7, the Molecular Electrostatic Potential (MEP) surfaces (0.001 au) of compounds **C1** to **C3** in both *cis* and *trans* configurations are shown. In all cases, very positive values were obtained due to the cationic nature of the *bis*-indolizine moieties. The MEP minima corresponded to the phenyl groups (red-colored regions in Figure 7), while the MEP maxima were located near the cationic N atoms belonging to the indolizine ring (blue-colored regions in Figure 7). We observed a progressive decrease (less positive) in the MEP values over the indolizine rings, ongoing from **C1** to **C3** in both *cis* and *trans* configurations, which points out an increment of the charge delocalization upon extending the length of the cyanine bridge, as expected. For compounds **C4** and **C5** (see Table 2), the MEP values showed the expected trend compared to compound **C3**, that is, compound **C4** (involving a methoxy group as an aromatic substituent) achieved lower MEP values over the indolizine ring (+57.7 and +58.4 kcal·mol^–1^ for both *cis* and *trans* configurations, respectively), while in the case of compound **C5** (involving a cyano group as an aromatic substituent), the contrary was observed (+66.5 and +65.8 kcal·mol^–1^ for both *cis* and *trans* configurations, respectively), in line with the electron donor and electron-withdrawing nature of these substituents. From the inspection of the electrostatic potential surfaces, those complexes involving **C1** should be more favorable in terms of stability than those involving compounds **C2** to **C5**. Among the rest, those that encompass compounds **C2** and **C5** should be more favored than the ones implying compounds **C3** and **C4**. However, this analysis only accounts for electrostatics and other factors (e.g., size and shape complementarity) that might also be important for the binding affinity between the *bis*-indolizines and the G4-quartet moieties.

**Figure 7:**
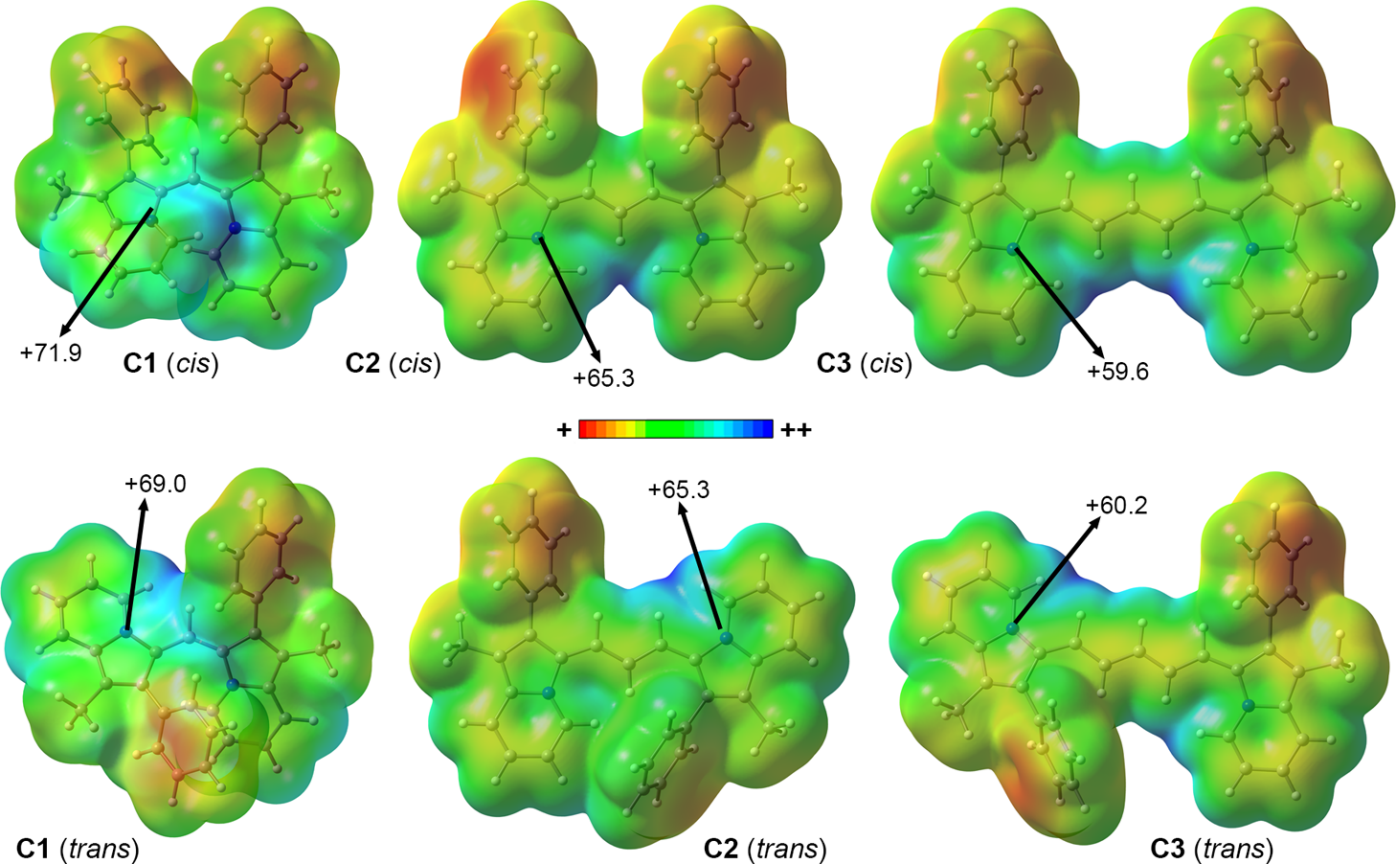
Molecular Electrostatic Potential (MEP) surfaces of **C1**, **C2**, and **C3**. Energy values at concrete points of the surface are given in kcal·mol^–1^ at 0.001 au. The values corresponding to all compounds used are gathered in Table 2.

**Table 2:** Values of the Electrostatic Potential at the N Atom from the *bis*-Indolizine Moiety (V_N_, in kcal·Mol^–1^) at 0.001 a.u

Ligand	V_N_ (*cis* configuration)	V_N_ (*trans* configuration)
**C1**	+71.9	+69.0
**C2**	+65.3	+65.3
**C3**	+59.6	+60.2
**C4**	+57.7	+58.4
**C5**	+66.5	+65.8

In Figure 8, the NCIplot analyses of several optimized (B3-LYP/def2-TZVP level of theory) **Cn@G4** complexes are shown (see Figure S37 in ESI for those corresponding to **C4** and **C5** involving complexes), while the interaction energy values in water are gathered in Table 3. As noted, in all the cases, attractive and moderately strong interaction energy values were obtained (ranging between −24.6 and −15.0 kcal·mol^–1^). In general, those complexes involving **C3** to **C5** achieved larger interaction energy values than those involving **C1** while having a lower (in the case of the *cis* configuration) or a similar/higher (in the case of the *trans* configuration) stability compared to complexes involving **C2**. Another interesting aspect to note is the increase in the binding affinity upon increasing the length of the cyanine linker (from **C1** to **C2**), although the opposite was observed from **C2** to **C3** involving complexes.

**Figure 8:**
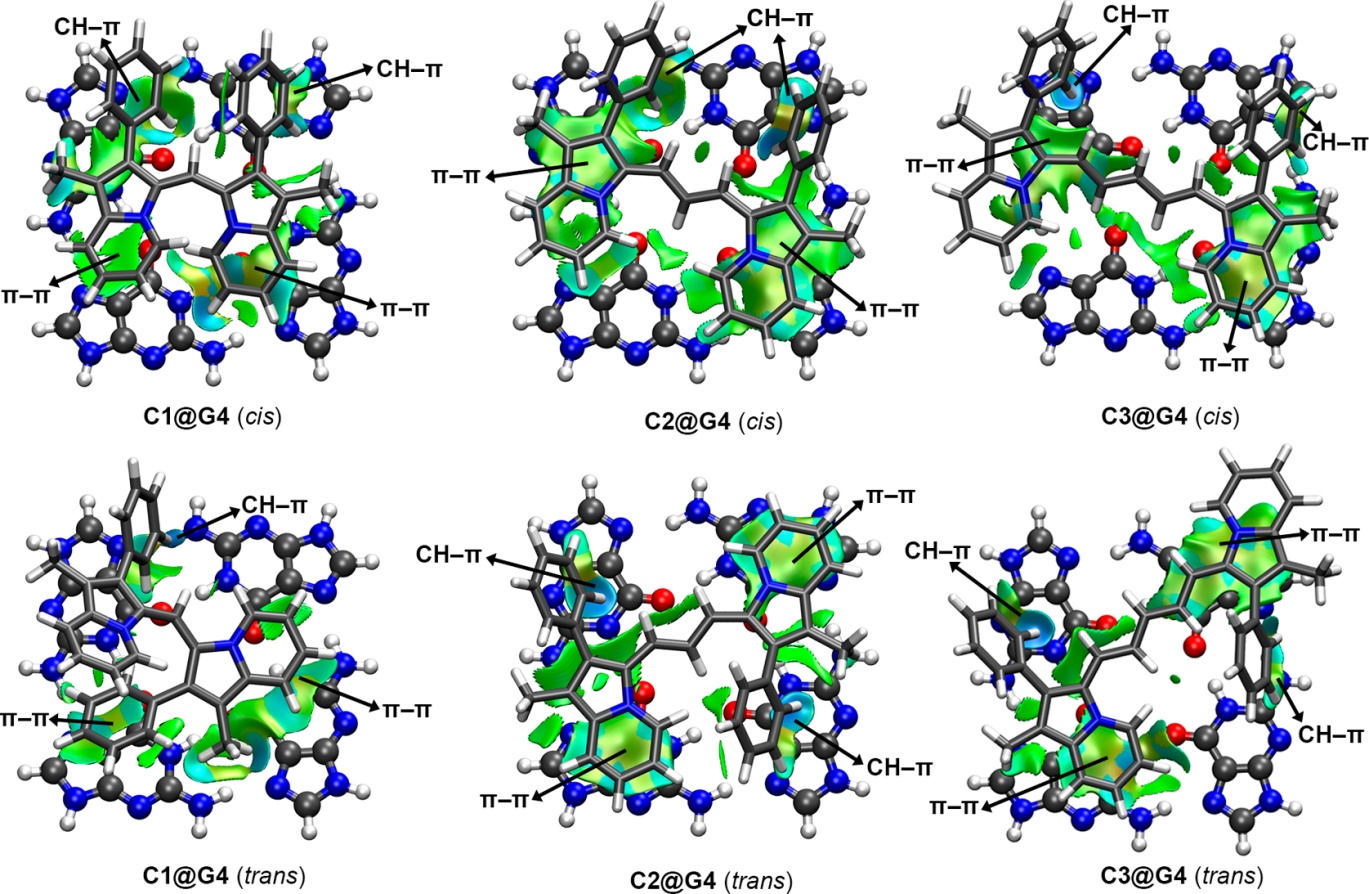
NCIplot surfaces of **C1** to **C3** in both *cis* and *trans* configurations complexed to the G4-quartet. The different types of noncovalent interactions present in each complex are also indicated. The density and RDG cutoff values = 0.5 and 1.0, respectively. The density and RDG cutplot values are 0.07 and 0.3 au, respectively. Surfaces created using the fine multigrid option.

**Table 3:** Values of the Interaction Energies (Δ*E*, in kcal·mol^–1^) for Complexes Involving Compounds **C1** to **C5** and a G4-Quartet in Water and Their Equilibrium Distances (d, in Å)

	*cis* configuration		*trans* configuration	
Ligand	Δ*E*	d^a^	Δ*E*	d^a^
**C1@G4**	–15.0	3.925	–13.1	4.808
**C2@G4**	–22.3	3.078	–20.5	3.362
**C3@G4**	–19.7	2.903	–20.2	3.551
**C4@G4**	–18.5	3.463	–21.0	3.782
**C5@G4**	–19.2	3.470	–24.6	5.070

a Values are given as the distance between the central C atom from the cyanine bridge to the G4 quartet plane.

To rationalize these results, which are contrary to the MEP analysis discussed above, we computed the NCIplot analyses (see Figure 8), and it can be noted that there is a more extended interacting region (larger isosurface) between the *bis*-indolizine and the G4-quartet for those complexes involving **C2** and **C3** compared to **C1**-involving complexes. In fact, both *cis* and *trans* configurations of **C2@G4** are those that showed the best size complementarity between both counterparts, resulting in larger interaction energy values compared with **C1** and **C3** involving complexes. These results agree with those retrieved from FRET melting assays, which point out that **C1** cannot interact with G-quadruplexes. Moreover, it is also in concordance with competition FRET melting experiments and UV–vis titrations, highlighting that **C2** is a more selective binder for G4 over duplex than the rest of the cyanine ligands of the series.

These computations were useful to elucidate the noncovalent interactions (NCIs) involved in the **Cn@G4** quartet recognition. In more detail, π–π interactions were undertaken between the indolizine rings and the π-system of the G4-quartet, which accounted for the large and greenish isosurfaces shown in Figure 8. On the other hand, CH–π interactions are involved in the stabilization of the phenyl moieties (interacting with either the five- or six-membered ring of guanine). The NCIplot analysis is also an interesting tool to assess the relative strength of the NCIs present in those systems from a qualitative point of view. In this regard, we observed the presence of strong CH-π bonds in complexes involving **C3** (*cis* and *trans*) and **C2** (*trans*) due to the bluish color, which denotes the CH–π interacting region compared to the greenish isosurface found in the case of the π–π stacking interactions. In the rest of the complexes, a similar color on the isosurface that characterized both interactions was observed, thus indicating a similar contribution to the total binding affinities.

### Molecular Docking of Duplex DNA-Cyanine Interaction

We last explored the binding mode of the cyanine ligands to duplex DNA by molecular docking. The minimum energy conformers showed that the ligands bind into the minor groove of the duplex DNA, except **C1**, which binds into the major groove (see Figures 9 and S38–S43). The ligands twisted into the minor groove, adapting to the groove dimensions and locating the phenyl groups in the groove while the other phenyl unit points outside the helix. The main driving noncovalent forces of the interaction are hydrophobic and electrostatic interactions (Figures S44–S49). The predicted binding energies range from Δ*E* −7.0 to −10.2 kcal·mol^–1^ (Table S13), decreasing as the length of the polymethine linker increases.

**Figure 9:**
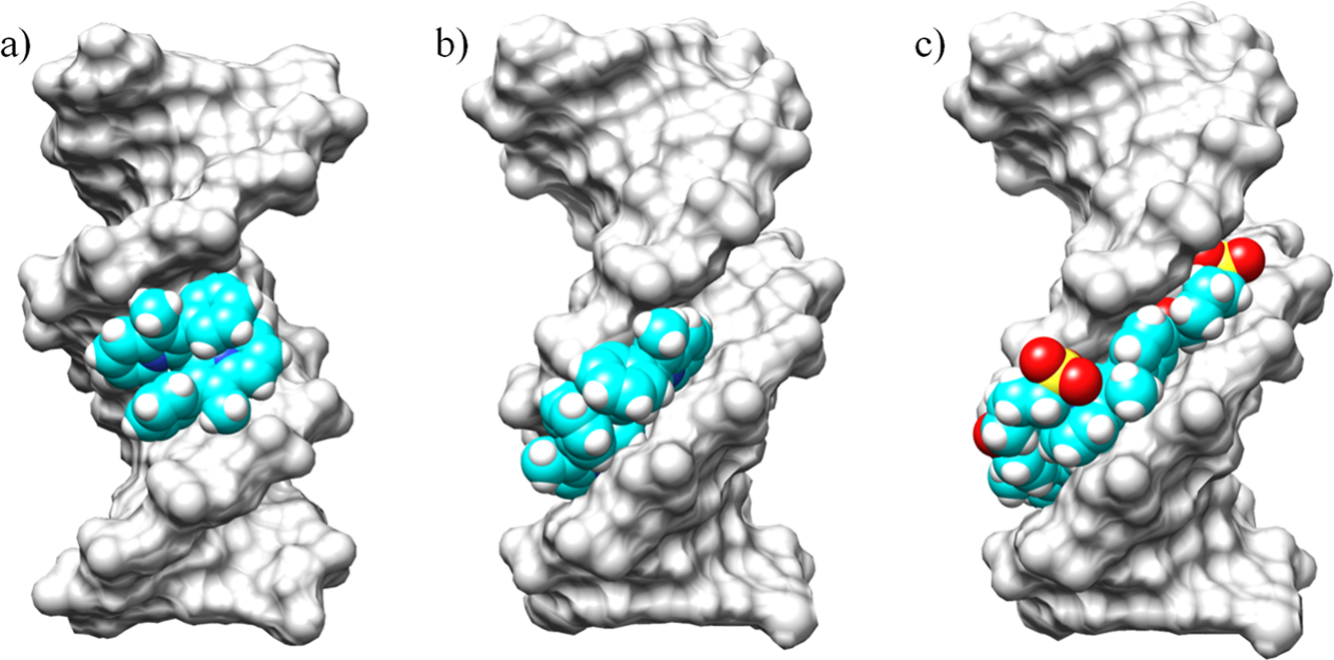
Representative view of duplex DNA (PDB: 296D) binding with cyanines (a) **C1**, (b) **C3**, and (c) **C5-Sulf**.

### Assessment of the Cell Viability

Once determined the biophysical properties and interaction with DNA were determined, we conducted cellular viability assays using an ATP-based luminescence assay in the cancer cell lines A549, MCF7, and HeLa (lung, breast, and cervical cancer cells, respectively) and macrophage cells (Raw 264.7). All the ligands except **C5** have IC_50_ values ranging between 1 and 10 μM (see Table 4 and Figure S50), denoting the high antiproliferative activity of cyanine-like ligands, in particular **C3**, which shows values lower than 0.5 μM for all cell lines.

**Table 4:** IC_50_ Mean ± SD Values for the Indicated Cell Lines, Obtained from Two Independent Experiments, Each Conducted in Triplicate

	IC_50_ (μM)			
ligand	A549	MCF7	Raw 264.7	HeLa
**C1**	1.41 ± 0.25	3.86 ± 0.10	1.44 ± 0.13	2.94 ± 0.09
**C2**	0.29 ± 0.03	0.92 ± 0.04	0.38 ± 0.01	0.19 ± 0.06
**C3**	0.34 ± 0.08	0.44 ± 0.08	0.52 ± 0.04	0.43 ± 0.02
**C4**	0.52 ± 0.14	0.88 ± 0.12	1.00 ± 0.02	0.69 ± 0.15
**C5**	21.08 ± 0.91	10.08 ± 2.25	19.43 ± 4.11	8.60 ± 0.05

### Transcriptome Profiling of C3-Treated Cells

According to the overall studies, **C3** is a potent G4 binder as shown by FRET melting, photophysical, and computational studies, but it also binds to duplexes, resulting in moderate selectivity for G4s. Because of these outcomes and the broad application of cyanine containing a five-carbon-length linker as fluorescent tags in biomolecules (oligonucleotides and proteins), we selected **C3** for conducting a transcriptome analysis. These analyses can assist us to understand the mechanism of action and unravel whether **C3** is acting via a G4-targeting mechanism in HeLa cancer cells.^18,66,67^ Using the cell viability assays, the HeLa cell line was treated for 24 h with 40 nM **C3** (calculated as 1/10 of the IC_50_ at 24 h), RNA was harvested, and gene expression levels were analyzed by RNA-seq *DESeq2* software was used to perform a comprehensive analysis of the significant transcriptional response to **C3** treatment, which identified 138 significantly deregulated genes (adjusted *p* < 0.05). These genes comprise 76 upregulated and 62 downregulated transcripts (Figure 10A; Supporting Information). The most highly expressed genes, *NIBAN1*, *TRIB3*, *GDF15*, *DDIT3*, and *SESN2*, as well as several aminoacyl-tRNA synthetases, were associated with stress adaptation and amino acid metabolism. In contrast, *CCL2*, *TTYH3*, and *DHCR24*, which are involved in lipid metabolism and inflammatory signaling, were downregulated. Consistently, hierarchical clustering of these genes (Figure 10B) clearly separated the control and treated samples. Despite the limited number of replicates (two controls and three treated samples), intragroup clustering was well preserved, confirming the reproducibility of the transcriptional responses. This highlights the coordinated activation of biosynthetic and proteostatic programs in the **C3**-treated cells.

**Figure 10:**
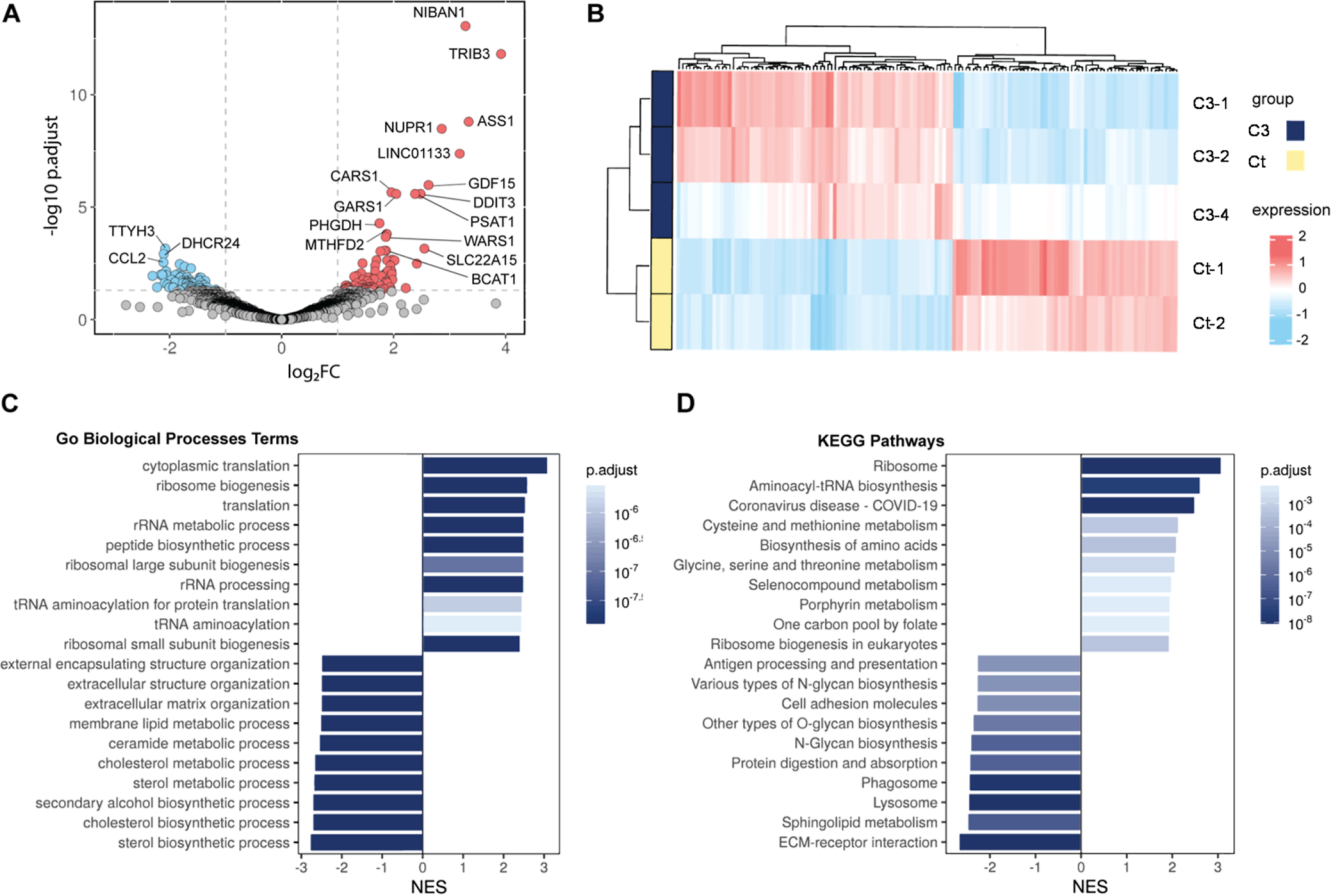
Volcano plot (A) and heatmap (B) of differentially expressed genes. Gene ontology (C) and KEGG pathways (D) analyses of the metabolic and cellular pathways.

Functional enrichment analysis (Figure 10C,D) provided a consistent view of the transcriptional reprogramming induced by **C3**. GO Biological Process analysis identified 1.042 significant terms, revealing activation of translation, ribosome biogenesis, and amino acid biosynthesis, together with repression of extracellular matrix organization and sterol and lipid metabolic processes. To minimize redundancy, the enriched GO terms were clustered by semantic similarity using the *Revigo* R package, grouping them into 25 positively and 91 negatively enriched clusters. Among the ten clusters with the highest and lowest scores, processes such as ribosome biogenesis, oxidative phosphorylation, and protein polyubiquitination were among the most positively enriched, whereas isoprenoid biosynthesis, basement membrane organization, and cell adhesion dominated the negatively enriched clusters, confirming the functional coherence of the enrichment results (Supporting Information). It shall be noted that previous works have shown that G4 ligands target the rRNA pathway, as shown by **C3**.^17–19^

Consistently, KEGG pathway analysis revealed 214 significantly enriched pathways, reinforcing the GO-derived trends. Positively enriched pathways (Figure 10D) included ribosome, aminoacyl-tRNA biosynthesis, and carbon metabolism, while ECM-receptor interaction, sphingolipid and sterol metabolism, and lysosomal pathways were downregulated. Together, these results depict a coordinated metabolic and proteostatic shift, where **C3** treatment promotes biosynthetic and stress-adaptive activity while suppressing lipid metabolism, ECM remodeling, and vesicular transport, indicative of a transition toward a less-proliferative, stress-responsive phenotype. All of the deregulated pathways associated with RNA biosynthesis and ribosome modulation suggest that **C3** can act through targeting G4s at the RNA level.

## Conclusion

Cyanines are largely used in biochemical and biophysical assays for the detection and quantification of nucleic acids and proteins, although the DNA sequence and structure preferences are underexplored in the literature. Herein, we developed a series of six cyanine ligands (**C1**–**C5-Sulf**) differing in the polymethine length and the heterocyclic moieties and investigated their interaction with G-quadruplex DNA structures. The ligands with three- and five-carbon polymethine linkers experienced an aggregated form in aqueous solution, which broke down in MeOH solution or upon DNA binding as demonstrated by UV–vis and fluorescence spectroscopies.

Strikingly, cyanine **C2**, containing a three-carbon linker, bound more strongly to G-quadruplex structures than duplex DNA due to adequate ligand flexibility, which enables effective interaction with the G-quartet unit while reducing its ability to adopt a propeller conformation required for duplex binding. Cyanine **C1** with only a one-carbon-length connector is unable to interact with both G-quadruplex and duplex DNAs, as demonstrated by FRET melting and UV–vis experiments. The cyanines containing a five-carbon polymethine linker showed similar binding affinities for duplex and G4 structures according to UV–vis results, along with a strong stabilization effect for G4 and low stabilization for duplex DNA. This indicates that the longer connector facilitates these cyanines to adopt the needed conformation to bind to both tetrameric and dimeric DNA structures.

On the other hand, theoretical calculations at the B3-LYP/def2-TZVP level of theory unraveled the noncovalent nature and extension in real space of the CH–π and π–π interactions involved in the cyanine···G4 quartet recognition phenomena. In general, results from calculations agree with the experimental findings, since complex **C1@G4** obtained the poorest interaction energy value, while complex **C2@G4** achieved the largest interaction energy value for the *cis* conformation. Furthermore, among the **C3** and **C4** compounds, both obtained a similar energy interaction strength upon interacting with the G4, also in agreement with the results obtained from experiments.

Lastly, whole-transcriptome RNA-seq analysis showed global gene expression in HeLa cells treated with **C3**. It revealed the up- and downregulation of a large number of genes in essential pathways of RNA and metabolism. The changes produced by **C3** represent a global response to a complex mechanism, involving mostly RNA processing, which sheds light on the cellular activity of cyanines and their potential to exhibit a G4-targeting mechanism.

The insights of this work provide an interesting view when using cyanine ligands for detecting and binding DNA structures depending on the nucleic acid conformation. Moreover, it covers the essential structural features for designing G4 probes and ligands based on the architecture of cyanine molecules.

## Supplementary Material





## Notes

Tables of sequences, hydrodynamic size, Cartesian coordinates of complexes, and values of binding affinity of complexes and figures of DLS, UV–vis, and fluorescence titrations, molecular docking of complexes, and NCIplot surfaces of complexes (PDF)
RNA-seq analysis data (CSV)
