# Supplementary material for: Unveiling the Structural Modifications of Cyanines to Target G‑Quadruplex DNA through Biophysical, Computational, and Transcriptome Analyses
Source: ACS Omega. 2026 May 15;11(21):30537–49. doi: 10.1021/acsomega.5c11600 (PMC13234662; doi:10.1021/acsomega.5c11600)
Supplement: Supplementary file 1 [file ao5c11600_si_001.pdf]

Unveiling the structural modifications of cyanines to targeting G-quadruplex DNA through biophysical, computational and transcriptome analysis

Cristina Galiana-Roselló[1,2][#], Andrea Lázaro-Gómez[1#], Ariadna Gil-Martínez[1], Anargyros Drolapas[1], William E. Meador[3], Catalina Nicolau[4], Chun-Qiong Zhou[5]*, Jared H. Delcamp[3]*, Antonio Bauza[4]*, Jorge González-García[1]**

[1] University of Valencia, Department of Inorganic Chemistry, Institute of Molecular Science (ICMol), Catedrático José Beltrán 2, 46980 Paterna, Spain

[2] Príncipe Felipe Research Center, Eduardo Primo Yúfera, 3, 46012 Valencia, Spain

[3] Air Force Research Laboratory, Materials and Manufacturing Directorate (RXNC), 2230 Tenth Street B655, Wright-Patterson AFB, OH 45433, USA

[4] Universitat de les Illes Balears, Department of Chemistry, Crta de Valldemossa km 7.5, 07122 Palma de Mallorca, Baleares, Spain

[5] Southern Medical University, School of Pharmaceutical Sciences, Guangzhou 510515, P. R. China

*Corresponding Authors: J.G.-G., A.B., C.G.-R., J.H.D. and C.-Q.Z. Email Address: jorge.gonzalez@uv.es, antonio.bauza@uib.es, cgaliana@cipf.es, jared.delcamp.1@us.af.mil, zcqlg@smu.edu.cn

[#]C.G.-R. and A.L.-G. contributed equally to this work

General Information

All reagents from commercial sources were used without further purification, unless otherwise noted. UV–Vis measurements were performed on a Cary UV-Vis 100 double-beam spectrophotometer and baseline corrected. Extinction molar coefficients were calculated by interpolation of data obtained from five solutions. Steady-state emission and excitation spectra were recorded on an FPI fluorescence spectrometer (Edinburgh Instruments) equipped with a single grating monochromator on both the excitation and the emission sides.

Table S1. Oligonucleotide sequences used in this work.

Name	Sequence (5' to 3')	Topology
**hTelo**	GGGTTAGGGTTAGGGTTAGGG<br>AGGGTTAGGGTTAGGGTTAGGG (for the labelled DNA)	Hybrid/mixed
**22CTA**	AGGGCTAGGGCTAGGGCTAGGG	Antiparallel
**cMyc**	TGAGGGTGGGTAGGGTGGGTAA	Parallel
**cKit1**	AGGGAGGGCGCTGGGAGGAGGG	Parallel
**cKit2**	CGGGCGGGCGCGAGGGAGGGG	Parallel
**CEB25**	AAGGGTGGGTGTAAGTGTGGGTGGGT	Parallel
**K-ras**	TCGGGTTGCGGGCGCAGGGCACGGGCG	Antiparallel
**TBA**	GGTTGGTGTGGTTGG	Antiparallel
**G4C2**	GGGGCCGGGGCCGGGGCCGGGGCC	Antiparallel
**ds26**	CAATCGGATCGAATTCGATCCGATTG	Duplex
**Duplex AT**	ATATATATATATATATATAT	Duplex AT-rich
**Duplex CG**	GCGCGCGCGCGCGCGCGCGC	Duplex GC-rich
**Z-G4**	TGGTGGTGGTGGTTGTGGTGGTGGTGTT	Left-handed G4
**i-Motif**	CCCTAACCCTAACCCTAACCCT	*i*-motif
**ssDNA**	TTTTTTTTTTTTTTTTTTTT	Single Stranded DNA
**r(G4C2)**	rGrGrGrGrCrCrGrGrGrGrCrCrGrGrGrGrCrCrGrGrGrGrCrC	Antiparallel G4 RNA

Table S2.- Average size of cyanine molecules in water.

Sample	Hydrodynamic Size (nm)
**C1**	361.8 ± 22.0
**C2**	355.6 ± 15.6
**C3**	83.4 ± 0.4
**C4**	240.1 ± 2.2
**C5**	94.6 ± 10.8

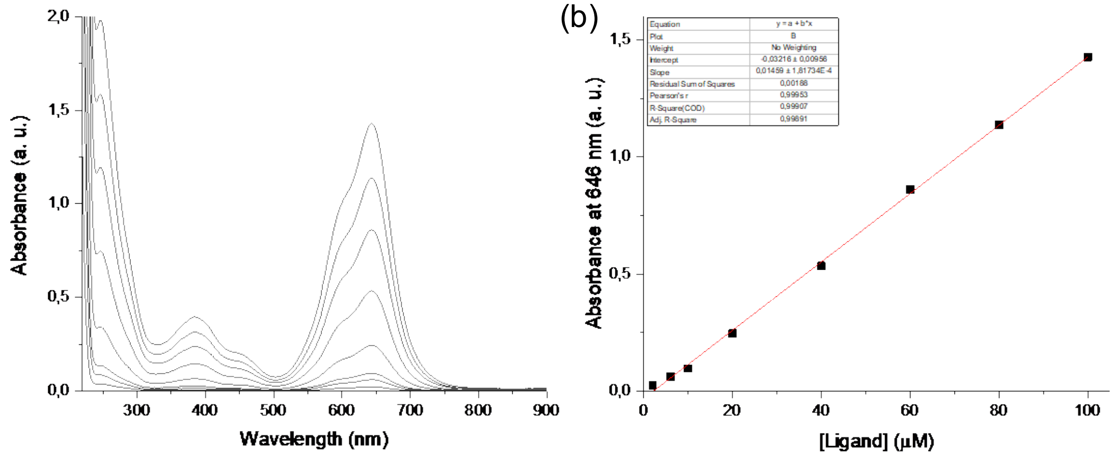

Figure S1.- (a) UV-Vis spectra of ligand **C1** at different concentrations. (b) Absorbance at the maximum versus the ligand concentration. Inset: Linear fitting of the experimental points.

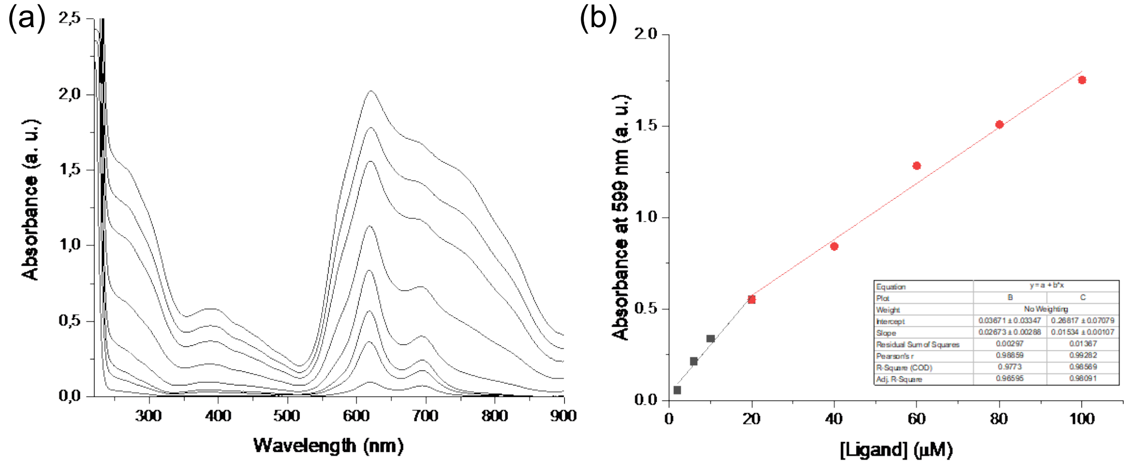

Figure S2.- (a) UV-Vis spectra of ligand **C2** at different concentrations. (b) Absorbance at the maximum versus the ligand concentration. Inset: Linear fitting of the experimental points.

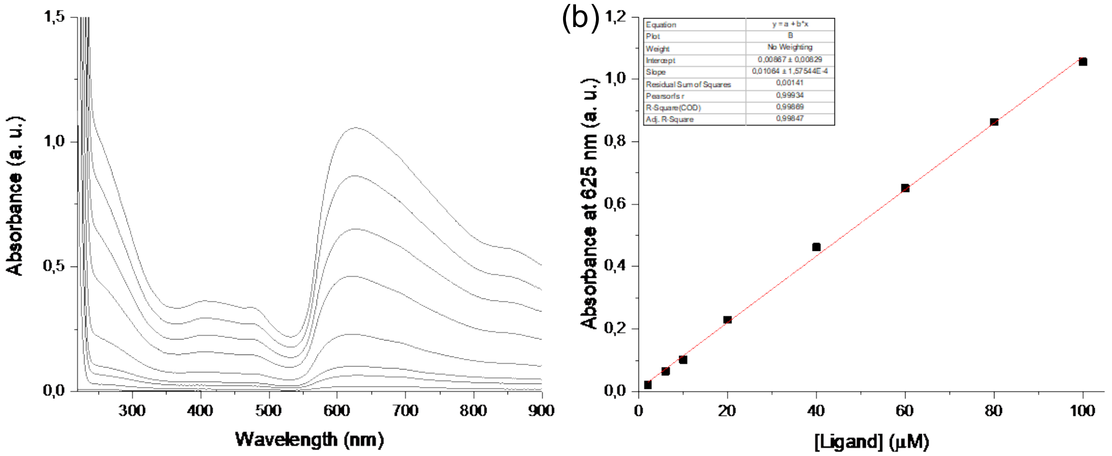

Figure S3.- (a) UV-Vis spectra of ligand **C3** at different concentrations. (b) Absorbance at the maximum versus the ligand concentration. Inset: Linear fitting of the experimental points.

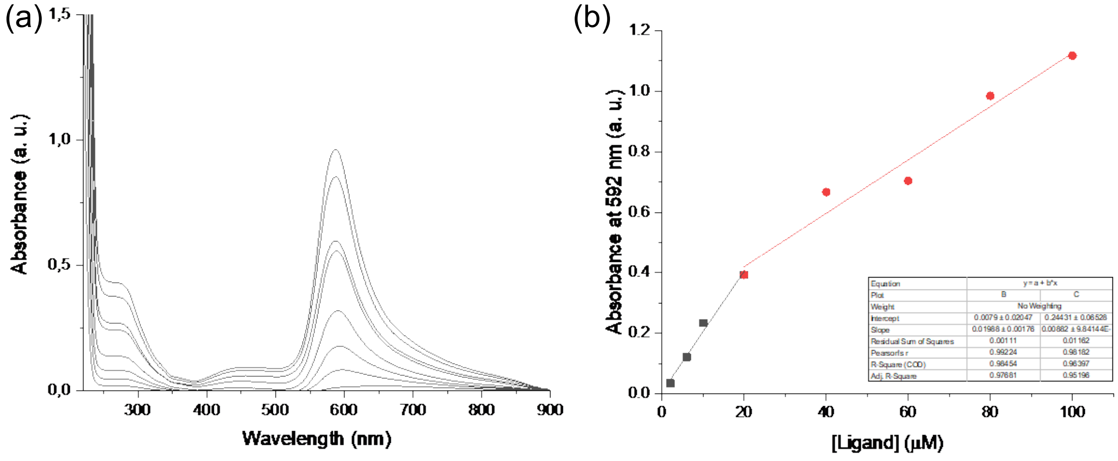

Figure S4.- (a) UV-Vis spectra of ligand **C4** at different concentrations. (b) Absorbance at the maximum versus the ligand concentration. Inset: Linear fitting of the experimental points.

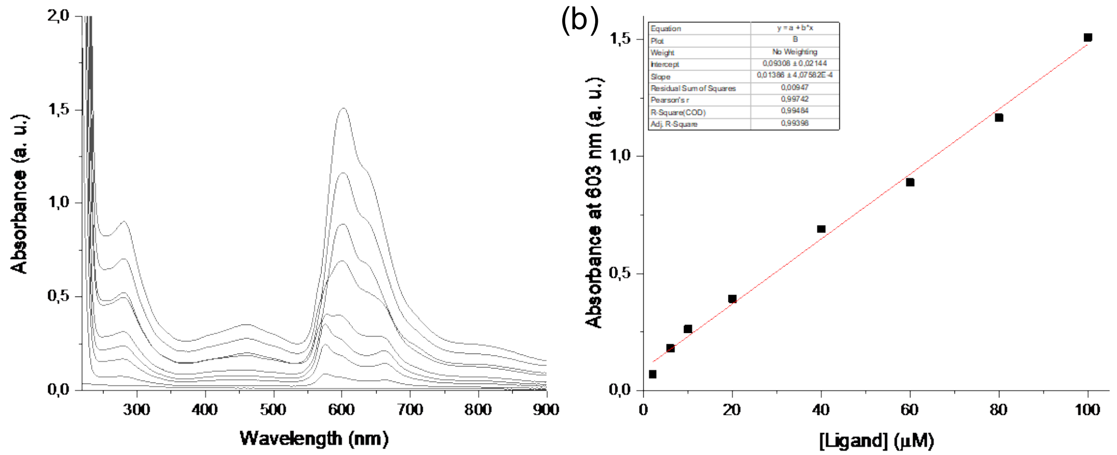

Figure S5.- (a) UV-Vis spectra of ligand **C5** at different concentrations. (b) Absorbance at the maximum versus the ligand concentration. Inset: Linear fitting of the experimental points.

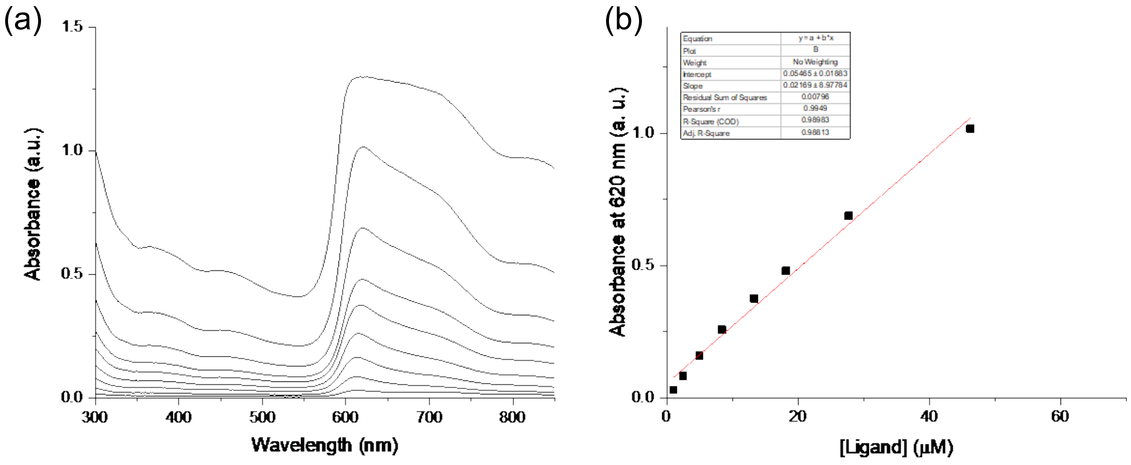

Figure S6.- (a) UV-Vis spectra of ligand **C5-Sulf** at different concentrations. (b) Absorbance at the maximum versus the ligand concentration. Inset: Linear fitting of the experimental points.

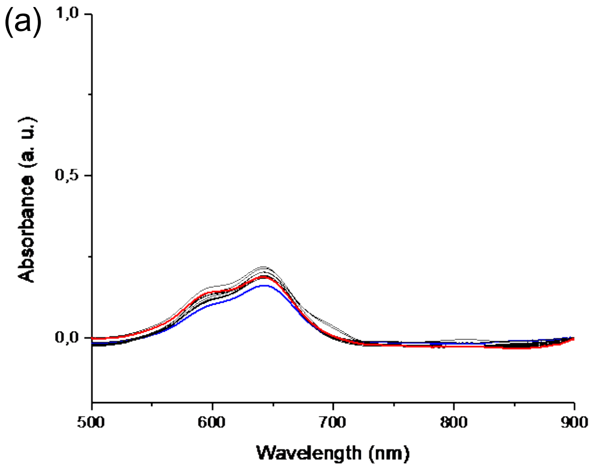

Figure S7.- (a) UV-Vis spectra of ligand **C1** in different solvent mixtures H_2O:MeOH (blue line 100 % H_2O, red line 100 % MeOH).

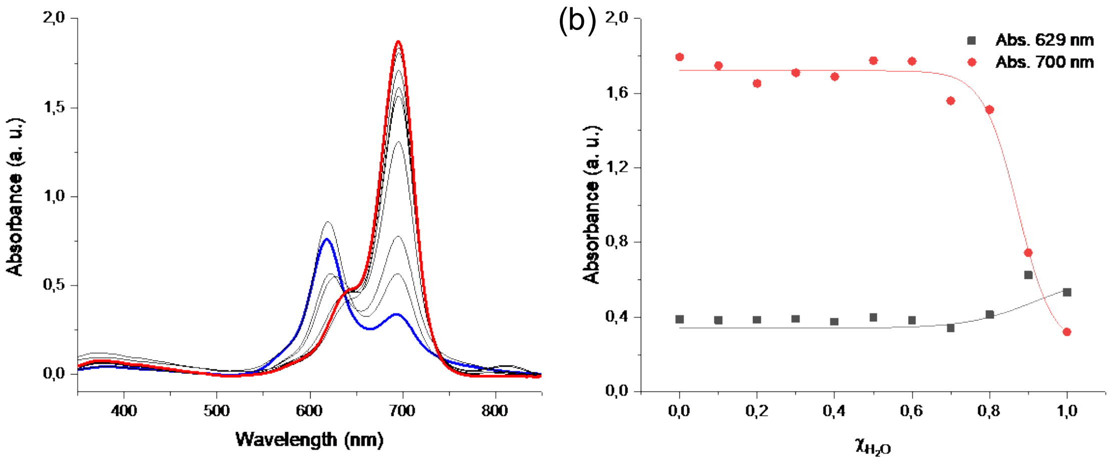

Figure S8.- (a) UV-Vis spectra of ligand **C2** in different solvent mixtures H_2O:MeOH (blue line 100 % H_2O, red line 100 % MeOH). (b) Absorbance of the aggregate and monomer bands versus the percentage of H_2O (X_{H2O}).

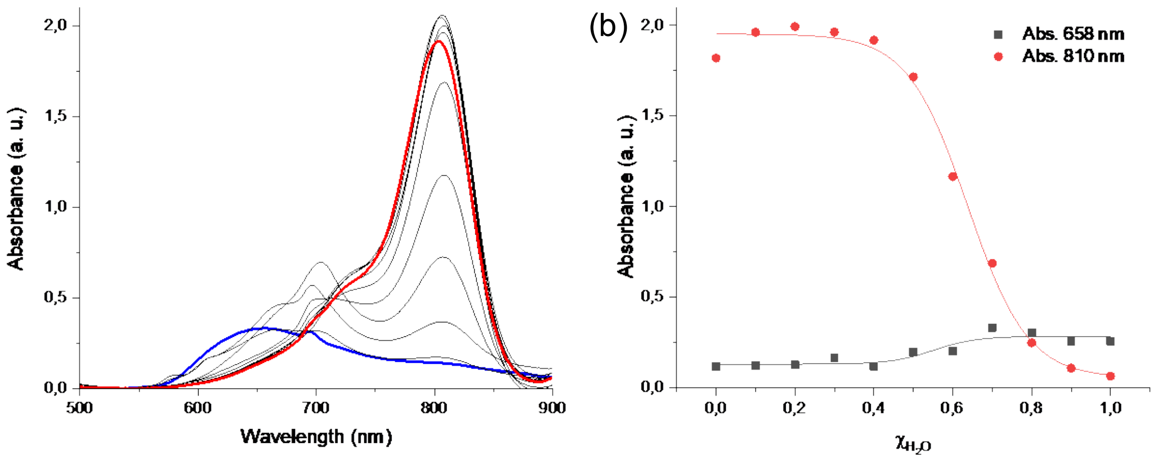

Figure S9.- (a) UV-Vis spectra of ligand **C4** in different solvent mixtures H_2O:MeOH (blue line 100 % H_2O, red line 100 % MeOH). (b) Absorbance of the aggregate and monomer bands versus the percentage of H_2O (X_{H2O}).

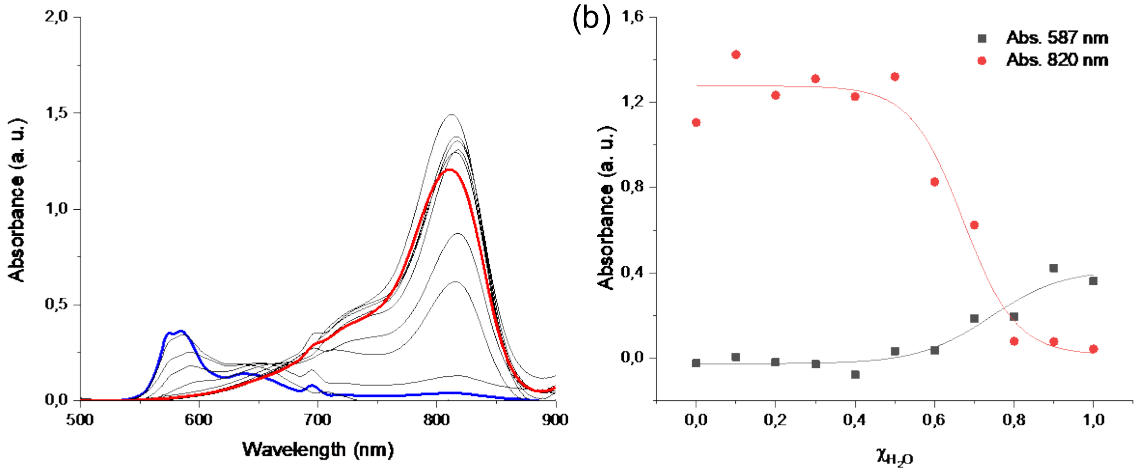

Figure S10.- (a) UV-Vis spectra of ligand **C5** in different solvent mixtures H_2O:MeOH (blue line 100 % H_2O, red line 100 % MeOH). (b) Absorbance of the aggregate and monomer bands versus the percentage of H_2O (X_{H2O}).

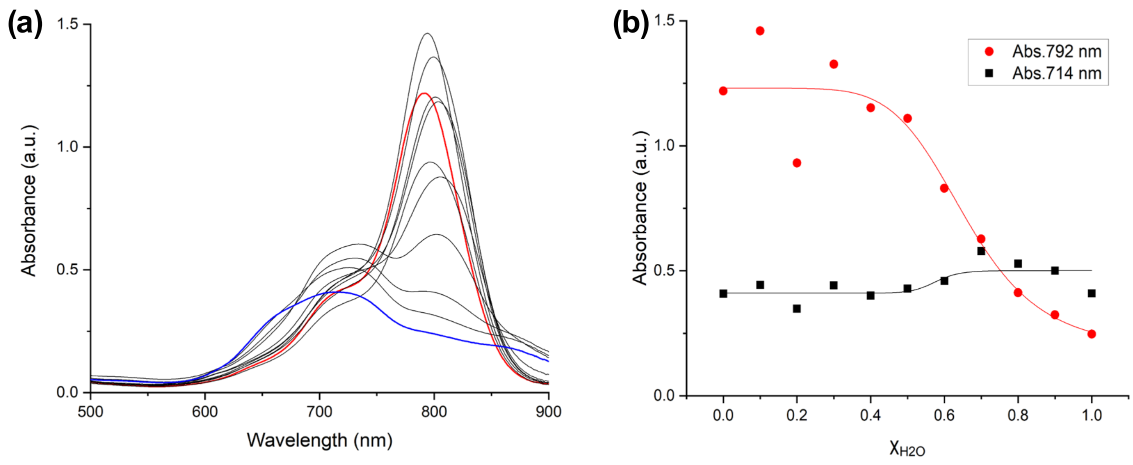

Figure S11.- (a) UV-Vis spectra of ligand **C5-Sulf** in different solvent mixtures H_2O:MeOH (blue line 100 % H_2O, red line 100 % MeOH). (b) Absorbance of the aggregate and monomer bands versus the percentage of H_2O (X_{H2O}).

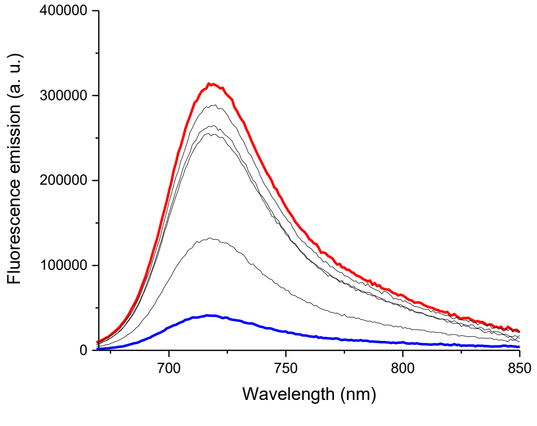

Figure S12.- Fluorescence emission spectra of ligand **C2** in different solvent mixtures H_2O:MeOH (blue line 100 % H_2O, red line 100 % MeOH).

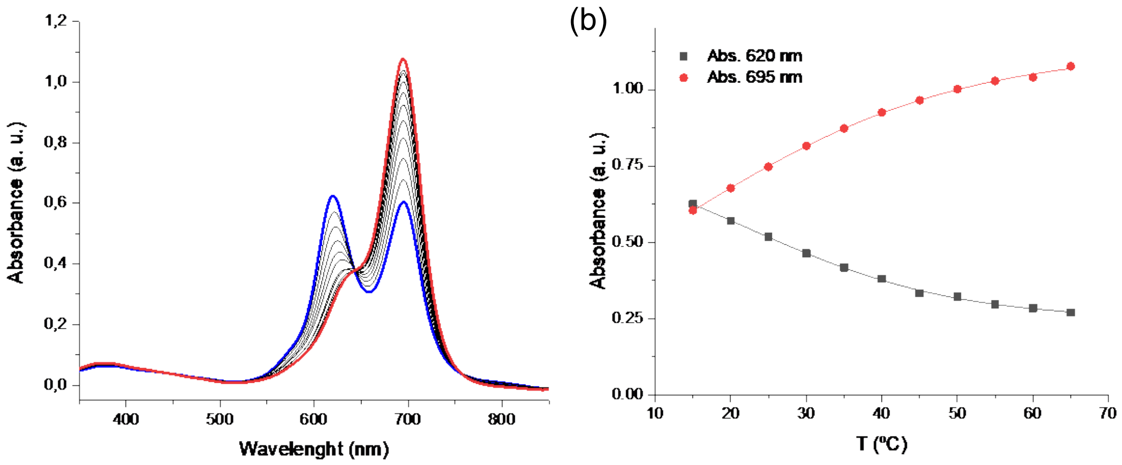

Figure S13.- (a) UV-Vis spectra of ligand **C2** in 80:20 H_2O:MeOH mixture at different temperatures (blue line 15 ºC, red line 65 ºC). (b) Absorbance of the aggregate and monomer species versus the temperature.

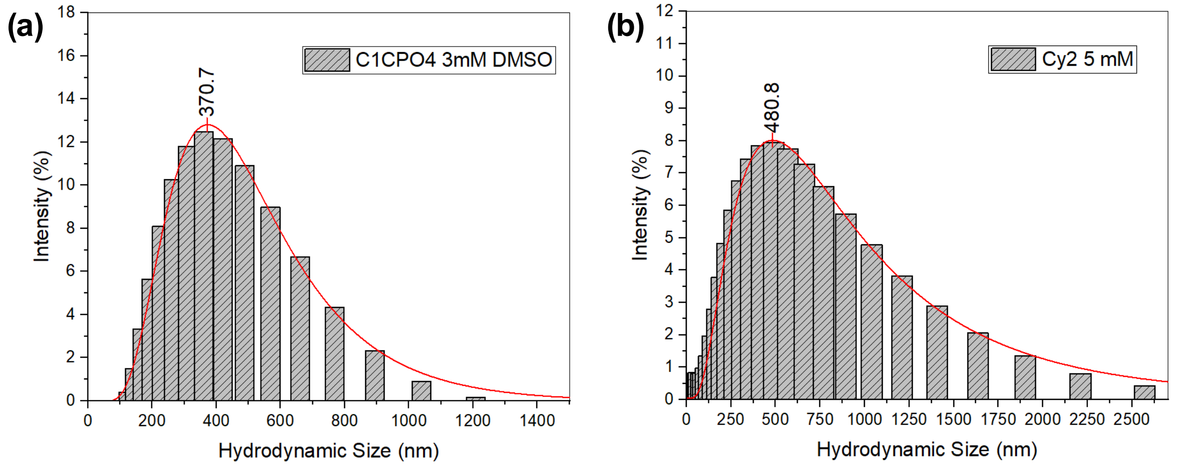

Figure S14.- Averaged dynamic light scattering (DLS) spectra of (a) **C1** and (b) **C2** in water.

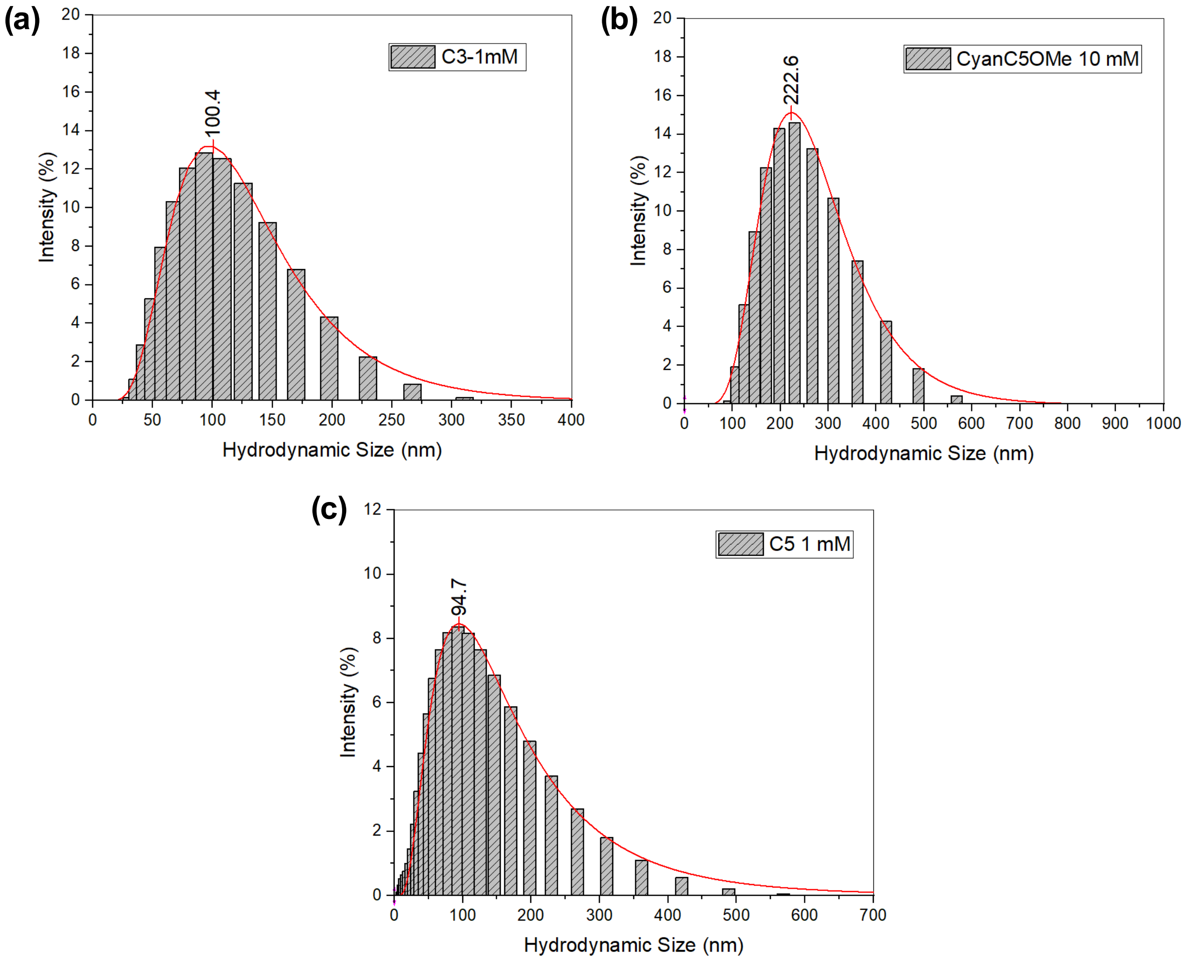

Figure S15.- Averaged dynamic light scattering (DLS) spectra of (a) **C3**, (b) **C4** and (c) **C5** in water.

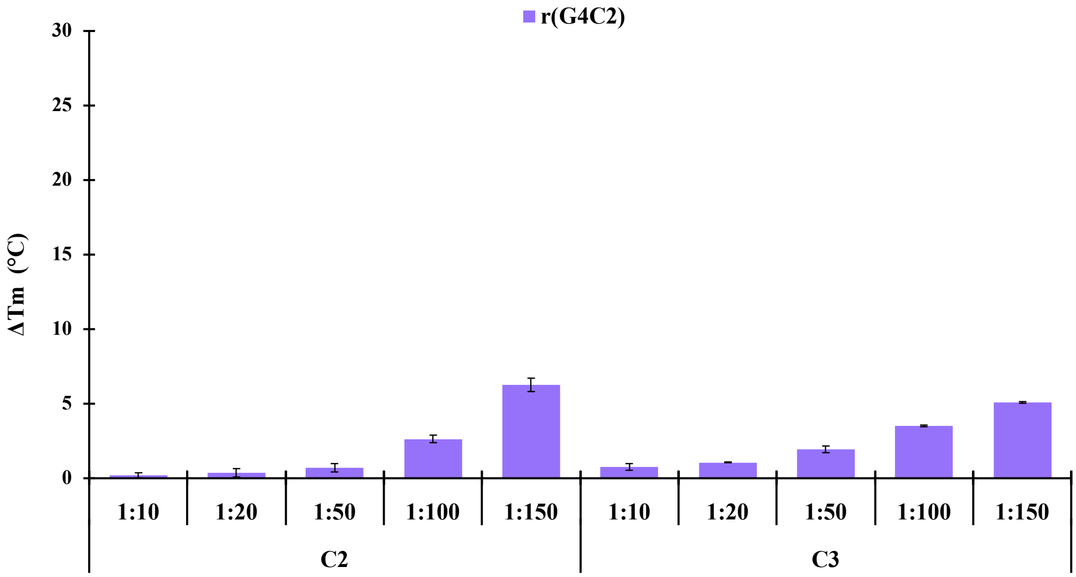

Figure S16.- Plot of ΔT_m (ºC) for the interaction of the cyanine ligands **C2** and **C3** with a G4 RNA (see Table S1 for the sequence of $r(G_4C_2)$) at different molar ratio.

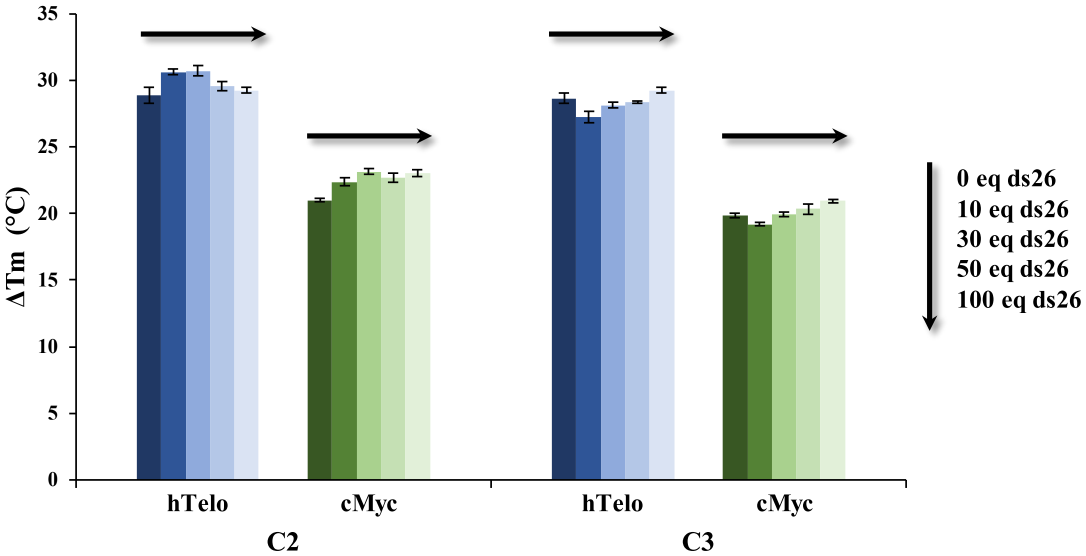

Figure S17.- Plot of ΔT_m (ºC) for FRET competition assays of **C2** and **C3**. The values were determined (in triplicate) by conventional FRET melting assays using 0.2 μM of oligonucleotide and excess of the ligand. The equivalents of the duplex competitor (ds26) used are indicated in the plot.

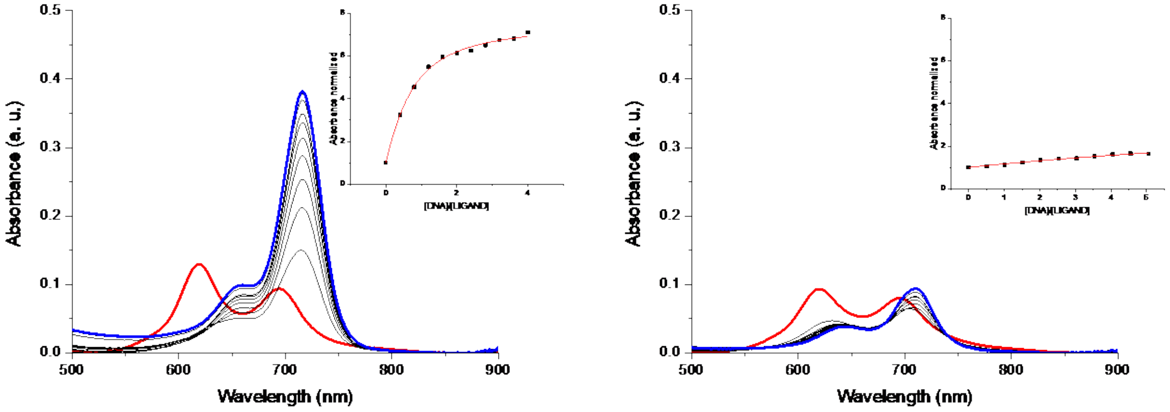

Figure S18.- UV-Vis titrations of **C2** ($c = 1 \times 10^{-5}$ M) with (a) hTelo and (b) ds26 in cacodylate buffer (LiCac 10 mM, KCl 100 mM, pH 7.4). Inset: Fitting of the absorbance at 715 nm normalized versus ratio ([DNA]/[LIGAND].

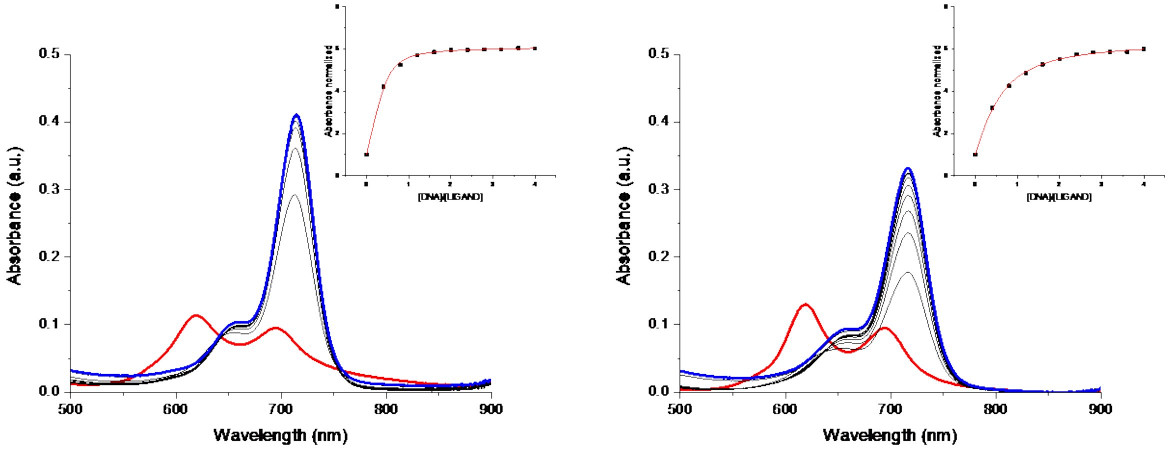

Figure S19.- UV-Vis titrations of **C2** ($c = 1 \times 10^{-5}$ M) with (a) cMyc and (b) 24TTG in cacodylate buffer (LiCac 10 mM, KCl 100 mM, pH 7.4). Inset: Fitting of the absorbance at 715 nm normalized versus ratio ([DNA]/[LIGAND].

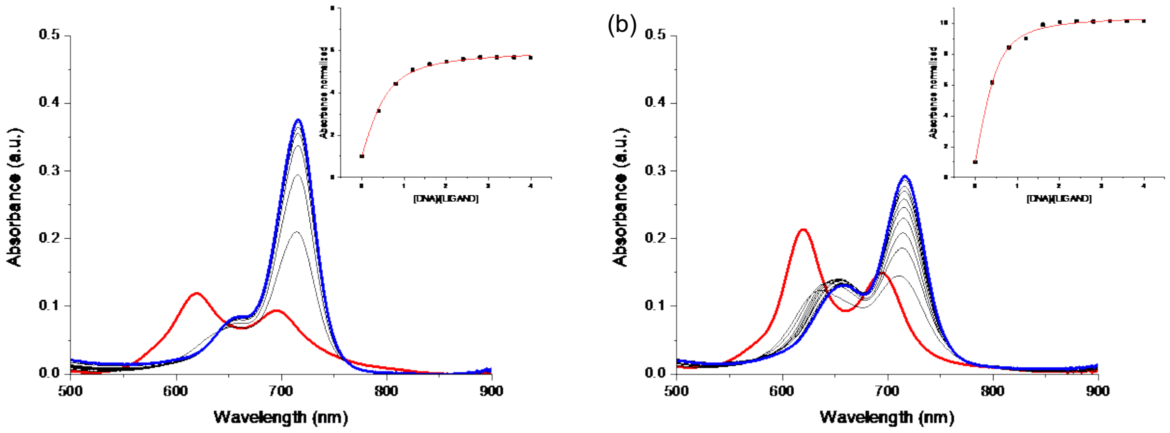

Figure S20.- UV-Vis titrations of **C2** ($c = 1 \times 10^{-5}$ M) with (a) 26TTA and (b) ckit87up in cacodylate buffer (LiCac 10 mM, KCl 100 mM, pH 7.4). Inset: Fitting of the absorbance at 715 nm normalized versus ratio ([DNA]/[LIGAND].

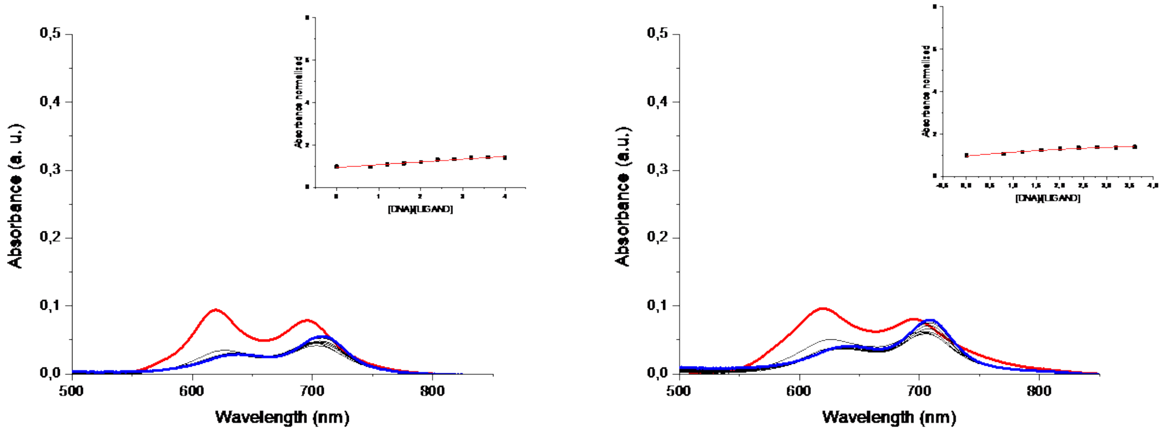

Figure S21.- UV-Vis titrations of **C2** ($c = 1 \times 10^{-5}$ M) with (a) Duplex AT and (b) Duplex GC in cacodylate buffer (LiCac 10 mM, KCl 100 mM, pH 7.4). Inset: Fitting of the absorbance at 715 nm normalized versus ratio ([DNA]/[LIGAND].

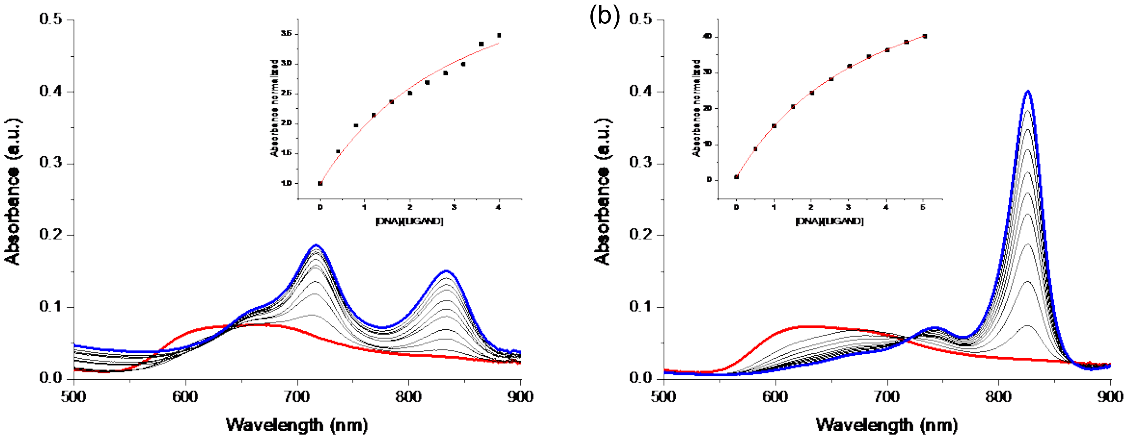

Figure S22.- UV-Vis titrations of **C3** ($c = 1 \times 10^{-5}$ M) with (a) hTelo and (b) ds26 in cacodylate buffer (LiCac 10 mM, KCl 100 mM, pH 7.4). Inset: Fitting of the absorbance at 715 nm normalized versus ratio ([DNA]/[LIGAND].

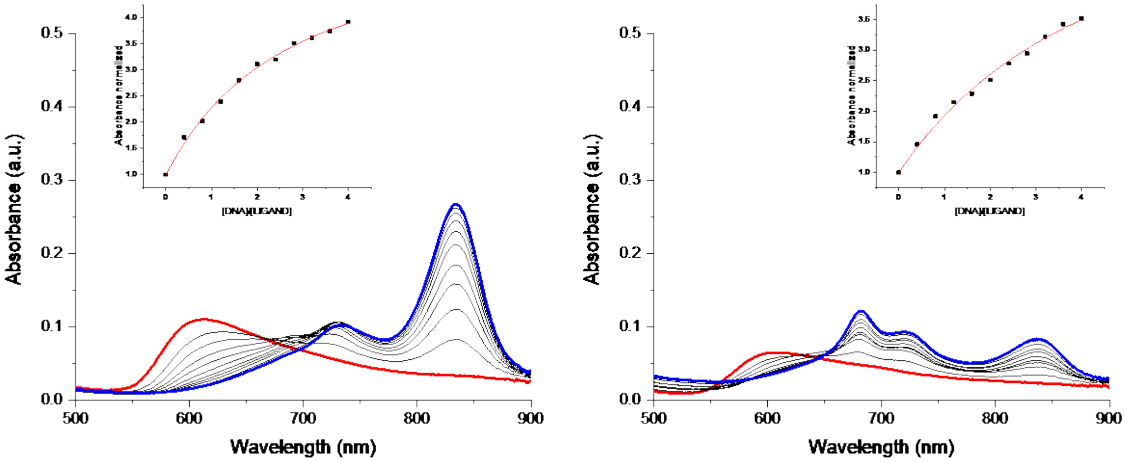

Figure S23.- UV-Vis titrations of **C3** ($c = 1 \times 10^{-5}$ M) with (a) cMyc and (b) 24TTG in cacodylate buffer (LiCac 10 mM, KCl 100 mM, pH 7.4). Inset: Fitting of the absorbance at 715 nm normalized versus ratio ([DNA]/[LIGAND].

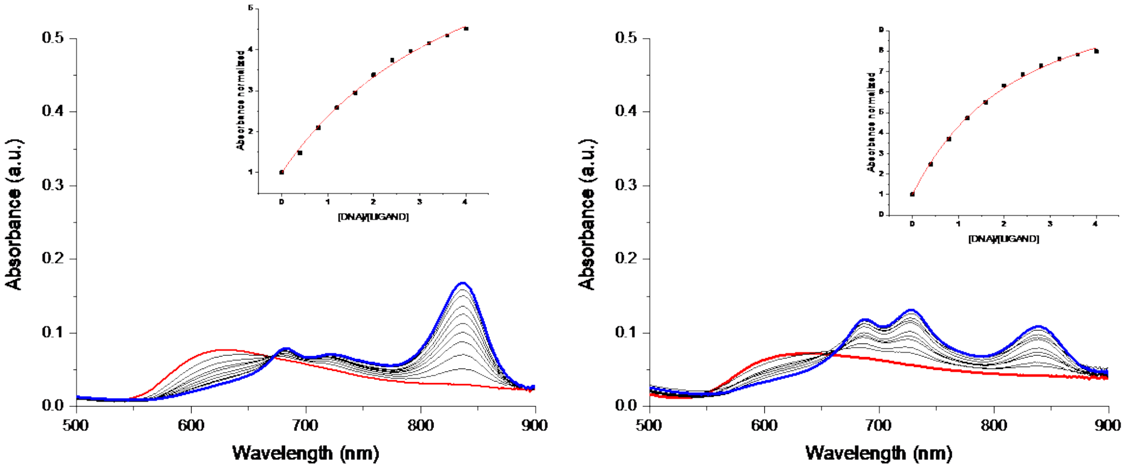

Figure S24.- UV-Vis titrations of **C3** (c = 1 × 10^{-5} M) with (a) 26TTA and (b) ckit87up in cacodylate buffer (LiCac 10 mM, KCl 100 mM, pH 7.4). Inset: Fitting of the absorbance at 715 nm normalized versus ratio ([DNA]/[LIGAND].

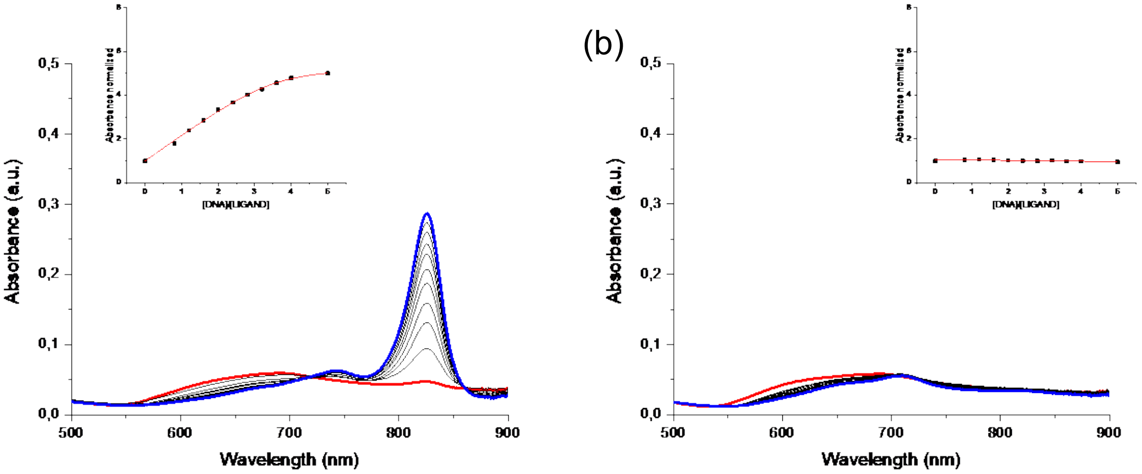

Figure S25.- UV-Vis titrations of **C3** (c = 1 × 10^{-5} M) with (a) 26TTA and (b) ckit87up in cacodylate buffer (LiCac 10 mM, KCl 100 mM, pH 7.4). Inset: Fitting of the absorbance at 715 nm normalized versus ratio ([DNA]/[LIGAND].

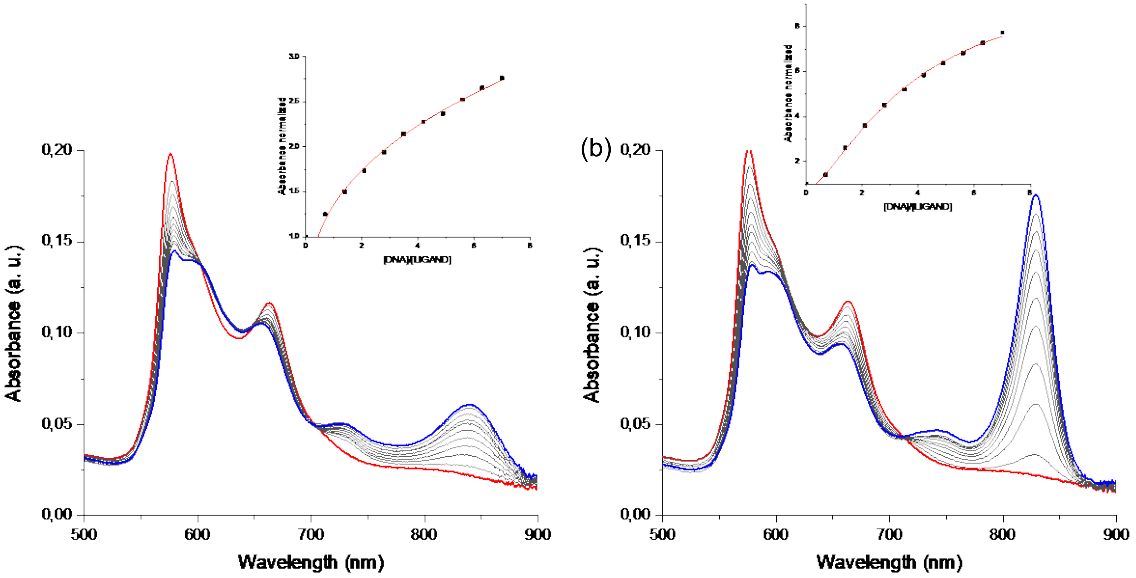

Figure S26.- UV-Vis titrations of **C5** ($c = 1 \times 10^{-5}$ M) with (a) hTelo and (b) ds26 in cacodylate buffer (LiCac 10 mM, KCl 100 mM, pH 7.4). Inset: Fitting of the absorbance at 847 nm normalized versus ratio ([DNA]/[LIGAND].

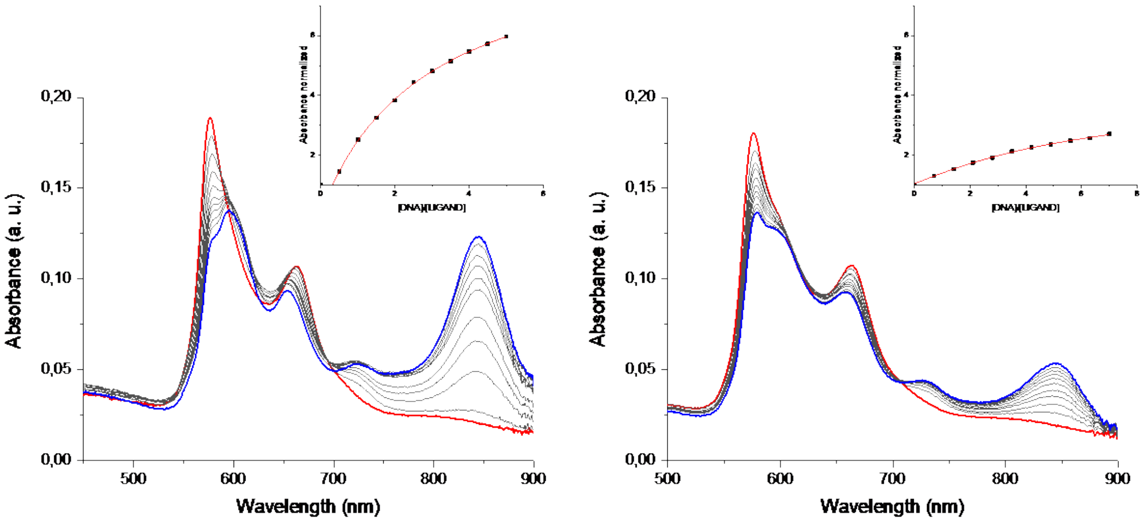

Figure S27.- UV-Vis titrations of **C5** ($c = 1 \times 10^{-5}$ M) with (a) cMyc and (b) 24TTG in cacodylate buffer (LiCac 10 mM, KCl 100 mM, pH 7.4). Inset: Fitting of the absorbance at 847 nm normalized versus ratio ([DNA]/[LIGAND].

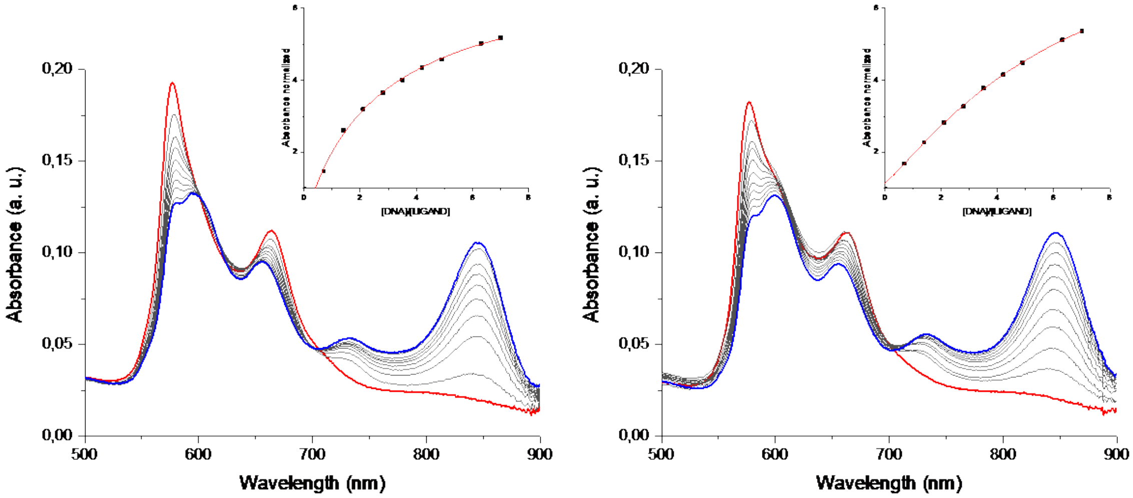

Figure S28.- UV-Vis titrations of **C5** ($c = 1 \times 10^{-5}$ M) with (a) 26TTA and (b) ckit87up in cacodylate buffer (LiCac 10 mM, KCl 100 mM, pH 7.4). Inset: Fitting of the absorbance at 847 nm normalized versus ratio ([DNA]/[LIGAND].

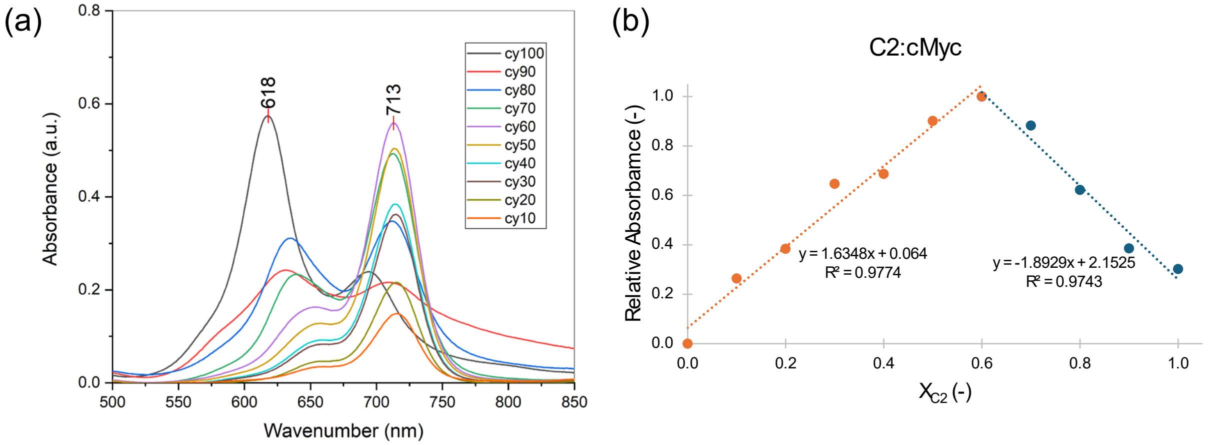

Figure S29.- The Job plot analysis for the binding stoichiometry of **C2** ($c = 1 \times 10^{-5}$ M) to cMyc G4 DNA in Tris 10 mM, KCl 100 mM, pH 7.4. (A) UV-Vis spectra of the Job plot experiment and (B) the points of intersection of the best fit lines for the Job plot.

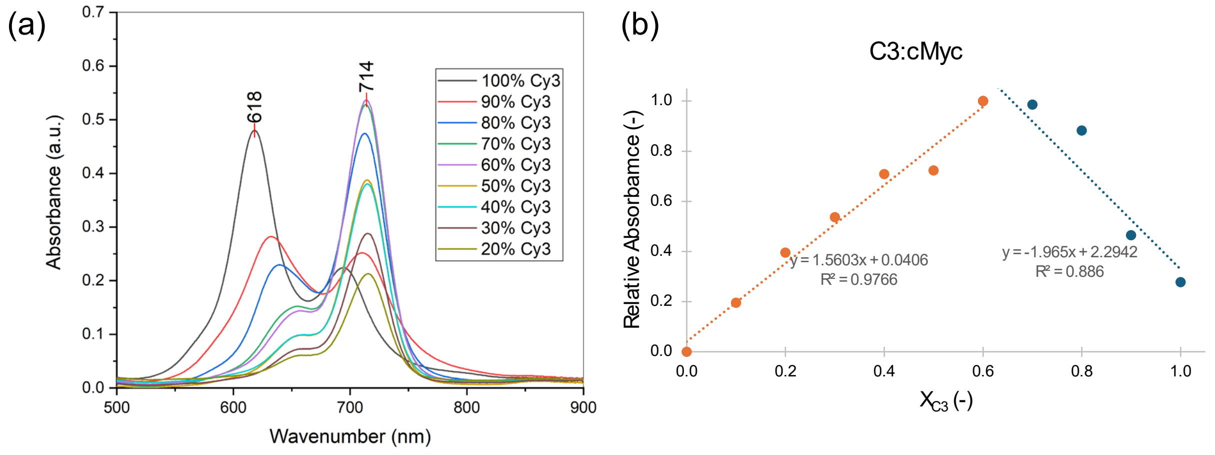

Figure S30.- The Job plot analysis for the binding stoichiometry of **C3** ($c = 1 \times 10^{-5}$ M) to cMyc G4 DNA in Tris 10 mM, KCl 100 mM, pH 7.4. (A) UV-Vis spectra of the Job plot experiment and (B) the points of intersection of the best fit lines for the Job plot.

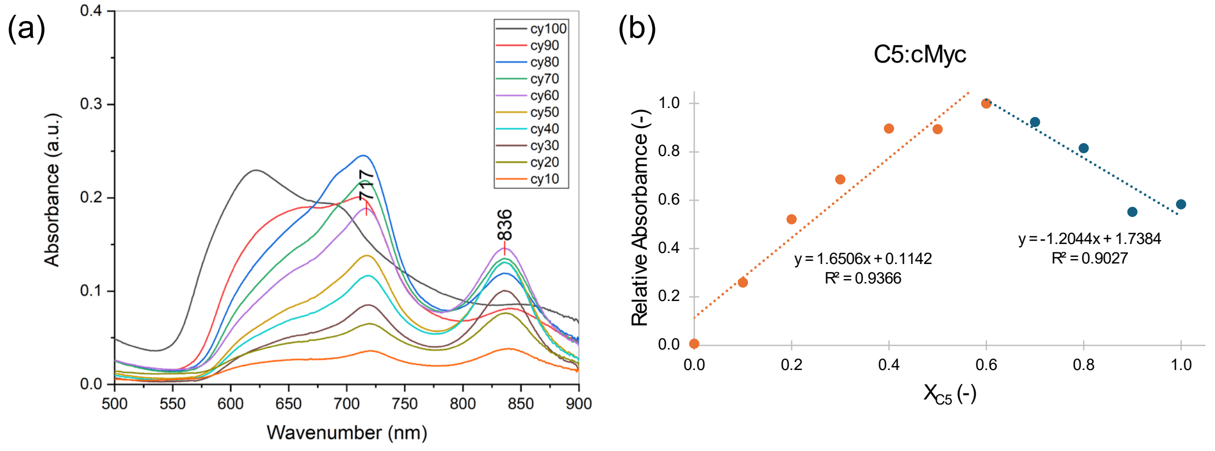

Figure S31.- The Job plot analysis for the binding stoichiometry of **C5** ($c = 1 \times 10^{-5}$ M) to cMyc G4 DNA in Tris 10 mM, KCl 100 mM, pH 7.4. (A) UV-Vis spectra of the Job plot experiment and (B) the points of intersection of the best fit lines for the Job plot.

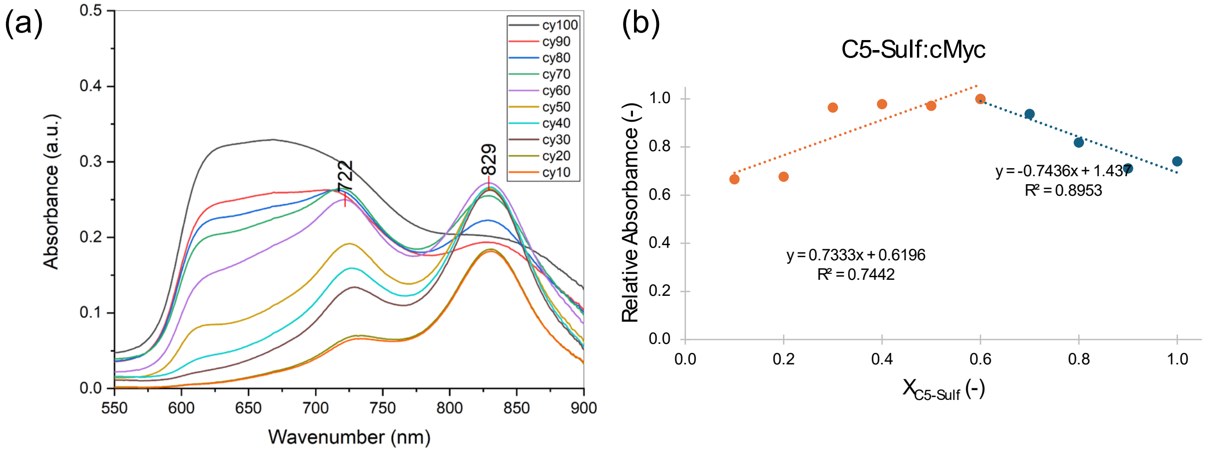

Figure S32.- The Job plot analysis for the binding stoichiometry of **C5-Sulf** ($c = 1 \times 10^{-5}$ M) to cMyc G4 DNA in Tris 10 mM, KCl 100 mM, pH 7.4. (A) UV-Vis spectra of the Job plot experiment and (B) the points of intersection of the best fit lines for the Job plot.

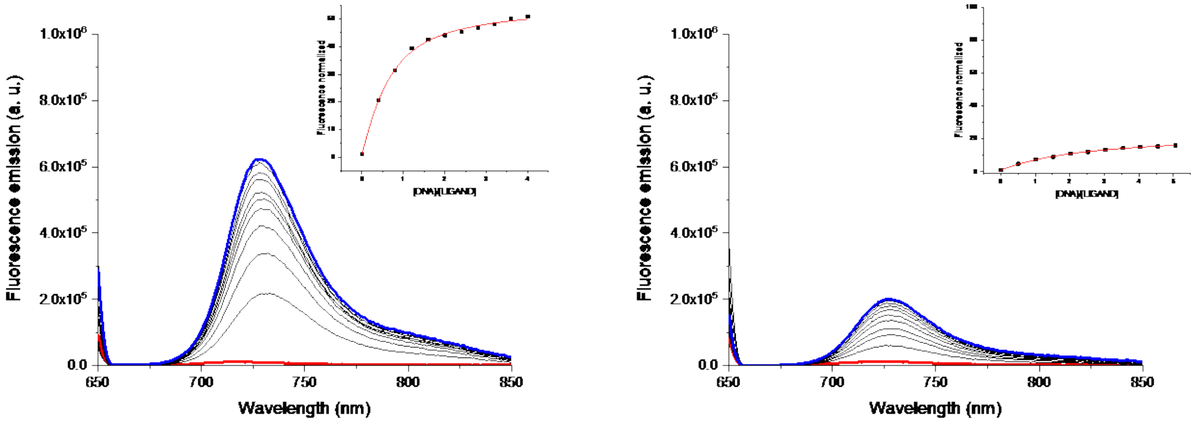

Figure S33.- Fluorimetric titrations of **C2** ($c = 5 \times 10^{-6}$ M) with (a) hTelo and (b) ds26 in cacodylate buffer (LiCac 10 mM, KCl 100 mM, pH 7.4). Inset: Fitting of the fluorescence normalized versus ratio ([DNA]/[LIGAND].

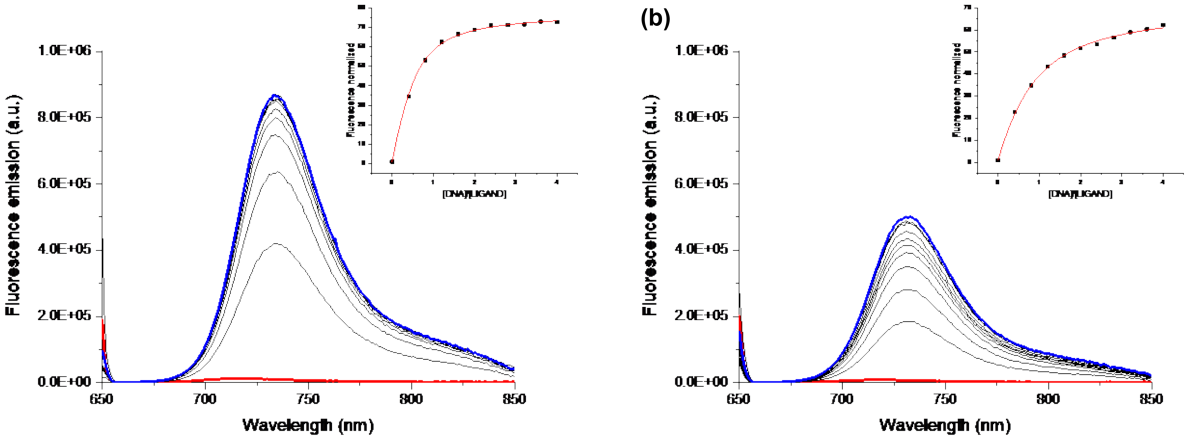

Figure S34.- Fluorimetric titrations of **C2** ($c = 5 \times 10^{-6}$ M) with (a) cMyc and (b) 24TTG in cacodylate buffer (LiCac 10 mM, KCl 100 mM, pH 7.4). Inset: Fitting of the fluorescence normalized versus ratio ([DNA]/[LIGAND].

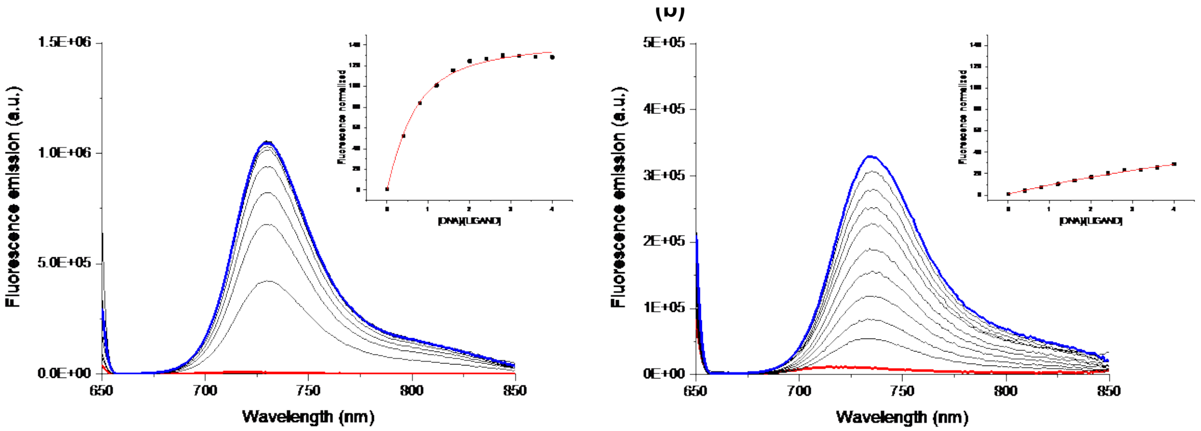

Figure S35.- Fluorimetric titrations of **C2** ($c = 5 \times 10^{-6}$ M) with (a) 26TTA and (b) ckit87up in cacodylate buffer (LiCac 10 mM, KCl 100 mM, pH 7.4). Inset: Fitting of the fluorescence normalized versus ratio ([DNA]/[LIGAND].

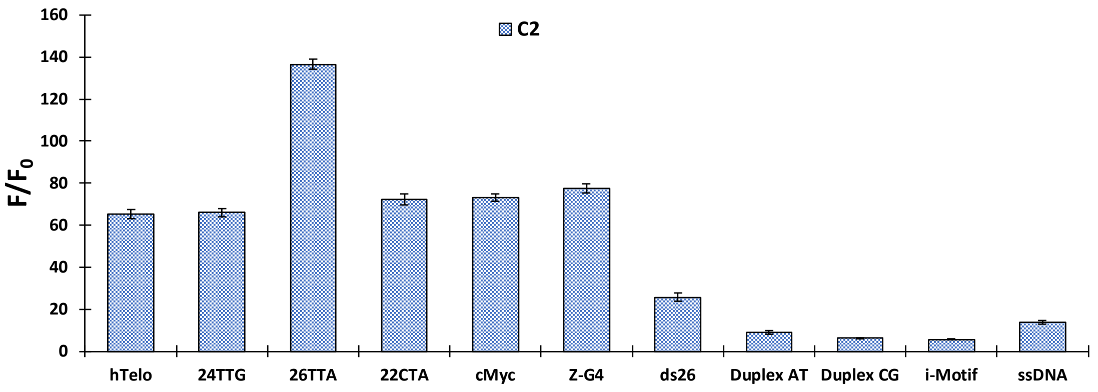

Figure S36.- Emission change experienced upon the addition of different DNA sequences to a solution of **C2** (c = 5 × 10−6 M, λ_{exc}=620 nm) in cacodylate buffer (LiCac 10 mM, KCl 100 mM, pH 7.4).

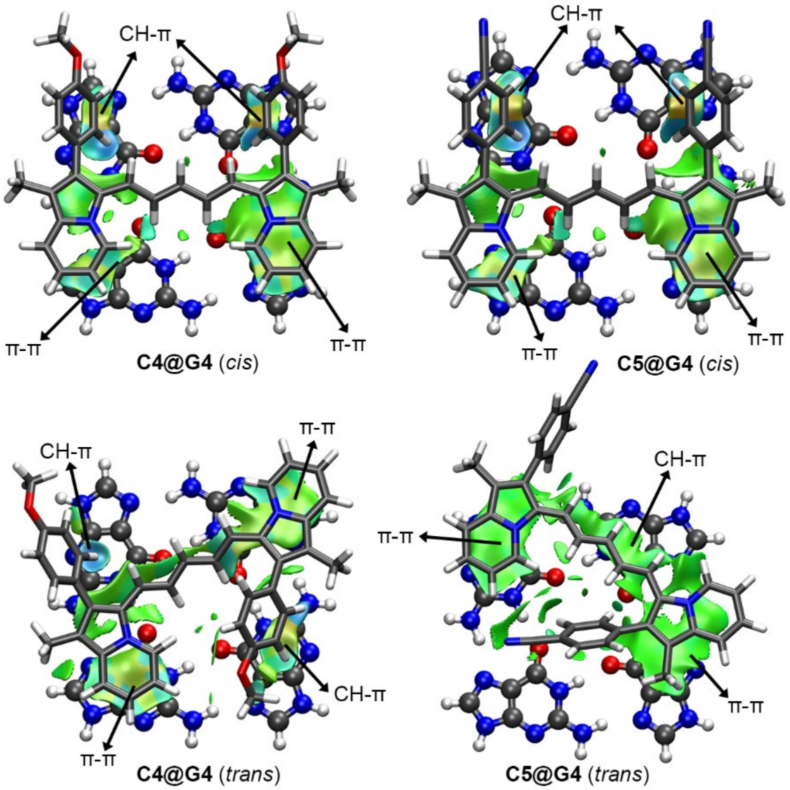

Figure S37. NCIplot surfaces of **C4** and **C5** in both *cis* and *trans* configuration complexed to the G4-quartet. The different types of noncovalent interactions present in each complex are also indicated. The density and RDG cutoff values = 0.5 and 1.0, respectively. The density and RDG cutplot values = 0.07 and 0.3 a.u., respectively. Surfaces created using the fine multigrid option.

Table S3.- Cartesian coordinates of the **C1@G4** (cis) complexes

N	-1.8382209	-6.6252078	-1.6247425
C	-3.0461368	-5.9599997	-1.6230104
N	-2.8953056	-4.6640320	-1.6212551
C	-1.5295612	-4.4611351	-1.6218187
C	-0.7734987	-3.2511352	-1.6203928
O	-1.1926310	-2.0981098	-1.6183650
N	0.6064180	-3.5099890	-1.6215134
C	1.1741154	-4.7579369	-1.6238957
N	2.5142580	-4.8376534	-1.6254969
N	0.4708652	-5.8831967	-1.6251094
C	-0.8510430	-5.6719310	-1.6239858
H	-3.9872128	-6.4862984	-1.6231688
H	1.2193879	-2.6803696	-1.6203083
H	3.1621015	-4.0505143	-1.6226748
H	2.8995166	-5.7651293	-1.6258160
N	6.7877539	-1.9001026	-1.6247425
C	6.1225458	-3.1080185	-1.6230104
N	4.8265781	-2.9571873	-1.6212551
C	4.6236812	-1.5914429	-1.6218187
C	3.4136813	-0.8353804	-1.6203928
O	2.2606559	-1.2545127	-1.6183650
N	3.6725351	0.5445363	-1.6215134
C	4.9204830	1.1122337	-1.6238957
N	5.0001995	2.4523763	-1.6254969
N	6.0457428	0.4089835	-1.6251094
C	5.8344771	-0.9129247	-1.6239858
H	6.6488445	-4.0490945	-1.6231688
H	2.8429157	1.1575062	-1.6203083
H	4.2130604	3.1002198	-1.6226748
H	5.9276754	2.8376349	-1.6258160
N	2.0626487	6.7258722	-1.6247425
C	3.2705646	6.0606641	-1.6230104
N	3.1197334	4.7646964	-1.6212551
C	1.7539890	4.5617995	-1.6218187
C	0.9979265	3.3517996	-1.6203928
O	1.4170588	2.1987742	-1.6183650
N	-0.3819902	3.6106534	-1.6215134
C	-0.9496876	4.8586013	-1.6238957
N	-2.2898302	4.9383178	-1.6254969
N	-0.2464374	5.9838611	-1.6251094
C	1.0754708	5.7725954	-1.6239858
H	4.2116406	6.5869628	-1.6231688
H	-0.9949601	2.7810340	-1.6203083
H	-2.9376737	4.1511787	-1.6226748

H	-2.6750888	5.8657937	-1.6258160
N	-6.5633261	2.0007670	-1.6247425
C	-5.8981180	3.2086829	-1.6230104
N	-4.6021503	3.0578517	-1.6212551
C	-4.3992534	1.6921073	-1.6218187
C	-3.1892535	0.9360448	-1.6203928
O	-2.0362281	1.3551771	-1.6183650
N	-3.4481073	-0.4438719	-1.6215134
C	-4.6960552	-1.0115693	-1.6238957
N	-4.7757717	-2.3517119	-1.6254969
N	-5.8213150	-0.3083191	-1.6251094
C	-5.6100493	1.0135891	-1.6239858
H	-6.4244167	4.1497589	-1.6231688
H	-2.6184879	-1.0568418	-1.6203083
H	-3.9886326	-2.9995554	-1.6226748
H	-5.7032476	-2.7369705	-1.6258160
H	7.7829119	-1.7518877	-1.6262448
H	-1.6900060	-7.6203658	-1.6262448
H	-7.5584841	1.8525521	-1.6262448
H	1.9144338	7.7210302	-1.6262448
C	-1.7285099	-0.0824787	2.2146857
C	-0.6252870	0.7613001	2.1707327
C	0.7511082	0.6744322	2.0499018
C	-3.0928086	0.3399539	2.0271784
C	-3.8980877	-0.7844679	1.9324803
C	-3.0732476	-1.9140223	2.1424678
N	-1.7721376	-1.4717265	2.3850442
N	1.5641002	-0.4279515	1.7392208
C	2.8975280	-0.0664350	1.9225662
C	2.9513561	1.3154472	2.2414120
C	1.6518362	1.7726538	2.3056927
C	-3.3385347	-3.2833547	2.2035589
C	-2.3512454	-4.1527256	2.6129831
C	-1.1043499	-3.6475970	3.0285721
C	-0.8418273	-2.3087412	2.9317549
C	1.2374389	-1.6360173	1.2019705
C	2.1980194	-2.6068581	1.0986710
C	3.5309153	-2.3199742	1.4225012
C	3.8858325	-1.0358799	1.7813230
C	-3.5467331	1.7408409	2.0606217
C	1.2223476	3.1550755	2.5731672
C	-5.3704774	-0.8601351	1.6922142
C	4.2202228	2.0720877	2.4578713
C	-2.9431706	2.7614762	1.3183402
C	-3.3391451	4.0824667	1.4835495
C	-4.3592095	4.4062248	2.3686246
C	-4.9915594	3.3974847	3.0890257
C	-4.5861770	2.0791533	2.9396401

C	1.7013923	4.1850865	1.7567204
C	1.2702945	5.4915085	1.9428416
C	0.3639630	5.7924952	2.9541440
C	-0.1018903	4.7803658	3.7864833
C	0.3246292	3.4716831	3.5997723
H	-0.9334405	1.7959340	2.2467990
H	-4.3293826	-3.6321539	1.9507691
H	-2.5477647	-5.2146806	2.6670929
H	-0.3509951	-4.2974885	3.4491101
H	0.0539519	-1.8543434	3.3174078
H	0.2406963	-1.7442800	0.8101373
H	1.9260147	-3.5719198	0.7044189
H	4.2858245	-3.0846985	1.3096253
H	4.9179756	-0.7569045	1.9387344
H	-5.7462454	0.0741979	1.2813510
H	-5.6105787	-1.6540105	0.9846276
H	-5.9155676	-1.0644238	2.6184050
H	4.0236959	3.0366307	2.9232457
H	4.9075549	1.5169717	3.1000007
H	4.7335815	2.2564030	1.5092634
H	-2.1862269	2.5159562	0.5869699
H	-2.8526119	4.8542839	0.9045191
H	-4.6644070	5.4374677	2.4942687
H	-5.7896231	3.6382450	3.7800545
H	-5.0551480	1.3037604	3.5312772
H	2.3900550	3.9522913	0.9566362
H	1.6346344	6.2717525	1.2874593
H	0.0245219	6.8106937	3.0952480
H	-0.8001062	5.0079970	4.5815439
H	-0.0323237	2.6916801	4.2601068

Table S4.- Cartesian coordinates of the **C1@G4** (trans) complexes

N	-1.8382209	-6.6252078	-1.6247425
C	-3.0461368	-5.9599997	-1.6230104
N	-2.8953056	-4.6640320	-1.6212551
C	-1.5295612	-4.4611351	-1.6218187
C	-0.7734987	-3.2511352	-1.6203928
O	-1.1926310	-2.0981098	-1.6183650
N	0.6064180	-3.5099890	-1.6215134
C	1.1741154	-4.7579369	-1.6238957
N	2.5142580	-4.8376534	-1.6254969
N	0.4708652	-5.8831967	-1.6251094
C	-0.8510430	-5.6719310	-1.6239858
H	-3.9872128	-6.4862984	-1.6231688
H	1.2193879	-2.6803696	-1.6203083
H	3.1621015	-4.0505143	-1.6226748
H	2.8995166	-5.7651293	-1.6258160
N	6.7877539	-1.9001026	-1.6247425
C	6.1225458	-3.1080185	-1.6230104
N	4.8265781	-2.9571873	-1.6212551
C	4.6236812	-1.5914429	-1.6218187
C	3.4136813	-0.8353804	-1.6203928
O	2.2606559	-1.2545127	-1.6183650
N	3.6725351	0.5445363	-1.6215134
C	4.9204830	1.1122337	-1.6238957
N	5.0001995	2.4523763	-1.6254969
N	6.0457428	0.4089835	-1.6251094
C	5.8344771	-0.9129247	-1.6239858
H	6.6488445	-4.0490945	-1.6231688
H	2.8429157	1.1575062	-1.6203083
H	4.2130604	3.1002198	-1.6226748
H	5.9276754	2.8376349	-1.6258160
N	2.0626487	6.7258722	-1.6247425
C	3.2705646	6.0606641	-1.6230104
N	3.1197334	4.7646964	-1.6212551
C	1.7539890	4.5617995	-1.6218187
C	0.9979265	3.3517996	-1.6203928
O	1.4170588	2.1987742	-1.6183650
N	-0.3819902	3.6106534	-1.6215134
C	-0.9496876	4.8586013	-1.6238957
N	-2.2898302	4.9383178	-1.6254969
N	-0.2464374	5.9838611	-1.6251094
C	1.0754708	5.7725954	-1.6239858
H	4.2116406	6.5869628	-1.6231688
H	-0.9949601	2.7810340	-1.6203083
H	-2.9376737	4.1511787	-1.6226748
H	-2.6750888	5.8657937	-1.6258160

N	-6.5633261	2.0007670	-1.6247425
C	-5.8981180	3.2086829	-1.6230104
N	-4.6021503	3.0578517	-1.6212551
C	-4.3992534	1.6921073	-1.6218187
C	-3.1892535	0.9360448	-1.6203928
O	-2.0362281	1.3551771	-1.6183650
N	-3.4481073	-0.4438719	-1.6215134
C	-4.6960552	-1.0115693	-1.6238957
N	-4.7757717	-2.3517119	-1.6254969
N	-5.8213150	-0.3083191	-1.6251094
C	-5.6100493	1.0135891	-1.6239858
H	-6.4244167	4.1497589	-1.6231688
H	-2.6184879	-1.0568418	-1.6203083
H	-3.9886326	-2.9995554	-1.6226748
H	-5.7032476	-2.7369705	-1.6258160
H	7.7829119	-1.7518877	-1.6262448
H	-1.6900060	-7.6203658	-1.6262448
H	-7.5584841	1.8525521	-1.6262448
H	1.9144338	7.7210302	-1.6262448
C	-2.0265199	-0.4170670	3.7066557
C	-0.8690016	0.0460513	3.0888415
C	0.2770560	-0.5616482	2.6029617
C	-3.2898316	0.2656278	3.7120997
C	-4.2302882	-0.5329069	4.3409474
C	-3.5567157	-1.6902634	4.8012550
N	-2.2194282	-1.6050445	4.4270760
N	1.4204590	0.2273469	2.3140570
C	2.4442374	-0.6025589	1.8955716
C	1.9558652	-1.9349986	1.8432803
C	0.6288742	-1.9109631	2.2370473
C	-3.9832759	-2.7898567	5.5524696
C	-3.0671768	-3.7310632	5.9603158
C	-1.7040898	-3.5621514	5.6454318
C	-1.3022386	-2.4993044	4.8896525
C	1.5940043	1.5745660	2.4029006
C	2.7903051	2.1353563	2.0572627
C	3.8600825	1.3157740	1.6486888
C	3.6872058	-0.0455964	1.5787937
C	-3.5030036	1.6019127	3.1421044
C	-0.3219877	-3.0266823	2.1260821
C	-5.6943801	-0.2907963	4.5138990
C	2.7628723	-3.0877210	1.3514121
C	-4.1359022	2.5873877	3.9116233
C	-4.2899434	3.8778092	3.4239641
C	-3.8084899	4.2071905	2.1605313
C	-3.1902843	3.2349940	1.3839971
C	-3.0470303	1.9403636	1.8617959
C	0.0208473	-4.3216569	2.5345008

C	-0.8882216	-5.3652179	2.4210056
C	-2.1570021	-5.1311940	1.9046746
C	-2.5049757	-3.8519691	1.4814150
C	-1.5946983	-2.8126571	1.5810482
H	-0.9190164	1.1161498	2.9255690
H	-5.0281482	-2.8646508	5.8187199
H	-3.3841640	-4.5847001	6.5438148
H	-0.9618637	-4.2667000	5.9897485
H	-0.2736768	-2.3099244	4.6389260
H	0.7570417	2.1620126	2.7442977
H	2.8978313	3.2089880	2.1028376
H	4.8067068	1.7604186	1.3751302
H	4.4844050	-0.6986056	1.2553934
H	-6.0364579	0.5058393	3.8550358
H	-6.2694029	-1.1905548	4.2841396
H	-5.9390892	-0.0017859	5.5401596
H	2.1273950	-3.8537725	0.9134245
H	3.4761479	-2.7767315	0.5915761
H	3.3281928	-3.5540985	2.1642529
H	-4.4815400	2.3424632	4.9078507
H	-4.7730879	4.6286671	4.0363604
H	-3.9173404	5.2148473	1.7800508
H	-2.8283758	3.4788216	0.3993288
H	-2.5928997	1.1912284	1.2259340
H	0.9948144	-4.5058735	2.9680598
H	-0.6055563	-6.3596654	2.7421635
H	-2.8663062	-5.9438573	1.8132477
H	-3.4739662	-3.6715806	1.0364628
H	-1.8558790	-1.8337118	1.2047621

Table S5.- Cartesian coordinates of the **C2@G4** (cis) complexes

N	-1.7883565	-7.2086696	-1.8276163
C	-2.9962724	-6.5434615	-1.8258842
N	-2.8454412	-5.2474938	-1.8241289
C	-1.4796968	-5.0445969	-1.8246925
C	-0.7236343	-3.8345970	-1.8232666
O	-1.1427666	-2.6815716	-1.8212388
N	0.6562824	-4.0934508	-1.8243872
C	1.2239798	-5.3413987	-1.8267695
N	2.5641224	-5.4211152	-1.8283707
N	0.5207296	-6.4666585	-1.8279832
C	-0.8011786	-6.2553928	-1.8268596
H	-3.9373484	-7.0697602	-1.8260426
H	1.2692523	-3.2638314	-1.8231821
H	3.2119659	-4.6339761	-1.8255486
H	2.9493810	-6.3485911	-1.8286898
N	6.8376183	-2.4835644	-1.8276163
C	6.1724102	-3.6914803	-1.8258842
N	4.8764425	-3.5406491	-1.8241289
C	4.6735456	-2.1749047	-1.8246925
C	3.4635457	-1.4188422	-1.8232666
O	2.3105203	-1.8379745	-1.8212388
N	3.7223995	-0.0389255	-1.8243872
C	4.9703474	0.5287719	-1.8267695
N	5.0500639	1.8689145	-1.8283707
N	6.0956072	-0.1744783	-1.8279832
C	5.8843415	-1.4963865	-1.8268596
H	6.6987089	-4.6325563	-1.8260426
H	2.8927801	0.5740444	-1.8231821
H	4.2629248	2.5167580	-1.8255486
H	5.9775398	2.2541731	-1.8286898
N	2.1125131	6.1424104	-1.8276163
C	3.3204290	5.4772023	-1.8258842
N	3.1695978	4.1812346	-1.8241289
C	1.8038534	3.9783377	-1.8246925
C	1.0477909	2.7683378	-1.8232666
O	1.4669232	1.6153124	-1.8212388
N	-0.3321258	3.0271916	-1.8243872
C	-0.8998232	4.2751395	-1.8267695
N	-2.2399658	4.3548560	-1.8283707
N	-0.1965730	5.4003993	-1.8279832
C	1.1253352	5.1891336	-1.8268596
H	4.2615050	6.0035010	-1.8260426
H	-0.9450957	2.1975722	-1.8231821
H	-2.8878093	3.5677169	-1.8255486
H	-2.6252244	5.2823319	-1.8286898

N	-6.5134617	1.4173052	-1.8276163
C	-5.8482536	2.6252211	-1.8258842
N	-4.5522859	2.4743899	-1.8241289
C	-4.3493890	1.1086455	-1.8246925
C	-3.1393891	0.3525830	-1.8232666
O	-1.9863637	0.7717153	-1.8212388
N	-3.3982429	-1.0273337	-1.8243872
C	-4.6461908	-1.5950311	-1.8267695
N	-4.7259073	-2.9351737	-1.8283707
N	-5.7714506	-0.8917809	-1.8279832
C	-5.5601849	0.4301273	-1.8268596
H	-6.3745523	3.5662971	-1.8260426
H	-2.5686235	-1.6403036	-1.8231821
H	-3.9387682	-3.5830172	-1.8255486
H	-5.6533832	-3.3204323	-1.8286898
H	7.8327763	-2.3353495	-1.8291186
H	-1.6401416	-8.2038276	-1.8291186
H	-7.5086197	1.2690903	-1.8291186
H	2.1013757	7.1012747	-1.8943266
C	-2.3527656	-0.5613389	1.4853590
C	-1.0473116	-0.0936253	1.5267616
C	2.6850179	-0.3572224	1.5429131
C	-3.5343687	0.2326795	1.6800153
C	-4.6457958	-0.5918017	1.6357118
C	-4.1787781	-1.9165494	1.4694536
N	-2.7919223	-1.8922213	1.3576974
N	3.2413156	-1.6411457	1.3945569
C	4.6196783	-1.5515772	1.5632114
C	4.9654367	-0.1977434	1.7870018
C	3.7872983	0.5264487	1.8077071
C	-4.8469175	-3.1448795	1.4500624
C	-4.1283499	-4.3107729	1.3516680
C	-2.7244570	-4.2560781	1.2574208
C	-2.0828932	-3.0524565	1.2526454
C	2.6427474	-2.8508046	1.2002284
C	3.3854052	-3.9949521	1.1921332
C	4.7819844	-3.9347448	1.3657876
C	5.3919688	-2.7170553	1.5400775
C	-3.5355284	1.6796125	1.9441763
C	3.6519569	1.9627669	2.0939619
C	-6.0919680	-0.2225993	1.6970363
C	6.3716748	0.2907766	1.9071107
C	-2.8173392	2.5794130	1.1492458
C	-2.8044492	3.9347009	1.4546652
C	-3.5157463	4.4150090	2.5480687
C	-4.2464393	3.5323631	3.3376305
C	-4.2547982	2.1772438	3.0390611
C	2.9325188	2.8083559	1.2448696

C	2.7904774	4.1570602	1.5460825
C	3.3669700	4.6818891	2.6966658
C	4.0942422	3.8514787	3.5450352
C	4.2359948	2.5032641	3.2468167
C	0.1772598	-0.6940402	1.2521757
C	1.3448150	0.0002072	1.5541661
H	-0.9907743	0.9380269	1.8576274
H	-5.9249552	-3.1470002	1.5259596
H	-4.6330584	-5.2669844	1.3401325
H	-2.1378954	-5.1571490	1.1632213
H	-1.0169318	-2.9682413	1.1724504
H	1.5811442	-2.8535099	1.0499133
H	2.8864856	-4.9370428	1.0229439
H	5.3668415	-4.8438391	1.3506664
H	6.4609798	-2.6305070	1.6732077
H	-6.2203355	0.8525248	1.5817537
H	-6.6440710	-0.7184617	0.8956772
H	-6.5486625	-0.5131210	2.6475680
H	6.4019710	1.3772243	1.9601767
H	6.8658146	-0.1040233	2.7992181
H	6.9565440	-0.0163977	1.0361583
H	-2.2898161	2.2129252	0.2795678
H	-2.2472879	4.6156040	0.8260767
H	-3.5026988	5.4716444	2.7838589
H	-4.8013157	3.8978085	4.1925218
H	-4.8023465	1.4899571	3.6713620
H	2.5049620	2.4055617	0.3385805
H	2.2324625	4.7960630	0.8744378
H	3.2531608	5.7324258	2.9326104
H	4.5440937	4.2529335	4.4442684
H	4.7831600	1.8553430	3.9197276
H	0.2161852	-1.6449527	0.7519795
H	1.1976928	1.0209511	1.8913473

Table S6.- Cartesian coordinates of the **C2@G4** (trans) complexes

N	-1.8382209	-6.6252078	-1.6247425
C	-3.0461368	-5.9599997	-1.6230104
N	-2.8953056	-4.6640320	-1.6212551
C	-1.5295612	-4.4611351	-1.6218187
C	-0.7734987	-3.2511352	-1.6203928
O	-1.1926310	-2.0981098	-1.6183650
N	0.6064180	-3.5099890	-1.6215134
C	1.1741154	-4.7579369	-1.6238957
N	2.5142580	-4.8376534	-1.6254969
N	0.4708652	-5.8831967	-1.6251094
C	-0.8510430	-5.6719310	-1.6239858
H	-3.9872128	-6.4862984	-1.6231688
H	1.2193879	-2.6803696	-1.6203083
H	3.1621015	-4.0505143	-1.6226748
H	2.8995166	-5.7651293	-1.6258160
N	6.7877539	-1.9001026	-1.6247425
C	6.1225458	-3.1080185	-1.6230104
N	4.8265781	-2.9571873	-1.6212551
C	4.6236812	-1.5914429	-1.6218187
C	3.4136813	-0.8353804	-1.6203928
O	2.2606559	-1.2545127	-1.6183650
N	3.6725351	0.5445363	-1.6215134
C	4.9204830	1.1122337	-1.6238957
N	5.0001995	2.4523763	-1.6254969
N	6.0457428	0.4089835	-1.6251094
C	5.8344771	-0.9129247	-1.6239858
H	6.6488445	-4.0490945	-1.6231688
H	2.8429157	1.1575062	-1.6203083
H	4.2130604	3.1002198	-1.6226748
H	5.9276754	2.8376349	-1.6258160
N	2.0626487	6.7258722	-1.6247425
C	3.2705646	6.0606641	-1.6230104
N	3.1197334	4.7646964	-1.6212551
C	1.7539890	4.5617995	-1.6218187
C	0.9979265	3.3517996	-1.6203928
O	1.4170588	2.1987742	-1.6183650
N	-0.3819902	3.6106534	-1.6215134
C	-0.9496876	4.8586013	-1.6238957
N	-2.2898302	4.9383178	-1.6254969
N	-0.2464374	5.9838611	-1.6251094
C	1.0754708	5.7725954	-1.6239858
H	4.2116406	6.5869628	-1.6231688
H	-0.9949601	2.7810340	-1.6203083
H	-2.9376737	4.1511787	-1.6226748
H	-2.6750888	5.8657937	-1.6258160

N	-6.5633261	2.0007670	-1.6247425
C	-5.8981180	3.2086829	-1.6230104
N	-4.6021503	3.0578517	-1.6212551
C	-4.3992534	1.6921073	-1.6218187
C	-3.1892535	0.9360448	-1.6203928
O	-2.0362281	1.3551771	-1.6183650
N	-3.4481073	-0.4438719	-1.6215134
C	-4.6960552	-1.0115693	-1.6238957
N	-4.7757717	-2.3517119	-1.6254969
N	-5.8213150	-0.3083191	-1.6251094
C	-5.6100493	1.0135891	-1.6239858
H	-6.4244167	4.1497589	-1.6231688
H	-2.6184879	-1.0568418	-1.6203083
H	-3.9886326	-2.9995554	-1.6226748
H	-5.7032476	-2.7369705	-1.6258160
H	7.7829119	-1.7518877	-1.6262448
H	-1.6900060	-7.6203658	-1.6262448
H	-7.5584841	1.8525521	-1.6262448
H	1.9144338	7.7210302	-1.6262448
C	-1.6534672	2.3114167	1.8858213
C	-0.3673015	1.7961984	1.9198529
C	2.0828885	-0.9999620	1.9171261
C	-2.0119539	3.6970310	2.0356754
C	-3.3866202	3.8187651	1.9356196
C	-3.9165556	2.5149740	1.7836766
N	-2.8654384	1.6051739	1.7467575
N	3.4778579	-1.1482205	1.8182488
C	3.7930946	-2.4989633	1.8552249
C	2.5907417	-3.2352522	2.0002333
C	1.5494126	-2.3221376	2.0611825
C	-5.2320322	2.0490411	1.7117580
C	-5.4738513	0.6988850	1.6243505
C	-4.3910838	-0.1999280	1.6089123
C	-3.1090506	0.2654954	1.6665834
C	4.4449445	-0.1978578	1.6753179
C	5.7582848	-0.5625567	1.6323355
C	6.1157607	-1.9258030	1.6990287
C	5.1367927	-2.8845109	1.7924174
C	-1.0689386	4.7933499	2.3144072
C	0.1471131	-2.6474227	2.3857543
C	-4.2228226	5.0559222	1.9238799
C	2.5294021	-4.7221971	2.1224808
C	-1.3454069	5.6933140	3.3532715
C	-0.4707634	6.7299819	3.6489468
C	0.6996982	6.8903601	2.9129710
C	0.9863722	6.0054507	1.8801947
C	0.1113011	4.9685837	1.5853942
C	-0.5683609	-3.5836236	1.6381633

C	-1.8946184	-3.8745026	1.9381700
C	-2.5160003	-3.2549756	3.0157084
C	-1.8012372	-2.3467771	3.7945102
C	-0.4847534	-2.0427101	3.4806026
C	0.1302631	0.5167470	1.7112632
C	1.4788510	0.2517455	1.8899959
H	0.3741716	2.5443082	2.1753407
H	-6.0377990	2.7686084	1.7338630
H	-6.4857117	0.3240423	1.5595380
H	-4.5478626	-1.2657145	1.5348546
H	-2.2672863	-0.3988932	1.6626788
H	4.1223012	0.8263843	1.5855323
H	6.5082636	0.2046091	1.5125725
H	7.1577584	-2.2109521	1.6570124
H	5.3766739	-3.9375798	1.8147134
H	-3.6059863	5.9402513	1.7747117
H	-4.9535523	5.0115799	1.1126368
H	-4.7766469	5.1877086	2.8578995
H	1.5973542	-5.0385312	2.5888046
H	2.5860718	-5.1967006	1.1389280
H	3.3564599	-5.1045467	2.7239908
H	-2.2423520	5.5633114	3.9444877
H	-0.6997019	7.4094473	4.4601286
H	1.3811273	7.6988941	3.1450923
H	1.8874291	6.1222975	1.2917991
H	0.3355699	4.3022076	0.7681692
H	-0.0863737	-4.0740403	0.8074852
H	-2.4388018	-4.5775138	1.3208235
H	-3.5462182	-3.4845358	3.2574729
H	-2.2726337	-1.8737618	4.6467818
H	0.0623887	-1.3279312	4.0812295
H	-0.4996947	-0.2950782	1.3953549
H	2.1237517	1.1139921	2.0313028

Table S7.- Cartesian coordinates of the **C3@G4** (cis) complexes

N	-1.8642290	-6.5752651	-1.8839522
C	-3.0721449	-5.9100570	-1.8822201
N	-2.9213137	-4.6140893	-1.8804648
C	-1.5555693	-4.4111924	-1.8810284
C	-0.7995068	-3.2011925	-1.8796025
O	-1.2186391	-2.0481671	-1.8775747
N	0.5804099	-3.4600463	-1.8807231
C	1.1481073	-4.7079942	-1.8831054
N	2.4882499	-4.7877107	-1.8847066
N	0.4448571	-5.8332540	-1.8843191
C	-0.8770511	-5.6219883	-1.8831955
H	-4.0132209	-6.4363557	-1.8823785
H	1.1933798	-2.6304269	-1.8795180
H	3.1360934	-4.0005716	-1.8818845
H	2.8735085	-5.7151866	-1.8850257
N	6.7617458	-1.8501599	-1.8839522
C	6.0965377	-3.0580758	-1.8822201
N	4.8005700	-2.9072446	-1.8804648
C	4.5976731	-1.5415002	-1.8810284
C	3.3876732	-0.7854377	-1.8796025
O	2.2346478	-1.2045700	-1.8775747
N	3.6465270	0.5944790	-1.8807231
C	4.8944749	1.1621764	-1.8831054
N	4.9741914	2.5023190	-1.8847066
N	6.0197347	0.4589262	-1.8843191
C	5.8084690	-0.8629820	-1.8831955
H	6.6228364	-3.9991518	-1.8823785
H	2.8169076	1.2074489	-1.8795180
H	4.1870523	3.1501625	-1.8818845
H	5.9016673	2.8875776	-1.8850257
N	2.0366406	6.7758149	-1.8839522
C	3.2445565	6.1106068	-1.8822201
N	3.0937253	4.8146391	-1.8804648
C	1.7279809	4.6117422	-1.8810284
C	0.9719184	3.4017423	-1.8796025
O	1.3910507	2.2487169	-1.8775747
N	-0.4079983	3.6605961	-1.8807231
C	-0.9756957	4.9085440	-1.8831054
N	-2.3158383	4.9882605	-1.8847066
N	-0.2724455	6.0338038	-1.8843191
C	1.0494627	5.8225381	-1.8831955
H	4.1856325	6.6369055	-1.8823785
H	-1.0209682	2.8309767	-1.8795180
H	-2.9636818	4.2011214	-1.8818845
H	-2.7010969	5.9157364	-1.8850257

N	-6.5893342	2.0507097	-1.8839522
C	-5.9241261	3.2586256	-1.8822201
N	-4.6281584	3.1077944	-1.8804648
C	-4.4252615	1.7420500	-1.8810284
C	-3.2152616	0.9859875	-1.8796025
O	-2.0622362	1.4051198	-1.8775747
N	-3.4741154	-0.3939292	-1.8807231
C	-4.7220633	-0.9616266	-1.8831054
N	-4.8017798	-2.3017692	-1.8847066
N	-5.8473231	-0.2583764	-1.8843191
C	-5.6360574	1.0635318	-1.8831955
H	-6.4504248	4.1997016	-1.8823785
H	-2.6444960	-1.0068991	-1.8795180
H	-4.0146407	-2.9496127	-1.8818845
H	-5.7292557	-2.6870278	-1.8850257
H	7.7569038	-1.7019450	-1.8854545
H	-1.7160141	-7.5704231	-1.8854545
H	-7.5844922	1.9024948	-1.8854545
H	1.8884257	7.7709729	-1.8854545
C	-4.9781820	-0.5991898	1.5657905
C	-3.6961890	-0.0493907	1.5611321
C	2.4013626	0.5058636	1.4110691
C	-6.1973957	0.1124698	1.8143157
C	-7.2677512	-0.7574465	1.6634271
C	-6.7344354	-2.0278584	1.3583872
N	-5.3439783	-1.9291810	1.3142372
N	3.1321878	-0.6910219	1.3053360
C	4.4833055	-0.4033851	1.4700522
C	4.6317914	0.9907838	1.6721717
C	3.3621174	1.5378774	1.6831253
C	-7.3392469	-3.2697318	1.1330147
C	-6.5601453	-4.3817179	0.9174080
C	-5.1559343	-4.2586309	0.9304171
C	-4.5743744	-3.0398320	1.1236899
C	2.7059384	-1.9767749	1.1431458
C	3.6009188	-3.0057080	1.1655561
C	4.9744382	-2.7468006	1.3434243
C	5.4096770	-1.4515694	1.4811784
C	-6.2920855	1.5383310	2.1710316
C	3.0142894	2.9353618	1.9945056
C	-8.7312912	-0.4650678	1.7428828
C	5.9457555	1.6687441	1.8765806
C	-7.0613921	1.9308309	3.2739668
C	-7.1675221	3.2696624	3.6269328
C	-6.5082608	4.2442501	2.8837805
C	-5.7429375	3.8684681	1.7852546
C	-5.6357602	2.5292908	1.4324950
C	2.1591573	3.2256319	3.0662967

C	1.8381317	4.5388458	3.3815826
C	2.3662117	5.5851172	2.6310701
C	3.2196379	5.3071344	1.5701841
C	3.5458236	3.9938441	1.2559281
C	-2.4670237	-0.5364072	1.1567325
C	-1.3235899	0.2568264	1.2655929
C	-0.0264479	-0.1572477	1.0116534
C	1.0336555	0.6959953	1.2986945
H	-3.6862100	0.9717243	1.9250455
H	-8.4182496	-3.3320032	1.1549844
H	-7.0188768	-5.3473856	0.7530799
H	-4.5167805	-5.1138946	0.7712604
H	-3.5087788	-2.9093802	1.1383280
H	1.6536602	-2.1301170	1.0002472
H	3.2381827	-4.0122428	1.0206853
H	5.6796466	-3.5660627	1.3560649
H	6.4566440	-1.2151684	1.6069165
H	-8.9177291	0.6068073	1.6980030
H	-9.2679326	-0.9372062	0.9168587
H	-9.1718532	-0.8376946	2.6721406
H	5.8072864	2.6850268	2.2415322
H	6.5625830	1.1299863	2.5998521
H	6.5068502	1.7182095	0.9388821
H	-7.5630105	1.1767820	3.8670159
H	-7.7611494	3.5518189	4.4872848
H	-5.2270668	4.6151066	1.1946538
H	-5.0506808	2.2527953	0.5682117
H	1.7616842	2.4146955	3.6638354
H	1.1822404	4.7460572	4.2177072
H	3.6272630	6.1138005	0.9751403
H	4.1911765	3.7837389	0.4156762
H	-2.3459750	-1.5160633	0.7180437
H	-1.4593484	1.2703810	1.6357403
H	0.1189507	-1.1511482	0.6153827
H	0.7468587	1.7188612	1.5149869
H	2.1150600	6.6096007	2.8749032
H	-6.5908180	5.2876294	3.1605010

Table S8.- Cartesian coordinates of the **C3@G4** (trans) complexes

N	-1.9504348	-6.6755400	-0.0017551
C	-3.1583507	-6.0103319	-0.0000230
N	-3.0075195	-4.7143642	0.0017323
C	-1.6417751	-4.5114673	0.0011687
C	-0.8857126	-3.3014674	0.0025946
O	-1.3048449	-2.1484420	0.0046224
N	0.4942041	-3.5603212	0.0014740
C	1.0619015	-4.8082691	-0.0009083
N	2.4020441	-4.8879856	-0.0025095
N	0.3586513	-5.9335289	-0.0021220
C	-0.9632569	-5.7222632	-0.0009984
H	-4.0994267	-6.5366306	-0.0001814
H	1.1071740	-2.7307018	0.0026791
H	3.0498876	-4.1008465	0.0003126
H	2.7873027	-5.8154615	-0.0028286
N	6.6755400	-1.9504348	-0.0017551
C	6.0103319	-3.1583507	-0.0000230
N	4.7143642	-3.0075195	0.0017323
C	4.5114673	-1.6417751	0.0011687
C	3.3014674	-0.8857126	0.0025946
O	2.1484420	-1.3048449	0.0046224
N	3.5603212	0.4942041	0.0014740
C	4.8082691	1.0619015	-0.0009083
N	4.8879856	2.4020441	-0.0025095
N	5.9335289	0.3586513	-0.0021220
C	5.7222632	-0.9632569	-0.0009984
H	6.5366306	-4.0994267	-0.0001814
H	2.7307018	1.1071740	0.0026791
H	4.1008465	3.0498876	0.0003126
H	5.8154615	2.7873027	-0.0028286
N	1.9504348	6.6755400	-0.0017551
C	3.1583507	6.0103319	-0.0000230
N	3.0075195	4.7143642	0.0017323
C	1.6417751	4.5114673	0.0011687
C	0.8857126	3.3014674	0.0025946
O	1.3048449	2.1484420	0.0046224
N	-0.4942041	3.5603212	0.0014740
C	-1.0619015	4.8082691	-0.0009083
N	-2.4020441	4.8879856	-0.0025095
N	-0.3586513	5.9335289	-0.0021220
C	0.9632569	5.7222632	-0.0009984
H	4.0994267	6.5366306	-0.0001814
H	-1.1071740	2.7307018	0.0026791
H	-3.0498876	4.1008465	0.0003126
H	-2.7873027	5.8154615	-0.0028286

N	-6.6755400	1.9504348	-0.0017551
C	-6.0103319	3.1583507	-0.0000230
N	-4.7143642	3.0075195	0.0017323
C	-4.5114673	1.6417751	0.0011687
C	-3.3014674	0.8857126	0.0025946
O	-2.1484420	1.3048449	0.0046224
N	-3.5603212	-0.4942041	0.0014740
C	-4.8082691	-1.0619015	-0.0009083
N	-4.8879856	-2.4020441	-0.0025095
N	-5.9335289	-0.3586513	-0.0021220
C	-5.7222632	0.9632569	-0.0009984
H	-6.5366306	4.0994267	-0.0001814
H	-2.7307018	-1.1071740	0.0026791
H	-4.1008465	-3.0498876	0.0003126
H	-5.8154615	-2.7873027	-0.0028286
H	7.6706980	-1.8022199	-0.0032574
H	-1.8022199	-7.6706980	-0.0032574
H	-7.6706980	1.8022199	-0.0032574
H	1.8022199	7.6706980	-0.0032574
C	-1.6952883	-0.9887987	3.3465458
C	-1.6471880	0.3937121	3.3407453
C	1.2611130	5.8341127	3.3423607
C	-2.8339920	-1.8020132	3.6980700
C	-2.4379057	-3.1246066	3.7569384
C	-1.0490589	-3.1660229	3.4696583
N	-0.6128639	-1.8794463	3.1871850
N	1.0774463	7.1903130	3.0386242
C	2.3040897	7.8477770	3.1352684
C	3.2807007	6.9041922	3.5204210
C	2.6441180	5.6777961	3.6454946
C	-0.1332573	-4.2204344	3.4579346
C	1.1870706	-3.9687509	3.1642938
C	1.5795297	-2.6705417	2.7949681
C	0.6665179	-1.6532899	2.7756681
C	-0.0415094	7.8460577	2.6136175
C	0.0169802	9.1758516	2.3144450
C	1.2380039	9.8791577	2.4258916
C	2.3706087	9.2132422	2.8299923
C	-4.1678703	-1.2896986	4.0556622
C	3.2878132	4.4153449	4.0696959
C	-3.2546741	-4.3465084	4.0233661
C	4.7217652	7.2173703	3.7563105
C	-4.7893339	-1.7354185	5.2301180
C	-6.0488767	-1.2749096	5.5884692
C	-6.7160171	-0.3601425	4.7793662
C	-6.1084462	0.0950185	3.6149511
C	-4.8462704	-0.3612353	3.2605047
C	3.0774637	3.9094747	5.3550194

C	3.6564613	2.7068305	5.7406273
C	4.4500331	1.9938060	4.8461061
C	4.6689531	2.4938291	3.5670003
C	4.0951300	3.7009140	3.1830067
C	-0.5703830	1.2752743	3.2690395
C	-0.7336586	2.6475287	3.2953432
C	0.3463444	3.5344175	3.3160390
C	0.2121357	4.9040156	3.2918729
H	-2.6175014	0.8563459	3.4758121
H	-0.4824005	-5.2169758	3.6869304
H	1.9093942	-4.7729148	3.1567086
H	2.5831968	-2.4698430	2.4526314
H	0.8988970	-0.6829824	2.3774159
H	-0.9385423	7.2617913	2.4981274
H	-0.8813301	9.6742492	1.9806041
H	1.2770879	10.9332561	2.1875192
H	3.3233379	9.7177671	2.9153798
H	-4.3183057	-4.1273028	3.9519865
H	-3.0219612	-5.1252776	3.2933839
H	-3.0631174	-4.7593347	5.0183330
H	5.2606752	6.3291741	4.0830699
H	5.2051370	7.5867766	2.8469852
H	4.8440393	7.9874256	4.5224489
H	-4.2704904	-2.4331535	5.8743202
H	-6.5083917	-1.6280720	6.5029579
H	-7.7008085	-0.0061289	5.0565290
H	-6.6194264	0.7979775	2.9696367
H	-4.3904079	-0.0073097	2.3503419
H	2.4502375	4.4576699	6.0470852
H	3.4883830	2.3262871	6.7402812
H	4.8982025	1.0554353	5.1478783
H	5.2816947	1.9465583	2.8617492
H	4.2414969	4.0777787	2.1810274
H	0.4459407	0.9126143	3.2301902
H	-1.7426414	3.0536838	3.2929740
H	1.3376318	3.1028050	3.3288438
H	-0.7951390	5.2964752	3.1963822

Table S9.- Cartesian coordinates of the **C4@G4** (cis) complexes

N	-1.8382209	-6.6252078	-1.6247425
C	-3.0461368	-5.9599997	-1.6230104
N	-2.8953056	-4.6640320	-1.6212551
C	-1.5295612	-4.4611351	-1.6218187
C	-0.7734987	-3.2511352	-1.6203928
O	-1.1926310	-2.0981098	-1.6183650
N	0.6064180	-3.5099890	-1.6215134
C	1.1741154	-4.7579369	-1.6238957
N	2.5142580	-4.8376534	-1.6254969
N	0.4708652	-5.8831967	-1.6251094
C	-0.8510430	-5.6719310	-1.6239858
H	-3.9872128	-6.4862984	-1.6231688
H	1.2193879	-2.6803696	-1.6203083
H	3.1621015	-4.0505143	-1.6226748
H	2.8995166	-5.7651293	-1.6258160
N	6.7877539	-1.9001026	-1.6247425
C	6.1225458	-3.1080185	-1.6230104
N	4.8265781	-2.9571873	-1.6212551
C	4.6236812	-1.5914429	-1.6218187
C	3.4136813	-0.8353804	-1.6203928
O	2.2606559	-1.2545127	-1.6183650
N	3.6725351	0.5445363	-1.6215134
C	4.9204830	1.1122337	-1.6238957
N	5.0001995	2.4523763	-1.6254969
N	6.0457428	0.4089835	-1.6251094
C	5.8344771	-0.9129247	-1.6239858
H	6.6488445	-4.0490945	-1.6231688
H	2.8429157	1.1575062	-1.6203083
H	4.2130604	3.1002198	-1.6226748
H	5.9276754	2.8376349	-1.6258160
N	2.0626487	6.7258722	-1.6247425
C	3.2705646	6.0606641	-1.6230104
N	3.1197334	4.7646964	-1.6212551
C	1.7539890	4.5617995	-1.6218187
C	0.9979265	3.3517996	-1.6203928
O	1.4170588	2.1987742	-1.6183650
N	-0.3819902	3.6106534	-1.6215134
C	-0.9496876	4.8586013	-1.6238957
N	-2.2898302	4.9383178	-1.6254969
N	-0.2464374	5.9838611	-1.6251094
C	1.0754708	5.7725954	-1.6239858
H	4.2116406	6.5869628	-1.6231688
H	-0.9949601	2.7810340	-1.6203083
H	-2.9376737	4.1511787	-1.6226748
H	-2.6750888	5.8657937	-1.6258160

N	-6.5633261	2.0007670	-1.6247425
C	-5.8981180	3.2086829	-1.6230104
N	-4.6021503	3.0578517	-1.6212551
C	-4.3992534	1.6921073	-1.6218187
C	-3.1892535	0.9360448	-1.6203928
O	-2.0362281	1.3551771	-1.6183650
N	-3.4481073	-0.4438719	-1.6215134
C	-4.6960552	-1.0115693	-1.6238957
N	-4.7757717	-2.3517119	-1.6254969
N	-5.8213150	-0.3083191	-1.6251094
C	-5.6100493	1.0135891	-1.6239858
H	-6.4244167	4.1497589	-1.6231688
H	-2.6184879	-1.0568418	-1.6203083
H	-3.9886326	-2.9995554	-1.6226748
H	-5.7032476	-2.7369705	-1.6258160
H	7.7829119	-1.7518877	-1.6262448
H	-1.6900060	-7.6203658	-1.6262448
H	-7.5584841	1.8525521	-1.6262448
H	1.9144338	7.7210302	-1.6262448
C	-3.2846689	-2.1074838	1.9194824
C	-2.2257789	-1.2031167	1.9582107
C	3.6019826	0.8082065	1.8971000
C	-4.6723897	-1.7988031	2.1151052
C	-5.4099312	-2.9725187	2.0252568
C	-4.4968790	-4.0292384	1.8267302
N	-3.2089477	-3.5000768	1.7486932
N	4.5511728	-0.2203514	1.7568540
C	5.8259285	0.3347483	1.8517663
C	5.7020222	1.7296884	2.0279910
C	4.3468950	2.0198878	2.0901186
C	-4.6704542	-5.4169607	1.7589142
C	-3.5746120	-6.2394949	1.6611143
C	-2.2821820	-5.6767369	1.6283595
C	-2.1228197	-4.3226042	1.6685497
C	4.3849640	-1.5726456	1.6749196
C	5.4681433	-2.4023924	1.6641861
C	6.7707892	-1.8670235	1.7235487
C	6.9429800	-0.5073826	1.8115020
C	-5.2131605	-0.4549193	2.3491091
C	3.7492945	3.3402069	2.3170095
C	-6.8936151	-3.1471539	2.0672454
C	6.8614144	2.6706720	2.0702104
C	-6.1867966	-0.2347251	3.3274227
C	-6.7264211	1.0266744	3.5496348
C	-6.2987667	2.1100220	2.7779868
C	-5.3221460	1.9092654	1.7979051
C	-4.7927768	0.6509586	1.5956898
C	2.6960511	3.8208934	1.5252413

C	2.1331894	5.0597781	1.7584360
C	2.6085373	5.8674417	2.7954200
C	3.6606323	5.4104615	3.5927947
C	4.2174786	4.1619613	3.3457793
C	-0.8991765	-1.3166753	1.5745985
C	0.0114446	-0.2944840	1.8258970
C	1.3726588	-0.3530980	1.5510264
C	2.2169790	0.6841912	1.9212255
H	-2.5152913	-0.2358404	2.3546666
H	-5.6728933	-5.8172590	1.8188993
H	-3.6978908	-7.3136784	1.6273122
H	-1.4027236	-6.2997955	1.5613907
H	-1.1552392	-3.8570579	1.6606670
H	3.3769388	-1.9412828	1.6333109
H	5.3042303	-3.4668711	1.5919743
H	7.6271337	-2.5276351	1.7089974
H	7.9264494	-0.0638672	1.8759167
H	-7.3981792	-2.2166741	1.8097963
H	-7.2170540	-3.9128445	1.3588849
H	-7.2452934	-3.4520223	3.0571755
H	6.5346962	3.6925553	1.8818860
H	7.3676207	2.6585679	3.0399669
H	7.6014637	2.4052793	1.3119324
H	-6.5188435	-1.0590483	3.9449225
H	-7.4694635	1.1526079	4.3230798
H	-5.0012724	2.7480415	1.1946093
H	-4.0605665	0.5130313	0.8165379
H	2.3397381	3.2240294	0.6978055
H	1.3293813	5.4313181	1.1372220
H	4.0452479	6.0080613	4.4060346
H	5.0185830	3.8117333	3.9844548
H	-0.5368090	-2.1844362	1.0438964
H	-0.3635570	0.6009715	2.3164702
H	1.7389136	-1.2153107	1.0115509
H	1.7251306	1.5750570	2.2971466
O	1.9938235	7.0667810	2.9420126
O	-6.7665806	3.3768863	2.8984009
C	2.4105071	7.9305482	3.9928131
H	1.7771689	8.8115980	3.9232349
H	2.2724337	7.4618490	4.9718861
H	3.4575037	8.2263800	3.8751383
C	-7.7576295	3.6540984	3.8809037
H	-7.3893300	3.4417541	4.8890313
H	-7.9751425	4.7158485	3.7935060
H	-8.6713098	3.0802075	3.6985134

Table S10.- Cartesian coordinates of the **C4@G4** (trans) complexes

N	-1.9504348	-6.6755400	-0.0017551
C	-3.1583507	-6.0103319	-0.0000230
N	-3.0075195	-4.7143642	0.0017323
C	-1.6417751	-4.5114673	0.0011687
C	-0.8857126	-3.3014674	0.0025946
O	-1.3048449	-2.1484420	0.0046224
N	0.4942041	-3.5603212	0.0014740
C	1.0619015	-4.8082691	-0.0009083
N	2.4020441	-4.8879856	-0.0025095
N	0.3586513	-5.9335289	-0.0021220
C	-0.9632569	-5.7222632	-0.0009984
H	-4.0994267	-6.5366306	-0.0001814
H	1.1071740	-2.7307018	0.0026791
H	3.0498876	-4.1008465	0.0003126
H	2.7873027	-5.8154615	-0.0028286
N	6.6755400	-1.9504348	-0.0017551
C	6.0103319	-3.1583507	-0.0000230
N	4.7143642	-3.0075195	0.0017323
C	4.5114673	-1.6417751	0.0011687
C	3.3014674	-0.8857126	0.0025946
O	2.1484420	-1.3048449	0.0046224
N	3.5603212	0.4942041	0.0014740
C	4.8082691	1.0619015	-0.0009083
N	4.8879856	2.4020441	-0.0025095
N	5.9335289	0.3586513	-0.0021220
C	5.7222632	-0.9632569	-0.0009984
H	6.5366306	-4.0994267	-0.0001814
H	2.7307018	1.1071740	0.0026791
H	4.1008465	3.0498876	0.0003126
H	5.8154615	2.7873027	-0.0028286
N	1.9504348	6.6755400	-0.0017551
C	3.1583507	6.0103319	-0.0000230
N	3.0075195	4.7143642	0.0017323
C	1.6417751	4.5114673	0.0011687
C	0.8857126	3.3014674	0.0025946
O	1.3048449	2.1484420	0.0046224
N	-0.4942041	3.5603212	0.0014740
C	-1.0619015	4.8082691	-0.0009083
N	-2.4020441	4.8879856	-0.0025095
N	-0.3586513	5.9335289	-0.0021220
C	0.9632569	5.7222632	-0.0009984
H	4.0994267	6.5366306	-0.0001814
H	-1.1071740	2.7307018	0.0026791
H	-3.0498876	4.1008465	0.0003126
H	-2.7873027	5.8154615	-0.0028286

N	-6.6755400	1.9504348	-0.0017551
C	-6.0103319	3.1583507	-0.0000230
N	-4.7143642	3.0075195	0.0017323
C	-4.5114673	1.6417751	0.0011687
C	-3.3014674	0.8857126	0.0025946
O	-2.1484420	1.3048449	0.0046224
N	-3.5603212	-0.4942041	0.0014740
C	-4.8082691	-1.0619015	-0.0009083
N	-4.8879856	-2.4020441	-0.0025095
N	-5.9335289	-0.3586513	-0.0021220
C	-5.7222632	0.9632569	-0.0009984
H	-6.5366306	4.0994267	-0.0001814
H	-2.7307018	-1.1071740	0.0026791
H	-4.1008465	-3.0498876	0.0003126
H	-5.8154615	-2.7873027	-0.0028286
H	7.6706980	-1.8022199	-0.0032574
H	-1.8022199	-7.6706980	-0.0032574
H	-7.6706980	1.8022199	-0.0032574
H	1.8022199	7.6706980	-0.0032574
C	-1.6778918	2.7170760	3.4934693
C	-0.3326830	2.3821839	3.4456704
C	4.2059496	-1.6875345	3.4892426
C	-2.2173558	4.0275304	3.7351211
C	-3.5996552	3.9600958	3.6721420
C	-3.9485903	2.6056276	3.4592222
N	-2.7823484	1.8547613	3.3480177
N	5.5920940	-1.8796522	3.4335254
C	5.8688041	-3.2379237	3.5842864
C	4.6467452	-3.9212636	3.7639231
C	3.6276346	-2.9754384	3.7165956
C	-5.1868695	1.9603575	3.3913493
C	-5.2398936	0.5929687	3.2644422
C	-4.0417217	-0.1453474	3.2087210
C	-2.8371900	0.4952133	3.2455420
C	6.5862248	-0.9734026	3.2096748
C	7.8890658	-1.3784991	3.1940725
C	8.2101034	-2.7435020	3.3704315
C	7.2040020	-3.6623810	3.5506036
C	-1.4230847	5.2269814	4.0326973
C	2.1974241	-3.2302316	3.9595574
C	-4.5946398	5.0708976	3.7485247
C	4.5273916	-5.3892236	4.0111032
C	-1.7811508	6.0551771	5.1104395
C	-1.0468803	7.1795349	5.4279402
C	0.0758541	7.5269762	4.6680565
C	0.4468272	6.7208468	3.5921506
C	-0.2987839	5.5878247	3.2904662
C	1.4774481	-2.4893064	4.9108452

C	0.1196572	-2.6687781	5.0774185
C	-0.5695774	-3.6045696	4.2951381
C	0.1382654	-4.3892374	3.3865121
C	1.5083536	-4.1974908	3.2341354
C	0.3103917	1.2195676	3.0466036
C	1.6735903	1.0457307	3.2307563
C	2.2989973	-0.1840167	3.0531976
C	3.6207991	-0.4211406	3.3657201
O	0.7265657	8.6513203	5.0535801
O	-1.9114388	-3.6697408	4.4925186
C	1.8663211	9.0728487	4.3181092
C	-2.6645304	-4.6132248	3.7360023
H	0.3134073	3.1810657	3.7916437
H	-6.0850487	2.5573656	3.4544724
H	-6.1913715	0.0835437	3.2018543
H	-4.0512115	-1.2214346	3.1213967
H	-1.9068012	-0.0376477	3.2043249
H	6.2900396	0.0457131	3.0198084
H	8.6609004	-0.6410883	3.0271069
H	9.2438334	-3.0610009	3.3525546
H	7.4174836	-4.7156651	3.6685578
H	-4.1126701	6.0344221	3.5884071
H	-5.3655636	4.9465698	2.9846282
H	-5.0972117	5.1092602	4.7194494
H	3.5770324	-5.6250751	4.4888691
H	4.5734826	-5.9584982	3.0778128
H	5.3301264	-5.7495254	4.6576762
H	-2.6372835	5.7954920	5.7198602
H	-1.3179494	7.8071795	6.2664845
H	1.3016225	6.9624400	2.9779507
H	-0.0043213	4.9893267	2.4429236
H	1.9921428	-1.7499979	5.5101522
H	-0.4351685	-2.0925152	5.8056601
H	-0.3579488	-5.1283270	2.7759074
H	2.0415391	-4.7880226	2.5035302
H	-0.2260127	0.4054829	2.5830398
H	2.2581090	1.8820904	3.6070730
H	1.6917104	-1.0074566	2.7063469
H	4.2540030	0.4428021	3.5486259
H	2.2151430	9.9814920	4.8024682
H	2.6605965	8.3201362	4.3451956
H	1.6073046	9.2954272	3.2776328
H	-3.6943613	-4.5080761	4.0690055
H	-2.6044089	-4.4042811	2.6652123
H	-2.3272399	-5.6357843	3.9298043

Table S11.- Cartesian coordinates of the **C5@G4** (cis) complexes

N	-1.8382209	-6.6252078	-1.6247425
C	-3.0461368	-5.9599997	-1.6230104
N	-2.8953056	-4.6640320	-1.6212551
C	-1.5295612	-4.4611351	-1.6218187
C	-0.7734987	-3.2511352	-1.6203928
O	-1.1926310	-2.0981098	-1.6183650
N	0.6064180	-3.5099890	-1.6215134
C	1.1741154	-4.7579369	-1.6238957
N	2.5142580	-4.8376534	-1.6254969
N	0.4708652	-5.8831967	-1.6251094
C	-0.8510430	-5.6719310	-1.6239858
H	-3.9872128	-6.4862984	-1.6231688
H	1.2193879	-2.6803696	-1.6203083
H	3.1621015	-4.0505143	-1.6226748
H	2.8995166	-5.7651293	-1.6258160
N	6.7877539	-1.9001026	-1.6247425
C	6.1225458	-3.1080185	-1.6230104
N	4.8265781	-2.9571873	-1.6212551
C	4.6236812	-1.5914429	-1.6218187
C	3.4136813	-0.8353804	-1.6203928
O	2.2606559	-1.2545127	-1.6183650
N	3.6725351	0.5445363	-1.6215134
C	4.9204830	1.1122337	-1.6238957
N	5.0001995	2.4523763	-1.6254969
N	6.0457428	0.4089835	-1.6251094
C	5.8344771	-0.9129247	-1.6239858
H	6.6488445	-4.0490945	-1.6231688
H	2.8429157	1.1575062	-1.6203083
H	4.2130604	3.1002198	-1.6226748
H	5.9276754	2.8376349	-1.6258160
N	2.0626487	6.7258722	-1.6247425
C	3.2705646	6.0606641	-1.6230104
N	3.1197334	4.7646964	-1.6212551
C	1.7539890	4.5617995	-1.6218187
C	0.9979265	3.3517996	-1.6203928
O	1.4170588	2.1987742	-1.6183650
N	-0.3819902	3.6106534	-1.6215134
C	-0.9496876	4.8586013	-1.6238957
N	-2.2898302	4.9383178	-1.6254969
N	-0.2464374	5.9838611	-1.6251094
C	1.0754708	5.7725954	-1.6239858
H	4.2116406	6.5869628	-1.6231688
H	-0.9949601	2.7810340	-1.6203083
H	-2.9376737	4.1511787	-1.6226748
H	-2.6750888	5.8657937	-1.6258160

N	-6.5633261	2.0007670	-1.6247425
C	-5.8981180	3.2086829	-1.6230104
N	-4.6021503	3.0578517	-1.6212551
C	-4.3992534	1.6921073	-1.6218187
C	-3.1892535	0.9360448	-1.6203928
O	-2.0362281	1.3551771	-1.6183650
N	-3.4481073	-0.4438719	-1.6215134
C	-4.6960552	-1.0115693	-1.6238957
N	-4.7757717	-2.3517119	-1.6254969
N	-5.8213150	-0.3083191	-1.6251094
C	-5.6100493	1.0135891	-1.6239858
H	-6.4244167	4.1497589	-1.6231688
H	-2.6184879	-1.0568418	-1.6203083
H	-3.9886326	-2.9995554	-1.6226748
H	-5.7032476	-2.7369705	-1.6258160
H	7.7829119	-1.7518877	-1.6262448
H	-1.6900060	-7.6203658	-1.6262448
H	-7.5584841	1.8525521	-1.6262448
H	1.9144338	7.7210302	-1.6262448
C	-3.2161731	-2.1684278	1.9322121
C	-2.1506225	-1.2707835	1.9656780
C	3.6801461	0.7235013	1.8925458
C	-4.6019034	-1.8527004	2.1272899
C	-5.3496669	-3.0184284	2.0404353
C	-4.4420507	-4.0821209	1.8408765
N	-3.1519721	-3.5611096	1.7641717
N	4.6300531	-0.3035458	1.7554072
C	5.9044735	0.2509854	1.8391649
C	5.7813155	1.6488170	2.0094720
C	4.4271132	1.9346491	2.0748314
C	-4.6273746	-5.4672920	1.7720526
C	-3.5376540	-6.2975629	1.6689722
C	-2.2418700	-5.7436205	1.6332065
C	-2.0712664	-4.3911361	1.6782994
C	4.4631610	-1.6563245	1.6774714
C	5.5470049	-2.4848761	1.6644394
C	6.8494355	-1.9496068	1.7179514
C	7.0218222	-0.5893222	1.7990313
C	-5.1408443	-0.5024403	2.3394068
C	3.8298232	3.2599990	2.2846296
C	-6.8341644	-3.1864229	2.0835700
C	6.9425385	2.5878205	2.0426792
C	-6.1272080	-0.2730041	3.3091453
C	-6.6777453	0.9854836	3.4822659
C	-6.2490071	2.0520762	2.6832843
C	-5.2525745	1.8420417	1.7257213
C	-4.7107677	0.5795289	1.5637985
C	2.7855618	3.7234414	1.4773861

C	2.2286731	4.9735082	1.6864035
C	2.7163449	5.7959239	2.7049511
C	3.7617378	5.3455811	3.5195893
C	4.3062099	4.0911187	3.3083469
C	-0.8253762	-1.3937097	1.5815876
C	0.0882955	-0.3725330	1.8259043
C	1.4496571	-0.4380721	1.5534348
C	2.2947544	0.5997759	1.9182710
H	-2.4310085	-0.2993211	2.3582392
H	-5.6326797	-5.8600340	1.8328937
H	-3.6686105	-7.3706874	1.6333007
H	-1.3671670	-6.3728391	1.5613682
H	-1.1000794	-3.9334650	1.6695779
H	3.4552764	-2.0255437	1.6417954
H	5.3835532	-3.5497341	1.5971016
H	7.7055172	-2.6104013	1.7041571
H	8.0054913	-0.1456056	1.8572210
H	-7.3391744	-2.2592218	1.8162247
H	-7.1596067	-3.9583769	1.3835855
H	-7.1843756	-3.4811421	3.0768749
H	6.6221611	3.6110679	1.8524268
H	7.4526528	2.5744757	3.0101691
H	7.6772615	2.3172558	1.2815973
H	-6.4562854	-1.0867400	3.9408321
H	-7.4392779	1.1499167	4.2325651
H	-4.9203755	2.6579847	1.0984144
H	-3.9694213	0.4215148	0.7981564
H	2.4309733	3.1135707	0.6587036
H	1.4288016	5.3239556	1.0494734
H	4.1357676	5.9785098	4.3130461
H	5.1003823	3.7405214	3.9541307
H	-0.4654805	-2.2676811	1.0595613
H	-0.2841570	0.5276042	2.3096457
H	1.8145967	-1.3051261	1.0214794
H	1.8013249	1.4916740	2.2891745
C	-6.8364430	3.3473998	2.8254065
C	2.1597480	7.0990036	2.8944210
N	-7.3141138	4.3920807	2.9188861
N	1.7143907	8.1533175	3.0292797

Table S12.- Cartesian coordinates of the **C5@G4** (trans) complexes

N	-1.9504348	-6.6755400	-0.0017551
C	-3.1583507	-6.0103319	-0.0000230
N	-3.0075195	-4.7143642	0.0017323
C	-1.6417751	-4.5114673	0.0011687
C	-0.8857126	-3.3014674	0.0025946
O	-1.3048449	-2.1484420	0.0046224
N	0.4942041	-3.5603212	0.0014740
C	1.0619015	-4.8082691	-0.0009083
N	2.4020441	-4.8879856	-0.0025095
N	0.3586513	-5.9335289	-0.0021220
C	-0.9632569	-5.7222632	-0.0009984
H	-4.0994267	-6.5366306	-0.0001814
H	1.1071740	-2.7307018	0.0026791
H	3.0498876	-4.1008465	0.0003126
H	2.7873027	-5.8154615	-0.0028286
N	6.6755400	-1.9504348	-0.0017551
C	6.0103319	-3.1583507	-0.0000230
N	4.7143642	-3.0075195	0.0017323
C	4.5114673	-1.6417751	0.0011687
C	3.3014674	-0.8857126	0.0025946
O	2.1484420	-1.3048449	0.0046224
N	3.5603212	0.4942041	0.0014740
C	4.8082691	1.0619015	-0.0009083
N	4.8879856	2.4020441	-0.0025095
N	5.9335289	0.3586513	-0.0021220
C	5.7222632	-0.9632569	-0.0009984
H	6.5366306	-4.0994267	-0.0001814
H	2.7307018	1.1071740	0.0026791
H	4.1008465	3.0498876	0.0003126
H	5.8154615	2.7873027	-0.0028286
N	1.9504348	6.6755400	-0.0017551
C	3.1583507	6.0103319	-0.0000230
N	3.0075195	4.7143642	0.0017323
C	1.6417751	4.5114673	0.0011687
C	0.8857126	3.3014674	0.0025946
O	1.3048449	2.1484420	0.0046224
N	-0.4942041	3.5603212	0.0014740
C	-1.0619015	4.8082691	-0.0009083
N	-2.4020441	4.8879856	-0.0025095
N	-0.3586513	5.9335289	-0.0021220
C	0.9632569	5.7222632	-0.0009984
H	4.0994267	6.5366306	-0.0001814
H	-1.1071740	2.7307018	0.0026791
H	-3.0498876	4.1008465	0.0003126
H	-2.7873027	5.8154615	-0.0028286

N	-6.6755400	1.9504348	-0.0017551
C	-6.0103319	3.1583507	-0.0000230
N	-4.7143642	3.0075195	0.0017323
C	-4.5114673	1.6417751	0.0011687
C	-3.3014674	0.8857126	0.0025946
O	-2.1484420	1.3048449	0.0046224
N	-3.5603212	-0.4942041	0.0014740
C	-4.8082691	-1.0619015	-0.0009083
N	-4.8879856	-2.4020441	-0.0025095
N	-5.9335289	-0.3586513	-0.0021220
C	-5.7222632	0.9632569	-0.0009984
H	-6.5366306	4.0994267	-0.0001814
H	-2.7307018	-1.1071740	0.0026791
H	-4.1008465	-3.0498876	0.0003126
H	-5.8154615	-2.7873027	-0.0028286
H	7.6706980	-1.8022199	-0.0032574
H	-1.8022199	-7.6706980	-0.0032574
H	-7.6706980	1.8022199	-0.0032574
H	1.8022199	7.6706980	-0.0032574
C	-4.4161475	3.6241387	3.2211144
C	-3.0828752	4.0208283	3.1940281
C	2.8559448	2.4326998	3.3254108
C	-5.5502580	4.4953353	3.2320483
C	-6.6992601	3.7556839	3.4307151
C	-6.3097706	2.3923653	3.4898576
N	-4.9274503	2.3196965	3.3518272
N	4.1879819	2.8668157	3.1966126
C	5.0351094	1.7699891	3.2896221
C	4.2431968	0.6080588	3.4670458
C	2.9185713	1.0083934	3.4816460
C	-7.0518178	1.2138124	3.5998006
C	-6.4126513	-0.0029451	3.5402814
C	-5.0190494	-0.0507516	3.3474057
C	-4.3032166	1.1073473	3.2428780
C	4.6788591	4.1246218	3.0179805
C	6.0242221	4.3235036	2.9063276
C	6.9122914	3.2287529	2.9837919
C	6.4155035	1.9622647	3.1755145
C	-5.4720014	5.9545293	3.0371528
C	1.7465777	0.1115140	3.5208116
C	-8.1097920	4.2368619	3.5233789
C	4.7863148	-0.7807020	3.5454414
C	-5.0655177	6.4663279	1.8003069
C	-4.9796464	7.8323109	1.5885559
C	-5.3018123	8.7167186	2.6241006
C	-5.7234434	8.2152341	3.8599667
C	-5.8088617	6.8456214	4.0588062
C	0.8512611	0.1133951	2.4484352

C	-0.2942545	-0.6553948	2.4809066
C	-0.5583280	-1.4612781	3.5931623
C	0.3500750	-1.5051011	4.6573898
C	1.4958980	-0.7229495	4.6147150
C	-1.9357034	3.2935218	3.4659070
C	-0.6428133	3.7867905	3.3134125
C	0.4628474	2.9877942	3.5752389
C	1.7748903	3.3137278	3.2795349
C	-1.7775243	-2.2064602	3.6306932
N	-2.7885040	-2.7608178	3.6429765
H	-2.9473508	5.0698808	2.9547419
H	-8.1240190	1.2847877	3.7188880
H	-6.9752796	-0.9228094	3.6168972
H	-4.4998735	-0.9949852	3.2631580
H	-3.2497345	1.1128600	3.0307844
H	3.9629469	4.9276114	2.9610089
H	7.9778426	3.3910950	2.8952988
H	7.0661088	1.1020784	3.2339799
H	-8.1754957	5.2997609	3.2951270
H	-8.7592588	3.7011014	2.8259865
H	-8.5212566	4.0850094	4.5250098
H	5.5848023	-0.9296851	2.8170319
H	5.1983331	-0.9945994	4.5357900
H	4.0077664	-1.5142759	3.3413743
H	-4.8326846	5.7738306	1.0030393
H	-5.9754754	8.9008423	4.6576648
H	-6.1250697	6.4607106	5.0196385
H	1.0272742	0.7391677	1.5880806
H	-0.9617126	-0.6440440	1.6344555
H	0.1497550	-2.1357202	5.5131681
H	2.1851750	-0.7374625	5.4492857
H	-2.0116151	2.2757263	3.8218124
H	-0.5018692	4.7907967	2.9200364
H	0.2582019	2.0052458	3.9724174
H	1.9687182	4.3279738	2.9459175
H	6.3940229	5.3285370	2.7639245
H	-4.6710330	8.2238117	0.6282898
C	-5.1960186	10.1278086	2.4175487
N	-5.1015102	11.2636681	2.2490733

Table S13. Values of binding affinity scores (ΔE, in kcal·mol^{-1}) for complexes involving compounds **C1** to **C5-Sulf** and a duplex DNA and their location.

Ligand	Affinity (kcal/mol)	Location
**C1**	-7.0	Major groove
**C2**	-8.3	Minor groove
**C3**	-8.5	Minor groove
**C4**	-8.8	Minor groove
**C5**	-10.2	Minor groove
**C5-Sulf**	-7.60	Minor groove

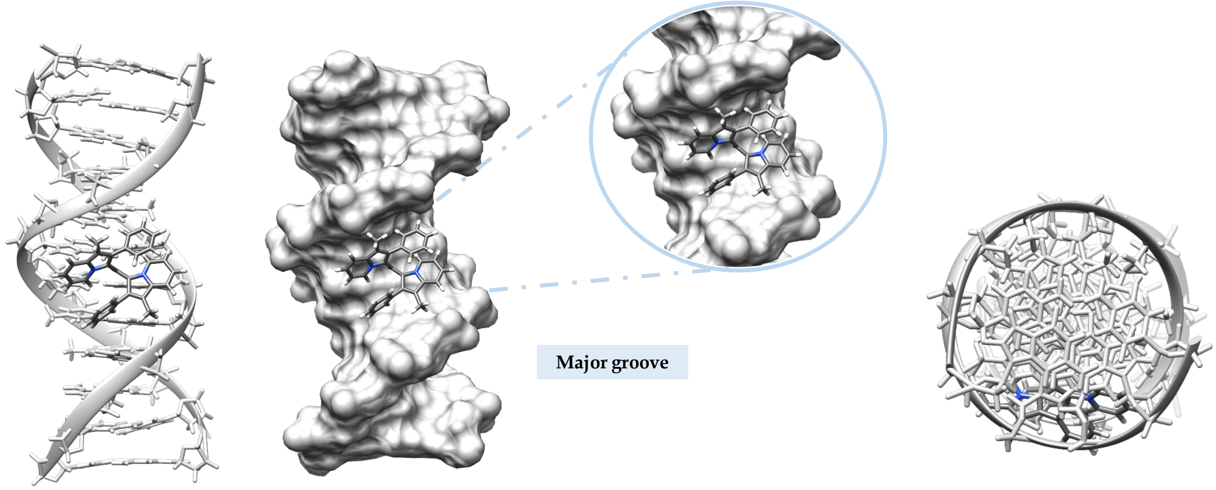

Figure S38. Molecular docking results with Autodock Vina of **C1**.

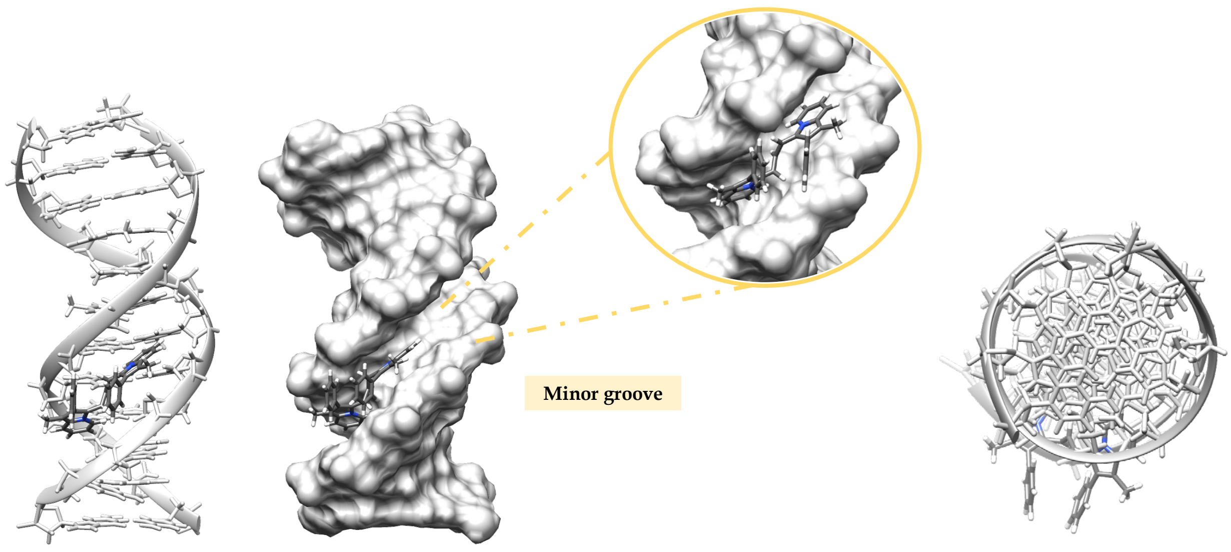

Figure S39. Molecular docking results with Autodock Vina of **C2**.

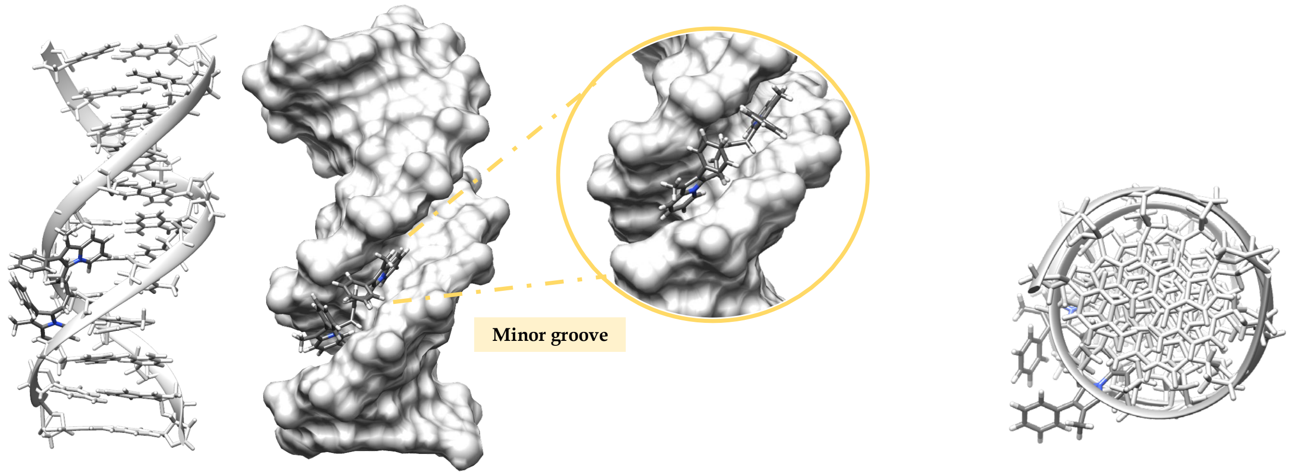

Figure S40. Molecular docking results with Autodock Vina of **C3**.

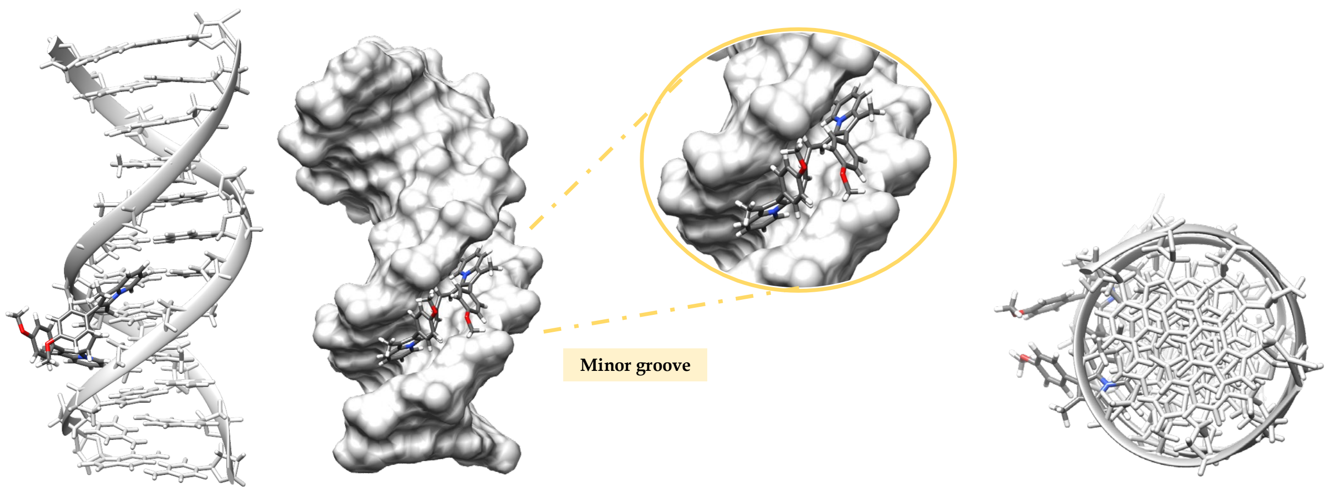

Figure S41. Molecular docking results with Autodock Vina of **C4**.

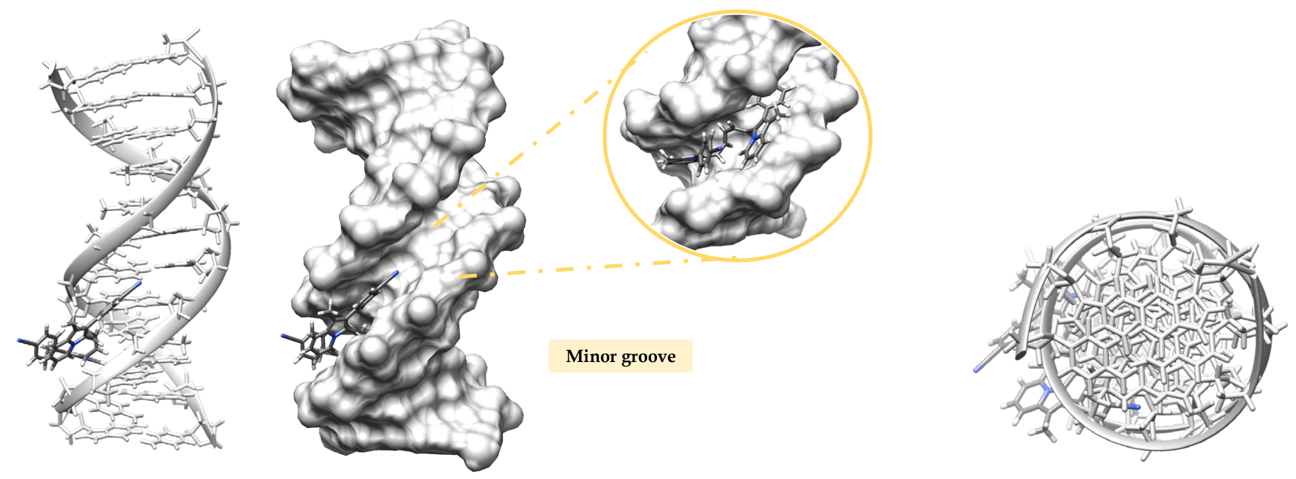

Figure S42. Molecular docking results with Autodock Vina of **C5**.

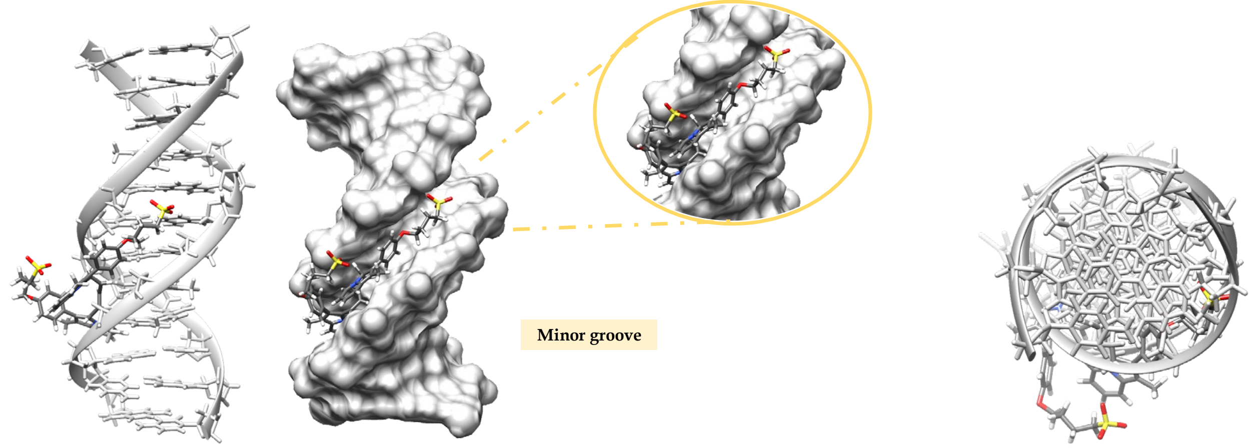

Figure S43. Molecular docking results with Autodock Vina of **C5-Sulfur**.

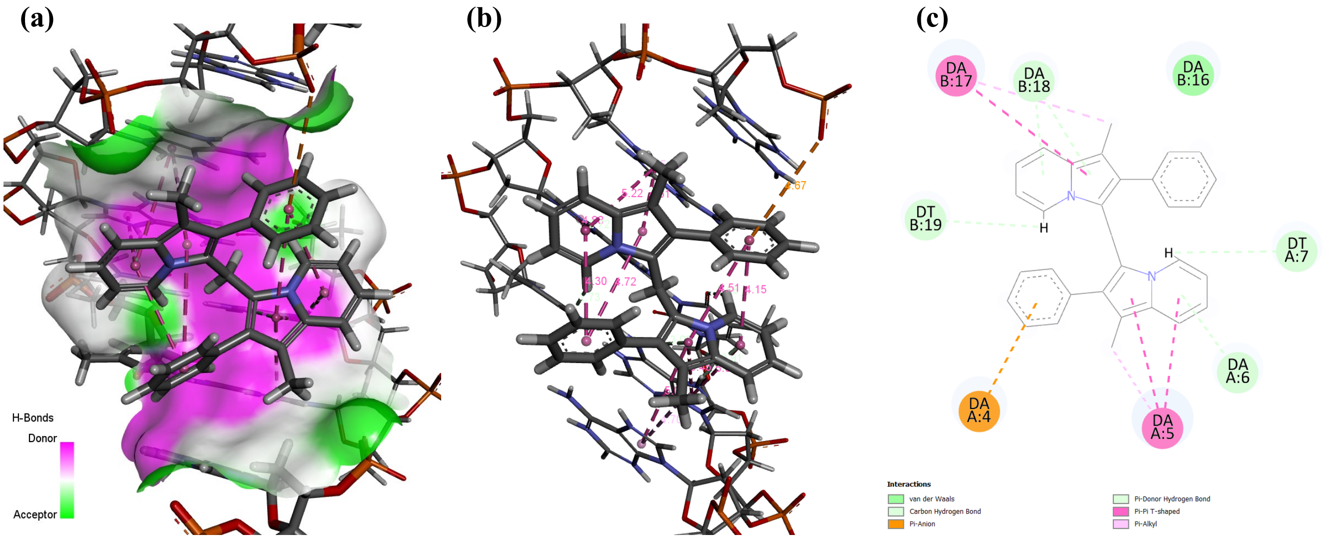

Figure S44. 3D and 2D molecular docking results with Autodock Vina of **C1**. Represented (a) stick model surrounded by surface to show the desirable site of H-bond donor and acceptor, (b) main interaction distances and (c) molecular interactions.

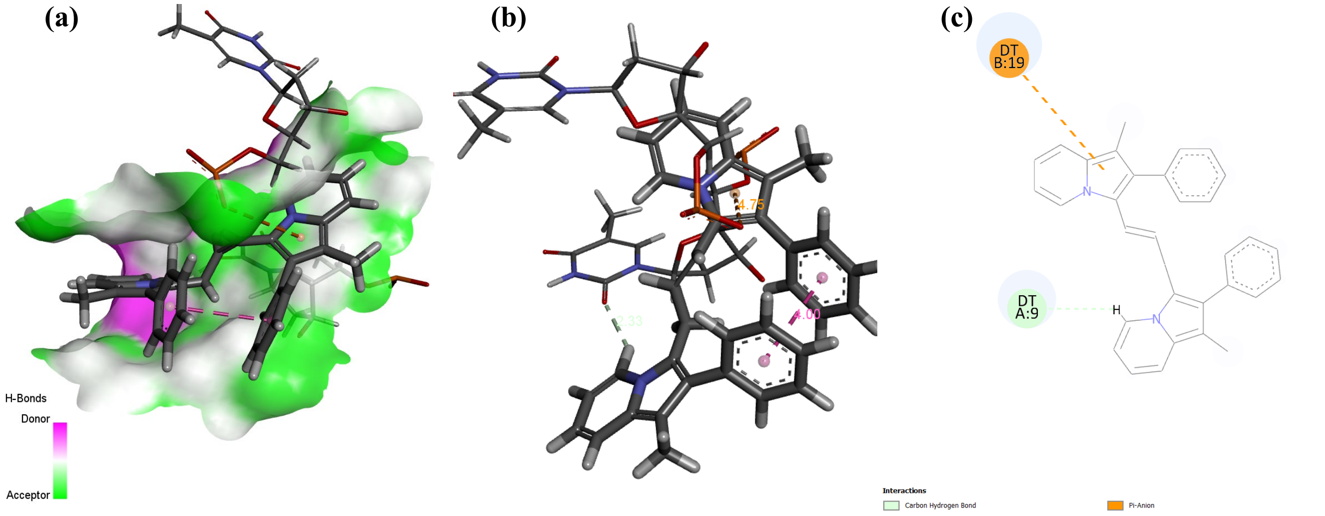

Figure S45. 3D and 2D molecular docking results with Autodock Vina of **C2**. Represented (a) stick model surrounded by surface to show the desirable site of H-bond donor and acceptor, (b) main interaction distances and (c) molecular interactions

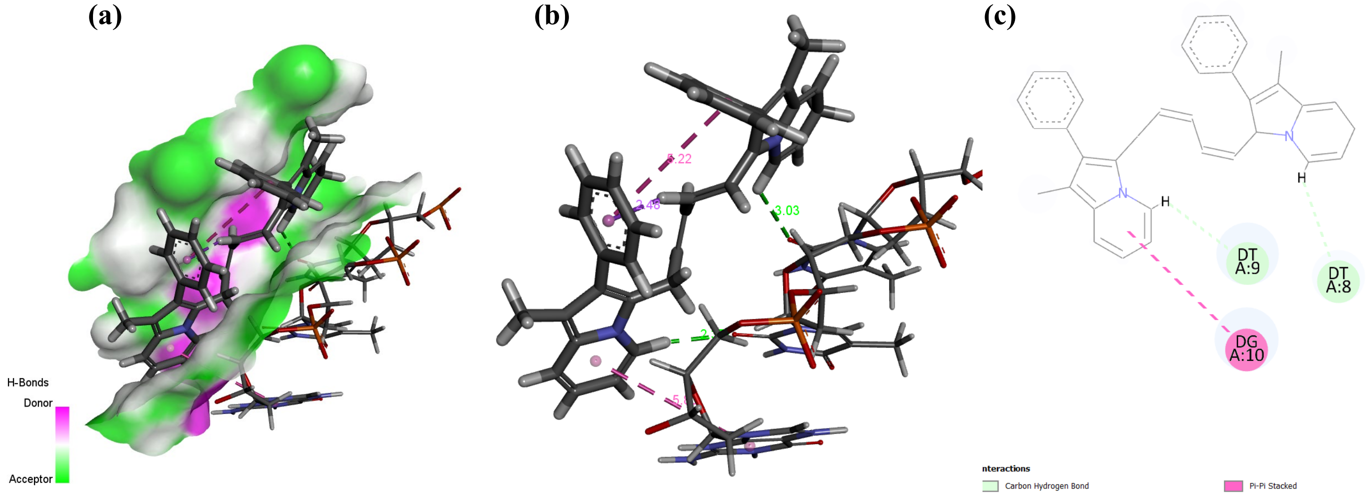

Figure S46. 3D and 2D molecular docking results with Autodock Vina of **C3**. Represented (a) stick model surrounded by surface to show the desirable site of H-bond donor and acceptor, (b) main interaction distances and (c) molecular interactions.

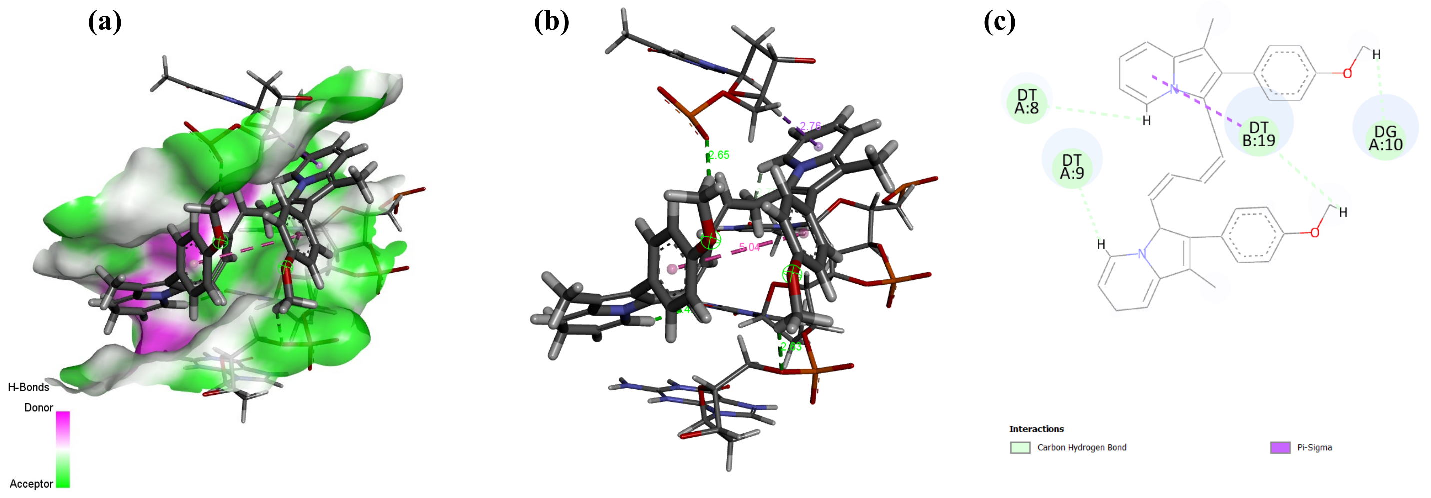

Figure S47. 3D and 2D molecular docking results with Autodock Vina of **C4**. Represented (a) stick model surrounded by surface to show the desirable site of H-bond donor and acceptor, (b) main interaction distances and (c) molecular interactions.

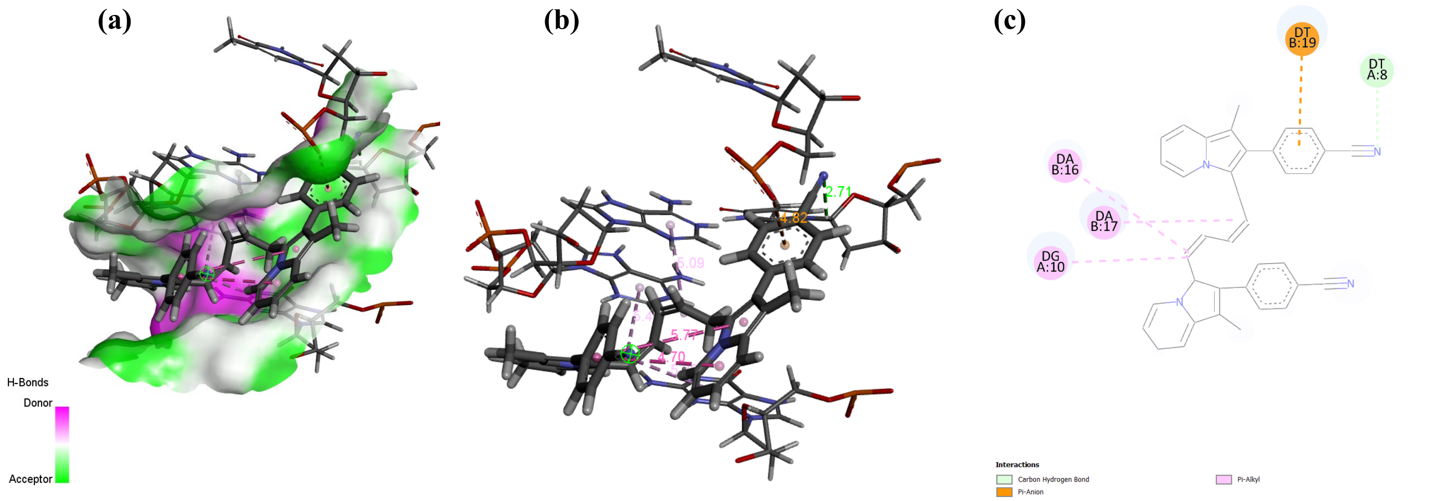

Figure S48. 3D and 2D molecular docking results with Autodock Vina of **C5**. Represented (a) stick model surrounded by surface to show the desirable site of H-bond donor and acceptor, (b) main interaction distances and (c) molecular interactions.

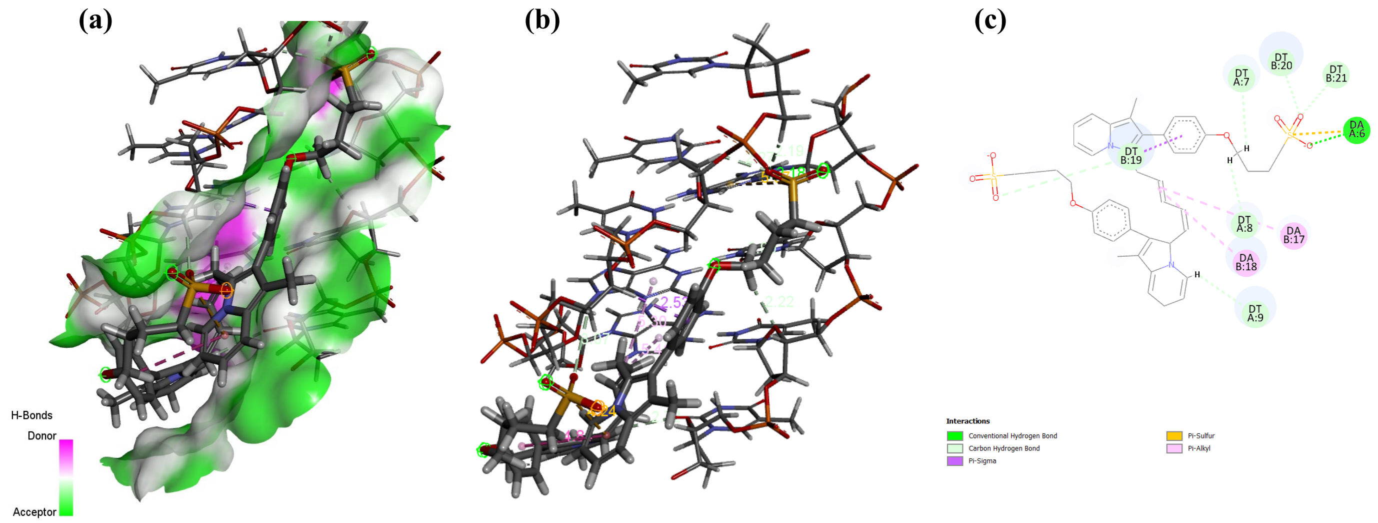

Figure S49. 3D and 2D molecular docking results with Autodock Vina of **C5-Sulfur**. Represented (a) stick model surrounded by surface to show the desirable site of H-bond donor and acceptor, (b) main interaction distances and (c) molecular interactions.

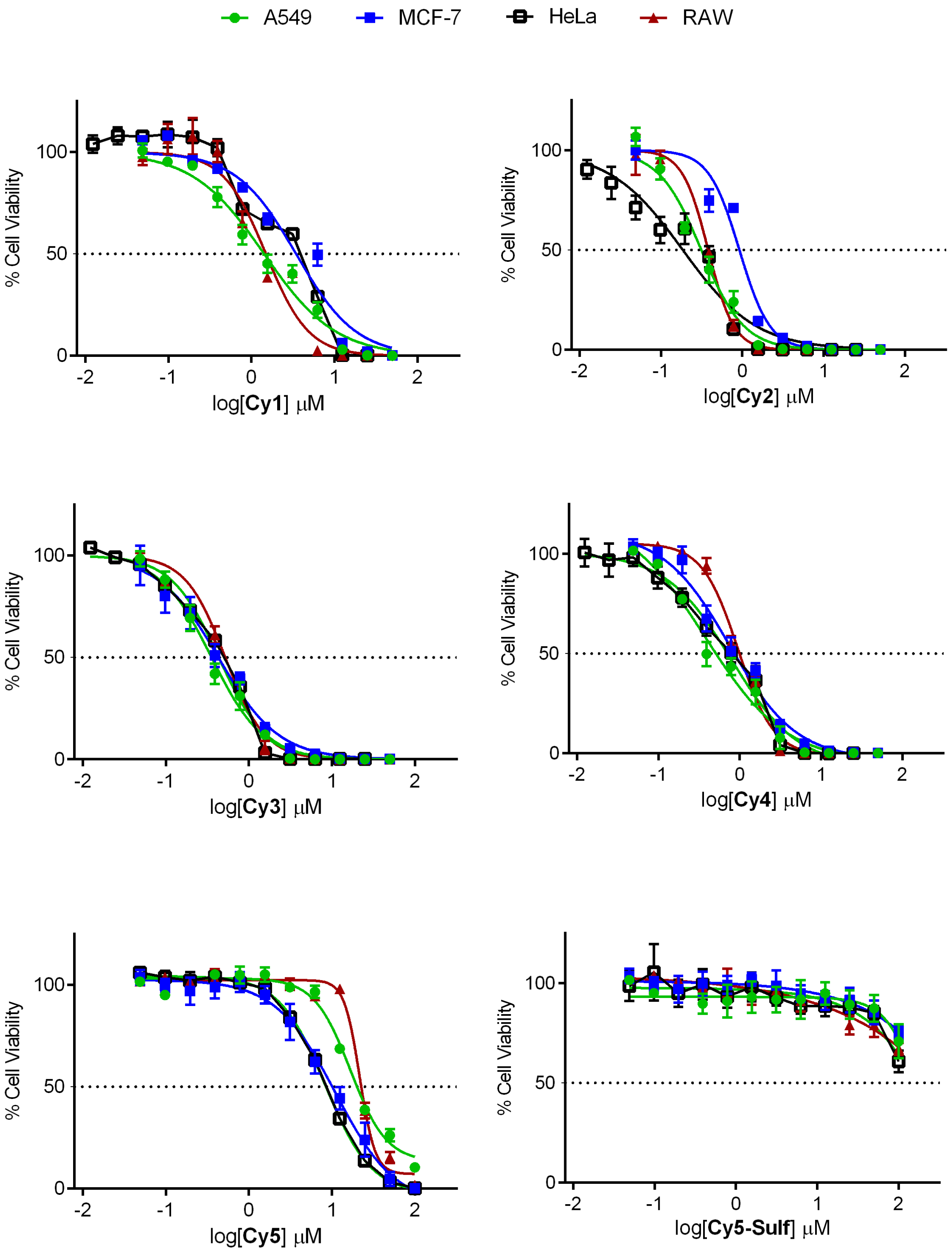

Figure S50.- Dose-response curves of the indolizine cyanine ligands in selected cell lines.
